# On elastic deformations of cylindrical bodies under the influence of the gravitational field

**DOI:** 10.12688/openreseurope.17329.1

**Published:** 2024-05-10

**Authors:** Hamed Barzegar, Piotr T. Chruściel, Florian Steininger

**Affiliations:** 1 Centre de Recherche Astrophysique de Lyon, Universite Claude Bernard Lyon 1, Villeurbanne, Auvergne-Rhône-Alpes, 69622, France; 2 Faculty of Physics, University of Vienna, Vienna, Vienna, 1090, Austria

**Keywords:** linear elasticity, GRAVITES, waveguides, Airy stress, Michell solution

## Abstract

**Background:**

Large elastic deformations of gravitating cylindrical bodies play a significant role in everyday life. On the other hand, tiny such deformations are relevant for state-of-the art experiments, as they affect the physical properties of materials under consideration, impacting wave propagation. This is of key importance for a recently planned experiment to explore the influence of the gravitational field on entangled photons propagating in waveguides. The purpose of this work is to determine this influence.

**Methods:**

We use the methods of linear elasticity, including thermoelasticity, to determine the stresses and strains of the medium. For this, the symmetry of the cylinder allows us to solve the problem by using Mitchell’s solutions of the equations satisfied by the Airy functions. The boundary conditions are implemented by an approximation of the Hertz contact method.

**Results:**

We calculate the displacements, the stresses and strains for several classes of boundary conditions, and give explicit solutions for a number of physically motivated configurations. The influence of the resulting deformations on the planned GRAVITES experiment is determined.

**Conclusions:**

The results are relevant for fiber interferometry experiments sensitive to the effects of the gravitational field on photon propagation. Our calculations give stringent bounds on the environmental variables, which need to be controlled in such experiments.

## 1 Introduction

**Figure 1.1.**
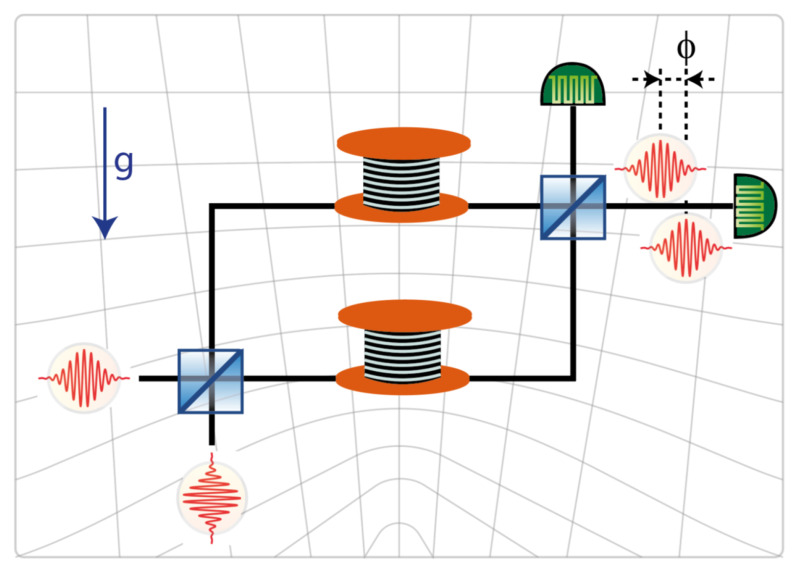
The GRAVITES experiment. The upper arm of the interferometer is moved vertically. The change of the gravitational field due to the change of height affects the propagation of light, resulting in a height-dependent phase shift. Here and unless otherwise indicated, the *y*-axis is aligned vertically, so that the gravitational force acts anti-colinearly along the *y*-axis. © C. Hilweg, reproduced with kind permission of the author.

An experiment is currently being built [ 1] with the aim to measure the effect of the gravitational field on entangled states of photons propagating in an optical fiber. The experiment requires displacing vertically an optical fiber, with circular cross-sections, in a spooled configuration, see Figure 1.1. Such a displacement in the gravitational field of the Earth leads to a minute phaseshift, which is expected to be measurable with the current state-of-the art photonic technology. The displacement is associated with a change of the ambient gravitational acceleration, of temperature, and of atmospheric pressure, leading to an elastic deformation of the fiber. The deformation affects the shape, the length, and the propagation properties of light in the fiber, leading to an additional phaseshift which needs to be determined for a correct interpretation of the results of the experiment. The objective of this work is to develop a framework which can be used to determine this elastic deformation. The resulting effects on the propagation properties of light in the fiber will be determined elsewhere.

**Figure 1.2.**
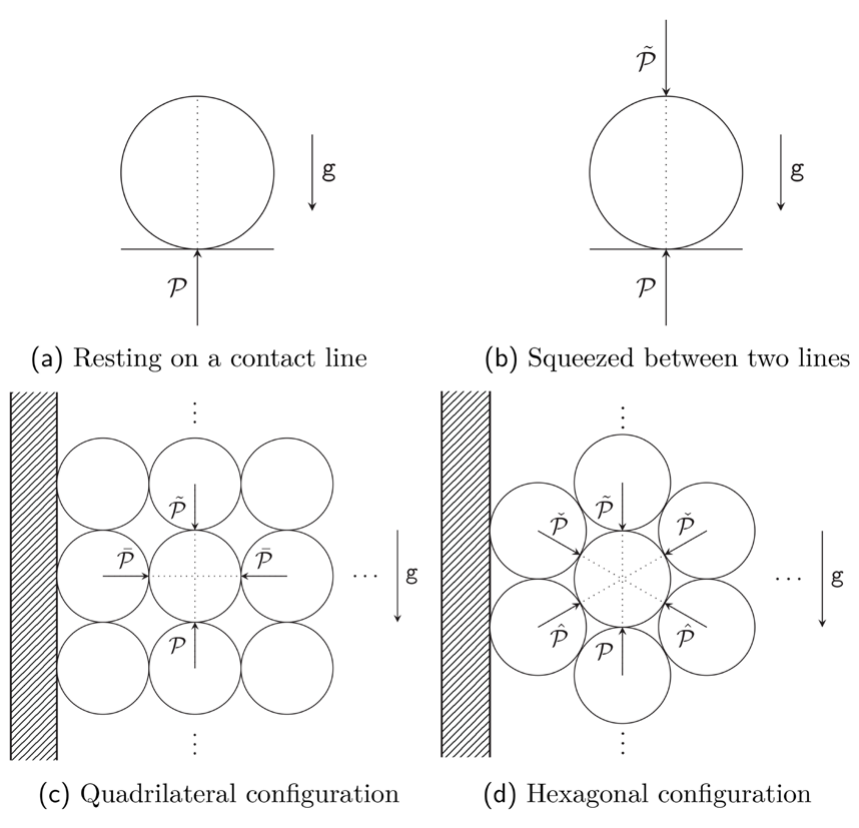
The four main models. The *y*-axis is again along the vertical in all four figures. Figures ( **a**) and ( **b**) show the cross-section of an infinite cylinder resting on a rigid support, with a supplementary pressure from the top in Figure ( **b**). Figures ( **c**) and ( **d**) show the spool of Figure 1.1 as cut by a vertical plane; we assume that the radius of the spool is very large compared to the radius of the waveguide. The circles represent the consecutive returns of the waveguide, exercising pressure on the neighboring strands. The dashed region represents the rigid spool.

The planned configuration is that of a spool with its axis of symmetry aligned vertically. Since the radius of the spool is very large compared to the radius of the fiber, we ignore the spooling and consider a very long elastic cylinder. We then consider several models for the problem at hand:

As a first model we start with a cylindrical waveguide resting on a horizontal contact line (see Figure 1.2a and Section 4.1.1). The next model is a configuration where the waveguide is squeezed between two contact lines, to take into account the pressure arising from the layers pressing from above (see Figure 1.2b and Section 4.1.2). The results obtained in both cases are unacceptable, with an infinite deformation at each contact line; this is of course a well known problem of such models (cf. [ 2, Chapter 8.4.7]). However, the model appears useful for its simplicity, we will return to this shortly.

To avoid the above problems we pass to a Hertz-contact-type calculation, where we first analyse a configuration with the waveguide resting on a rigid support, followed by one where the waveguides are stacked upon each other; in the last case the influence of the upper layer on a lower one is modelled by contact with a rigid plane. There arises a parameter describing the pressure from the waveguides stacked above the section of the fiber under consideration. We leave this parameter free in our calculations, its value can be determined by the number of layers and windings of the spool whenever a specific configuration is considered.

The above takes into account the vertical neighbours of any given section of the fiber, but ignores the side neighbours. To address this we consider two further configurations, where each fiber has four neighbours as in Figure 1.2c (see Section 4.2.3), or each fiber has six neighbours as in Figure 1.2d and Section 4.2.4. The last model seems to us to provide the best approximation to the problem at hand. In such models new parameters arise, associated with presence of new neighbours. We leave these parameters free again, and show in Appendix A how one of these parameters can be determined for each layer in the quadrilateral-contact case.

Our results show that in all models the relative deformations are similar, for the practical purposes of our interest, close enough to the center of waveguide, where the guiding core resides. Therefore we expect that the first, simplest model, will be sufficient for the applications we have in mind. We plan to return to this question in a future full treatment of the influence of small elastic deformations on the dispersion relation in optical fibers.

We include a constant ambient pressure term, as well as temperature effects in all configurations, in order to model possible variations of the environment.

## 2 Waveguide deformation in earth’s gravity

The unperturbed waveguide is modelled as a very long, homogeneous cylinder with radius *a*, length *L* and density *ρ*, supported by a rigid plane with gravity acting as a body force via a constant gravitational acceleration g. We take *U* = {( *r, θ, z*) : 0 *< r ≤ a, –π < θ ≤ π,* 0 *≤ z ≤ L*} to be the interior of the waveguide and write *∂U* for the surface *r* = *a*. Note that *a ⪡ L*.

It is known that in isotropic and homogeneous materials the generalized Hooke’s law in three dimensions yields the following stress-strain relation (cf., e.g., [ 2, Eq. (4.2.7)] and [ 3, Eq. (4.6)]),


$$\;\sigma_{ij}=\lambda \epsilon_{kk}\delta_{ij}+2\mu \epsilon_{ij},\;(2.1)$$

where *λ* and *µ* are Lamé’s first and second parameters of the material, respectively, with *µ* being called the shear modulus which is sometimes denoted by *G* in the literature; repeated indices are summed over unless explicitly indicated otherwise. Here, and elsewhere, all tensors are expressed in orthonormal frames. With *σ _kk_* = (3 *λ* + 2 *µ*) *ϵ _kk_* we find


$$\;\epsilon_{ij}=\frac{1+\nu }{E}\sigma_{ij}-\frac{\nu }{E}\sigma_{kk}\delta_{ij},\;(2.2)$$

where *E* := *µ*(3 *λ* + 2 *µ*) */*( *λ* + *µ*) is Young’s modulus and *ν* := *λ/*[2( *λ* + *µ*)] is Poisson’s ratio. Note that *E* = 2 *µ*(1 + *ν*).

When the changes of temperature are *not* negligible, the above needs to be revised as follows: According to [ 2, Section 4.4], in a thermally-isotropic and thermally-linear medium, Equation ( 2.2) should be replaced by


$$\;\epsilon_{ij}=\frac{1+\nu }{E}\sigma_{ij}-\frac{\nu }{E}\sigma_{kk}\delta_{ij}+\alpha (T-T_{0})\delta_{ij},\;(2.3)$$

where *α* is the *coefficient of linear thermal expansion*. This is a consequence of the assumptions of linear thermo-elasticity, for which the strain decomposes into independent thermal and elastic components. Inverting this relation, the stress tensor is given by


$$\;\sigma_{ij}=\lambda \epsilon_{kk}\delta_{ij}+2\mu \epsilon_{ij}-(3\lambda +2\mu )\alpha (T-T_{0})\delta_{ij}.\;(2.4)$$

**Figure 2.1.**
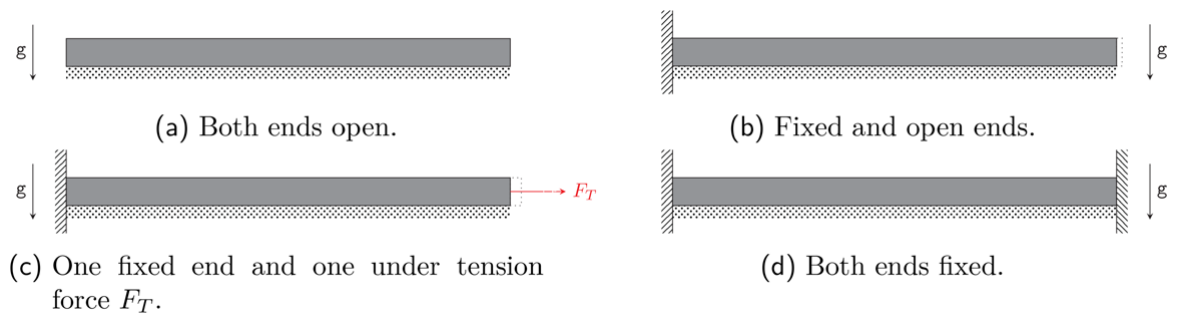
Four models for a very long cylinder with boundary conditions covered by our calculations. Vertical dashed regions represent fixed ends in the *z*-direction, whereas the dotted bars represent rigid supports. The remaining boundaries in the figures can move freely. Note that the model ( **d**) requires the vanishing of the elongation coefficient *κ* of ( 2.5).


We allow the waveguide to stretch in the *z* direction linearly in *z* (compare Figure 2.1 (a)–(c)):

$$\;u_{z}=\kappa z.\;(2.5)$$

All remaining fields are assumed to be *z*-independent. Equation ( 2.5) leads to a non-vanishing *z*-component of the strain tensor


$$\;\epsilon_{ij}=\frac{1}{2}(\partial_{i}u_{j}+\partial_{j}u_{i}),\;(2.6)$$

namely


$$\;\epsilon_{zz}=\kappa ,\;\text{but}\;\epsilon_{xz}=\epsilon_{yz}=0,\;(2.7)$$

giving an additional contribution to the usual plane-strain relations (cf. [ 4] for related considerations).

Having reduced the dimensionality of the problem, the equilibrium equations (cf., e.g., [ 2, Eq. (3.6.4)] or [ 5, Eq. (2.34)]),


$$\;\partial_{j}\sigma_{ij}+F_{i}=0,\;(2.8)$$

with *σ _ij_* the stress tensor and *F _i_* = *−∂ _i_V* the body force due to gravity, where *V* = g *ρy*, simplify to


$$\;\partial_{x}\sigma_{xx}+\partial_{y}\sigma_{xy}-\partial_{x}V=0,\;(2.9)$$

$$\;\partial_{x}\sigma_{xy}+\partial_{y}\sigma_{yy}-\partial_{y}V=0.\;(2.10)$$

Note that the stress in the *z* direction does not necessarily vanish. Indeed, the *zz*-component of ( 2.3) gives


$$\;\kappa =\frac{\sigma_{zz}}{E}-\frac{\nu }{E}(\sigma_{rr}+\sigma_{\theta \theta })+\alpha (T-T_{0}),\;(2.11)$$

so that


$$\;\sigma_{zz}=\nu (\sigma_{xx}+\sigma_{yy})+E\kappa -E\alpha (T-T_{0}).\;(2.12)$$

Adapting our coordinate system to the symmetry of the setup by choosing cylindrical coordinates, from now on tensor components will refer to the following orthonormal frame, which in Cartesian coordinates is given by


$$\;e_{r}\;\text{:=}\;(\cos(\theta ),\;\sin(\theta ),0),\;e_{\theta }\;\text{:=}\;(\text{−}\sin(\theta ),\;\cos(\theta ),0),\;e_{z}\;\text{:=}\;(0,0,1).\;(2.13)$$

In this frame the stress tensor can be expressed in terms of derivatives of the Airy stress function *ϕ* as (cf. [ 6] and, e.g., [ 7, Eqs. (8.12)–(8.13)])


$$\;\sigma_{rr}=\frac{1}{r}\partial_{r}\phi +\frac{1}{r^{2}}\partial_{\theta }^{2}\phi +V,\;(2.14)$$

$$\;\sigma_{\theta \theta }=\partial_{r}^{2}\phi +V,\;(2.15)$$

$$\;\sigma_{r\theta }=\text{−}\partial_{r}(\frac{1}{r}\partial_{\theta }\phi ),\;(2.16)$$

which has to satisfy the compatibility condition (see, e.g., [ 2, Eq. (7.5.5) or [ 5, Eq. (7.17b)], together with [ 2, Eq. (12.3.7)]),

$$\;{\text{Δ}}_{\delta }^{2}\phi =\text{−}\frac{1-2\nu }{1-\nu }{\text{Δ}}_{\delta }V-\frac{E\alpha }{1-\nu }{\text{Δ}}_{\delta }T,\;(2.17)$$

where Δ _*δ*_ is the Laplace operator of the Euclidean metric on ℝ ^2^. (Note that *V* is only defined up to a constant, which can be absorbed by a redefinition of *ϕ*.) In this work we consider a steady-state configuration, which requires Δ *_δ_T* = 0 (cf. [ 2, Section 12.1]). Further, since *V* = g *ρy* we have Δ *_δ_V* = 0 as well, implying that the compatibility conditions reduce to the homogeneous biharmonic equation.

Since the frame { *e _r_, e _θ_, e _z_*} is orthonormal, the strain is related to the stress via ( 2.3):


$$\;\epsilon_{rr}=\frac{1}{2\mu }[(1-\nu )\sigma_{rr}-\nu \sigma_{\theta \theta }]-\nu \kappa +(1+\nu )\alpha (T-T_{0}),\;(2.18)$$

$$\;\epsilon_{\theta \theta }=\frac{1}{2\mu }[(1-\nu )\sigma_{\theta \theta }-\nu \sigma_{rr}]-\nu \kappa +(1+\nu )\alpha (T-T_{0}),\;(2.19)$$

$$\;\epsilon_{r\theta }=\frac{1}{2\mu }\sigma_{r\theta }.\;(2.20)$$

One can now determine the displacement vector *u _i_* from the usual equations, where *u _r_* and *u _θ_* are frame components of *u* (cf., e.g., [ 2, Eq. (7.6.1)])


$$\;\epsilon_{rr}=\partial_{r}u_{r},\;(2.21)$$

$$\;\epsilon_{\theta \theta }=\frac{1}{r}(\partial_{\theta }u_{\theta }+u_{r}),\;(2.22)$$

$$\;\epsilon_{r\theta }=\frac{1}{2}\left(\frac{1}{r}\partial_{\theta }u_{r}+\partial_{r}u_{\theta }-\frac{1}{r}u_{\theta }\right).\;(2.23)$$

**Figure 2.2.**
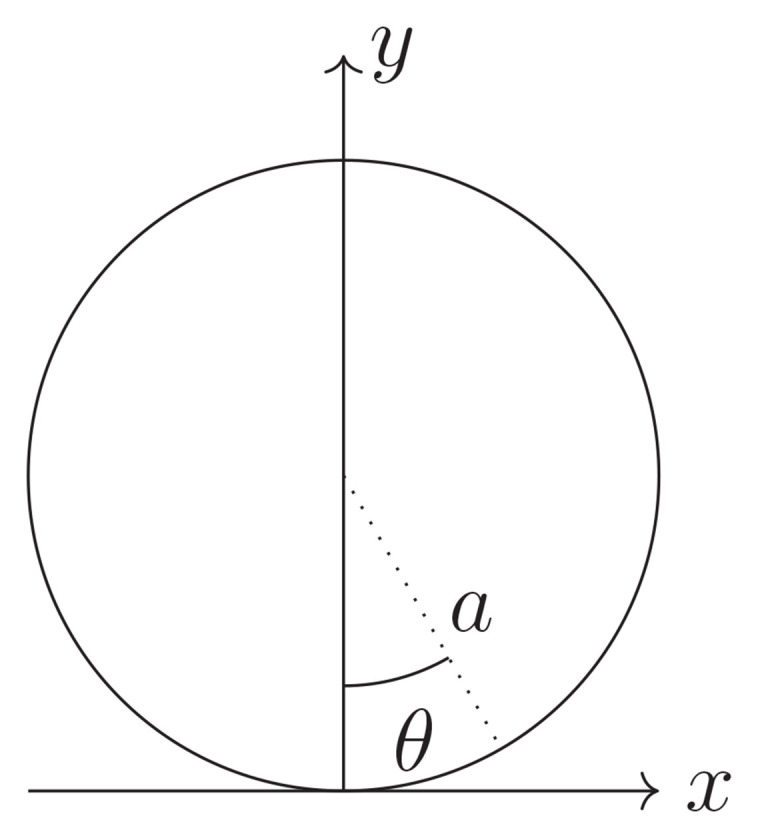
Convention for polar coordinates.

Having compiled all relevant equations from linear elasticity, we set up an adapted coordinate system as in Figure 2.2, with 


r∈(0,a]andθ∈(−π,π].(2.24)

There exists a general solution for ( 2.17) in polar coordinates, denoted by ^1^


$$\;\begin{array}{l} \phi (r,\theta )=A_{0}\;\text{ln}\;r+B_{0}+C_{0}r^{2}\;\text{ln}\;r+D_{0}r^{2}\\ \;+\left(a_{0}\;\text{ln}\;r+b_{0}+c_{0}r^{2}\;\text{ln}\;r+d_{0}r^{2}\right)\theta \\ \;+\left(A_{1}r\;\text{ln}\;r+\frac{B_{1}}{r}+C_{1}r^{3}+D_{1}r+E_{1}r\theta +F_{1}r\theta \;\text{ln}\;r\right)\text{cos(}\theta \text{)}\\ \;+\left(a_{1}r\;\text{ln}\;r+\frac{b_{1}}{r}+c_{1}r^{3}+d_{1}r+e_{1}r\theta +f_{1}r\theta \;\text{ln}\;r\right)\text{sin(}\theta \text{)}\\ \;+\sum_{n\geq 2}[\left(A_{n}r^{n}+B_{n}r^{-n}+C_{n}r^{n+2}+D_{n}r^{2-n}\right)\text{cos(}n\theta \text{)}\\ \;+\left(a_{n}r^{n}+b_{n}r^{-n}+c_{n}r^{n+2}+d_{n}r^{2-n}\right)\text{sin(}n\theta \text{)}],\;(2.25) \end{array}$$

known as the ‘Michell solution’ [ 8].

In practical terms, we are mostly concerned with determining the coefficients in ( 2.25) from the boundary conditions we impose. An immediate simplification can be achieved by only considering solutions which have the mirror symmetry *x* ↦ *−x* (equivalently *θ* ↦ *−θ*), since the forces considered are invariant under this symmetry; see Appendix B for a treatment without imposing mirror symmetry at the outset. Additionally, we require regularity at *r* = 0 and 2 *π*-periodicity in *θ*. Restricting to mirror-symmetric boundary conditions finally leads to a requirement of mirror-symmetry in *σ _rr_* and *σ _θθ_* and antisymmetry for *σ _rθ_*. Lastly, note that the parameters *B*_0_ and *D*_1_ do not contribute to the stress as by Equations ( 2.14)–( 2.16), which is straightforwardly verified. They may be considered degeneracies of the solution space and set to zero without loss of generality (cf. [ 7]). The remaining terms in ( 2.25) are


$$\;\phi (r,\theta )=D_{0}r^{2}+C_{1}r^{3}\text{cos(}\theta \text{)}+\sum_{n\geq 2}\left(A_{n}r^{n}+C_{n}r^{n+2}\right)\text{cos(}n\theta \text{),}\;(2.26)$$

which is the form considered for all applications below.

The displacement can be calculated via ( 2.21)–( 2.23) ^2^. We have for the remaining terms in *ϕ*,


$$\;\begin{array}{l} u_{r}(r,\theta )=\frac{1}{2\mu }\{2(1-2\nu )D_{0}r+(1-4\nu )C_{1}r^{2}\cos(\theta )-\frac{1}{2}\text{g}\rho (1-2\nu )r^{2}\cos(\theta )\\ \;+\sum_{n\geq 2}\left[-nA_{n}r^{n-1}+(2-4\nu -n)C_{n}r^{n+1}\right]\text{cos(}n\theta \text{)}\}-\text{Ξ}\;\text{cos(}\theta \text{)}-\nu \kappa r\\ \;+(1+\nu )\alpha (T-T_{0})r,\;(2.27) \end{array}$$

$$\;\begin{array}{l} u_{\theta }(r,\theta )=\frac{1}{2\mu }\{\left(5-4\nu )C_{1}r^{2}\sin(\theta )-\frac{1}{2}\text{g}\rho (1-2\nu )r^{2}\sin(\theta \right)\\ \;+\sum_{n\geq 2}\left[nA_{n}r^{n-1}+(4-4\nu +n)C_{n}r^{n+1}\right]\text{sin(}n\theta \text{)}\}+\text{Ξ}\;\text{sin(}\theta \text{)}+c^{*}r,\;(2.28) \end{array}$$

where *c* ^∗^, Ξ are integration constants. These are fixed by the boundary conditions imposed at the contact point *u _r_* ( *a,* 0) = 0 = *u _θ_* ( *a,* 0), which imply *c* ^∗^ = 0 and


$$\begin{array}{l} \text{Ξ}\;\text{:=}\;\frac{1}{2\mu }\{2(1-2\nu )D_{0}a+(1-4\nu )C_{1}a^{2}-\frac{1}{2}\text{g}\rho (1-2\nu )a^{2}\\ \;+\sum_{n\geq 2}\left[-nA_{n}a^{n-1}+(2-4\nu -n)C_{n}a^{n+1}\right]\}-\nu \kappa a+(1+\nu )\alpha (T-T_{0})a,\;(2.29) \end{array}$$

provided that the sums converge.

## 3 Boundary conditions

We now have general expressions for the stresses ( 2.14)–( 2.16), the strains ( 2.18)–( 2.20), and the displacements ( 2.27)–( 2.28), in terms of the coefficients { *D*_0_, *C*_1_, *A _n_*, *C _n_*} for *n* ≥ 2. These coefficients can be determined from the boundary conditions algebraically.

At the boundary of the cylinder we consider an angle dependent pressure *f*( *θ*) acting in the radial direction and a shear force per unit area *g*( *θ*). The boundary conditions in linear elasticity require the stresses at the boundary to react to the external forces as 


$$\;\sigma_{r\theta }{\text{|}}_{\partial U}=g(\theta ),\;(3.1)$$

$$\;\sigma_{rr}{\text{|}}_{\partial U}=f(\theta ).\;(3.2)$$

Our assumption of mirror-symmetry implies that the boundary conditions can be Fourier decomposed into sine and cosine series respectively,


$$\;\sigma_{r\theta }{\text{|}}_{\partial U}=\sum_{n\geq 0}g_{n}\text{sin(}n\theta \text{),}\;(3.3)$$

$$\;\sigma_{rr}{\text{|}}_{\partial U}=\frac{1}{2}f_{0}+\sum_{n\geq 1}f_{n}\text{cos(}n\theta \text{),}\;(3.4)$$

with the coefficients given explicitly by


$$\;g_{n}=\frac{2}{\pi }\int_{0}^{\pi }\text{d}\theta \;g\text{(}\theta \text{)sin(}n\theta \text{),}\;(3.5)$$

$$\;f_{n}=\frac{2}{\pi }\int_{0}^{\pi }\text{d}\theta \;f\text{(}\theta \text{)cos(}n\theta )\text{.}\;(3.6)$$

On the other hand, we know that the solution can be written using ( 2.14)–( 2.16) with ( 2.26) for the Airy stress function; explicitly 


$$\;\sigma_{r\theta }{\text{|}}_{\partial U}=2C_{1}a\;\text{sin(}\theta \text{)+}\sum_{n\geq 2}\left[(n-1)A_{n}+a^{2}(n+1)C_{n}\right]na^{n-2}\sin(n\theta ),\;(3.7)$$

$$\;\begin{array}{l} \sigma_{rr}{\text{|}}_{\partial U}=2D_{0}+(2C_{1}-\text{g}\rho )a\;\cos\text{(}\theta \text{)}-\sum_{n\geq 2}\left[(n-1)nA_{n}+a^{2}(n^{2}-n-2)C_{n}\right]a^{n-2}\cos(n\theta ).\;(3.8) \end{array}$$

Comparing term by term determines the coefficients. We are generally interested in the case with vanishing shear forces, i.e. *g*( *θ*) = 0, which corresponds to only normal forces or the frictionless limit. Then,


$$\;C_{1}=0\;\text{and}\;C_{n}=\frac{1-n}{a^{2}(1+n)}A_{n},\;(3.9)$$

and for the radial stress


$$\;\sigma_{rr}{\text{|}}_{\partial U}=2D_{0}-a\text{g}\rho \;\cos\text{(}\theta \text{)}\;-2\sum_{n\geq 2}A_{n}(n-1)a^{n-2}\cos(n\theta ).\;(3.10)$$

Comparing coefficients with ( 3.4), we find 


$$\;D_{0}=\frac{1}{4}f_{0},\;(3.11)$$

$$\;A_{n}=-\frac{1}{2(n-1)}a^{2-n}f_{n},\;(3.12)$$

for *n* ≥ 2, with


$$\;f_{1}=-a\text{g}\rho .\;(3.13)$$

We see that the Fourier coefficient *f*_1_ in the function *f* in ( 3.2) is not arbitrary, and is determined by the body force.

The boundary conditions in the *z*-direction are given by the models shown in Figure 2.1. We focus on subfigure (c), with the special case without tension force *F _T_* corresponding to subfigure (b). By definition, the displacement boundary conditions are given by ( 2.5), since we allow for elongation of the open end. The boundary condition for the stress is given by


$$\;F_{T}=\int_{U(z=L)}\sigma_{zz}r\text{d}r\;\text{d}\theta \text{,}\;(3.14)$$

i.e. the forces on the end face of the cylinder have to match the stresses on the end face. Using ( 2.12), this can be rewritten as


$$\;F_{T}=\int_{U(z=L)}\nu (\sigma_{xx}+\sigma_{yy})r\;\text{d}r\;\text{d}\theta +\pi a^{2}E[\kappa -\alpha (T-T_{0})].\;(3.15)$$

The integral can be calculated either explicitly or numerically for the solutions in Section 4, providing thus a relation between the forces acting on the system, *κ*, and the remaining parameters that appear in the problem.

This can be restated as an equation for the elongation *κ* as a function of tension *F _T_* and the plane stresses along the fiber:


$$\;\kappa =-\frac{1}{\pi a^{2}E}\int_{U(z=L)}\nu (\sigma_{xx}+\sigma_{yy})r\;\text{d}r\;\text{d}\theta +\alpha (T-T_{0})+\frac{F_{T}}{\pi a^{2}E}.\;(3.16)$$

For a waveguide of total length *L*, this implies a change in length 


$$L\mapsto L+\kappa L=L\left[1-\frac{1}{\pi a^{2}E}\int_{U(z=L)}\nu (\sigma_{xx}+\sigma_{yy})r\;\text{d}r\;\text{d}\theta +\alpha (T-T_{0})+\frac{F_{T}}{\pi a^{2}E}\right].\;(3.17)$$

## 4 Contact models

Having formally solved the problem for arbitrary boundary conditions in the preceding section, we now implement the boundary conditions for the configurations shown in Figures 1.2a– 1.2d.

As already pointed out in the Introduction, the solutions for the configurations of Figures 1.2a– 1.2b, that we are about to derive, with the boundary conditions corresponding to contact lines, describe unphysical displacement fields, diverging at the contact interfaces. We show that this can be cured by deriving a solution involving extended contact regions, using the Hertz contact deformations formalism. We show that even though the displacements differ between these approaches, the stresses near the center of the waveguide are in reasonable agreement.

### 4.1 Line contacts

***4.1.1 Resting on a contact line*.** The simplest physical model is given by a cylindrical waveguide lying on an infinite plane, contacting as a first approximation only on a line. This is reminiscent of the famous Flamant solution with circular cross-section (see in particular [ 7, Problem 3 of Chapter 12]).

The boundary conditions are given by


$$\;\sigma_{r\theta }{\text{|}}_{\partial U}=0,\;(4.1)$$

$$\;\sigma_{rr}{\text{|}}_{\partial U}=\delta (\theta )-p,\;(4.2)$$

where is the pressure with which the contact line is resisting the weight of the waveguide and the ambient pressure, taken to be constant. To put this into physical terms, we apply neither tangential nor radial forces, except for the contact line with the “contact wire”.

This model is particularly convenient, since the *δ*-distribution has a Fourier series expansion as


$$\;\delta (\theta )=\frac{1}{2\pi }\left[1+2\sum_{1\leq n}\cos(n\theta )\right].\;(4.3)$$

We find for the coefficients in the Michell solution


$$\;D_{0}=\frac{}{4\pi }-\frac{p}{2},\;(4.4)$$

$$\;C_{1}=0,\;(4.5)$$

$$\;A_{n}=-\frac{}{2\pi a^{n-2}(n-1)},\;(4.6)$$

$$\;C_{n}=\frac{}{2\pi a^{n}(n+1)},\;(4.7)$$

with


=−πagρ.(4.8)

This last constraint implements the physicality of the calculations, since in the absence of other forces there can only be an equilibrium configuration if the integrated body force of the waveguide


$$\int (-F_{y})r\;\text{d}r\text{d}\theta =\int \text{g}\rho \;r\;\text{d}r\text{d}\theta =\pi a^{2}\text{g}\rho$$

matches the reactive force exterted by the plane, given by


$$\int \delta (\theta )r\;\text{d}\theta {|}_{\partial U}=-\pi a^{2}\text{g}\rho \text{.}$$

The sum in ( 2.26) converges for coefficients ( 4.4)–( 4.7), yielding


$$\phi (r,\theta )=-\frac{1}{2}r^{2}p+\frac{1}{4}r\text{g}\rho \;\left[r(a+r\;\text{cos}(\theta ))-4a^{2}\arctan\left(\frac{r\;\text{sin}(\theta )}{a-r\;\text{cos}(\theta )}\right)\;\text{sin}(\theta )\right],\;(4.9)$$

and further for the stresses


$$\;\begin{array}{l} \sigma_{rr}=A\left[r(6a^{2}+r^{2})\text{cos}(\theta )-a\left(a^{2}+3r^{2}+2(a^{2}+r^{2})\text{cos}(2\theta )-ar\;\text{cos}(3\theta )\right)\right]-p,\;(4.10) \end{array}$$

$$\sigma_{r\theta }=A\left[4a(a^{2}+r^{2})\text{cos}(\theta )-r\left(5a^{2}+r^{2}+2a^{2}\text{cos}(2\theta )\right)\right]\text{sin}(\theta ),\;(4.11)$$

$$\;\begin{array}{l} \sigma_{\theta \theta }=A\left[-r(2a^{2}+r^{2})\text{cos}(\theta )+a\left(r^{2}-a^{2}+2(a^{2}+r^{2})\text{cos}(2\theta )-ar\;\text{cos}(3\theta )\right)\right]-p,\;(4.12) \end{array}$$

with


$$A\;\text{:=}\;\frac{\text{g}\rho (a^{2}-r^{2})}{2{\left[a^{2}+r^{2}-2ar\;\text{cos}(\theta )\right]}^{2}}.$$

**Figure 4.1.**
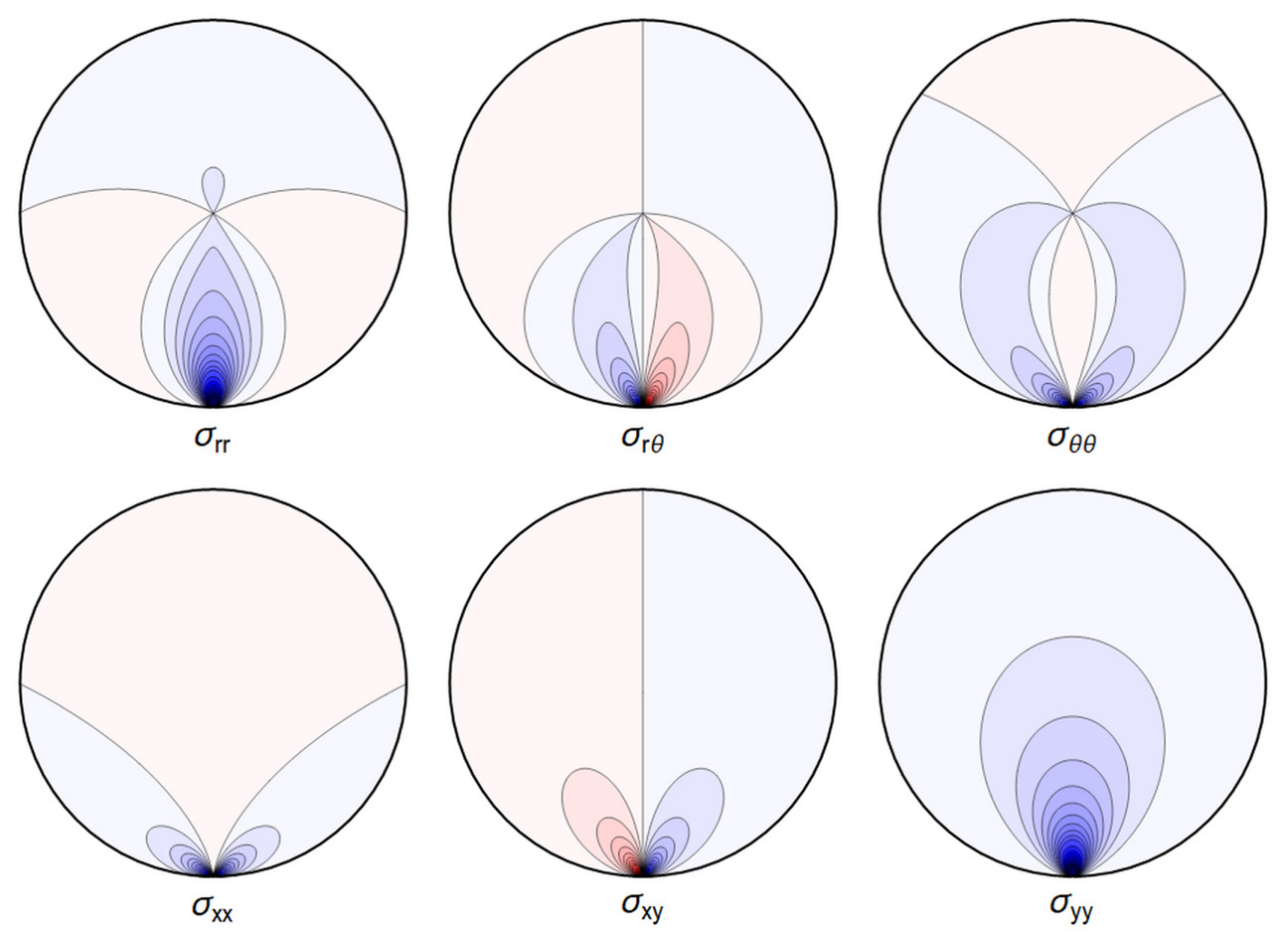
Typical plot of internal stresses for the case of a waveguide resting on an infinitely thin line, with unrealistic parameters arbitrarily chosen for illustration purposes listed in Table 4.1. Darker colors encode larger stresses, with blue color indicating compressive (negative) stresses.

A representative plot of the stresses can be seen in Figure 4.1.

**Table 4.1.** Set of parameters chosen in our visualizations. For conciseness, we express lengths in multiples of *a*, and pressure in multiples of the shear modulus *μ*, leading to dimensionless quantities for the remaining parameters used in numerical calculations.

*ρ*g	*ν*	, *κ, α*	$\tilde{P}$	$\hat{P}$ = $\overset{\vee }{P}$
0.01	0.17	0	1	0.3

**Figure 4.2.**
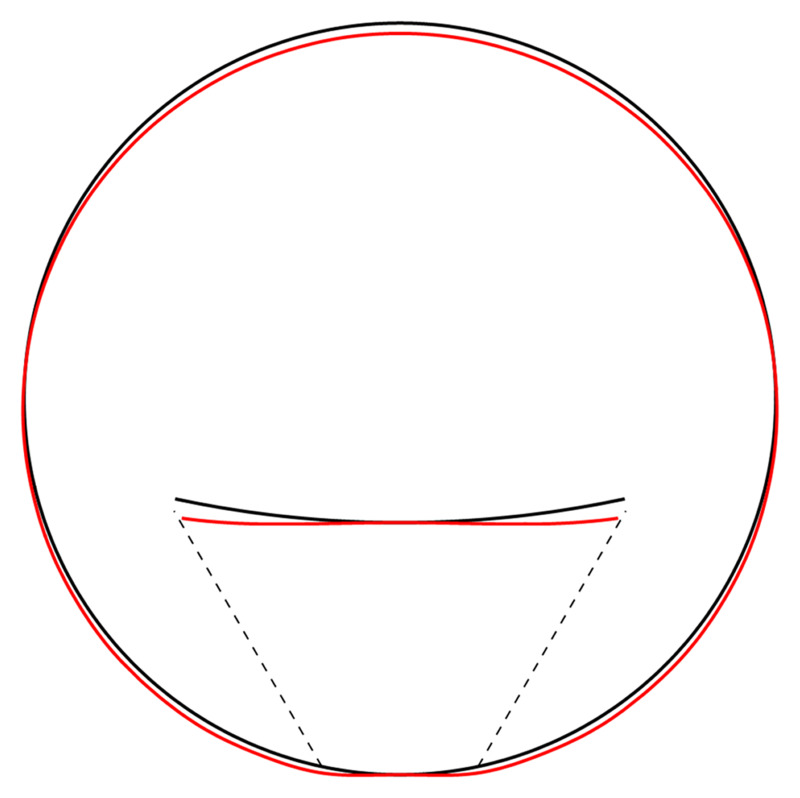
Illustrative deformation for the case of a waveguide resting on an infinitely thin line, after truncating the sums in ( 2.27)–( 2.29) to *n* = 20, drawn in red. The undeformed reference is drawn in black. Here and in similar figures below, the inset shows a magnified section around the contact point.

Equations ( 2.27)–( 2.29) do not make sense, as the sum in Ξ does not converge. But one can find explicit expressions for *u _r_* and *u _θ_* by integration; the resulting formulae are lengthy and not very enlightening, therefore we did not include them here. Not unexpectedly, and similar to the Flamant solution [ 2, Chapter 8.4.7] , the singularity in the function *A* at *r* = *a* and cos *θ* = 1 leads to an infinite displacement there. Hence the boundary condition *u*( *r* = *a, θ* = 0) = 0 cannot be imposed. However, the displacements obtained by direct integration are finite away from the contact point, in particular near the center of the waveguide (cf. [ 9]). One can also truncate the series ( 2.27)–( 2.28) which leads to finite solutions, illustrated in Figure 4.2.

***4.1.2 Squeezed between two lines*.** A second straightforward case is given by the waveguide being squeezed between two lines, with an arbitrary pressure $\tilde{P}$ pushing from the top. See also [ 2, Example 8–10] and [ 7, Problem 1 of Chapter 12], where a similar problem is modelled by superposition of three particular stress fields, including two Flamant solutions together with a uniform radial tension loading. The boundary conditions are given by


$$\;\sigma_{r\theta }{\text{|}}_{\partial U}=0,\;(4.13)$$

$$\;\sigma_{rr}{\text{|}}_{\partial U}=\delta (\theta )+\tilde{P}\delta (\theta -\pi )-p.\;(4.14)$$

The calculation is completely analogous to the above, with the boundary condition enforcing


$$\;D_{0}=\frac{+\tilde{P}}{4\pi }-\frac{p}{2},\;(4.15)$$

$$\;C_{1}=0,\;(4.16)$$

$$\;A_{n}=-\frac{+{\left(\text{−}1\right)}^{n}\tilde{P}}{2\pi a^{n-2}(n-1)},\;(4.17)$$

$$\;C_{n}=\frac{+{\left(\text{−}1\right)}^{n}\tilde{P}}{2\pi a^{n}(n+1)},\;(4.18)$$

with


$$\;=\tilde{P}-\pi a\text{g}\rho .\;(4.19)$$

Again, the physical interpretation is that now the force from above necessitates a reactive force from below larger than in ( 4.8) to achieve equilibrium.

**Figure 4.3.**
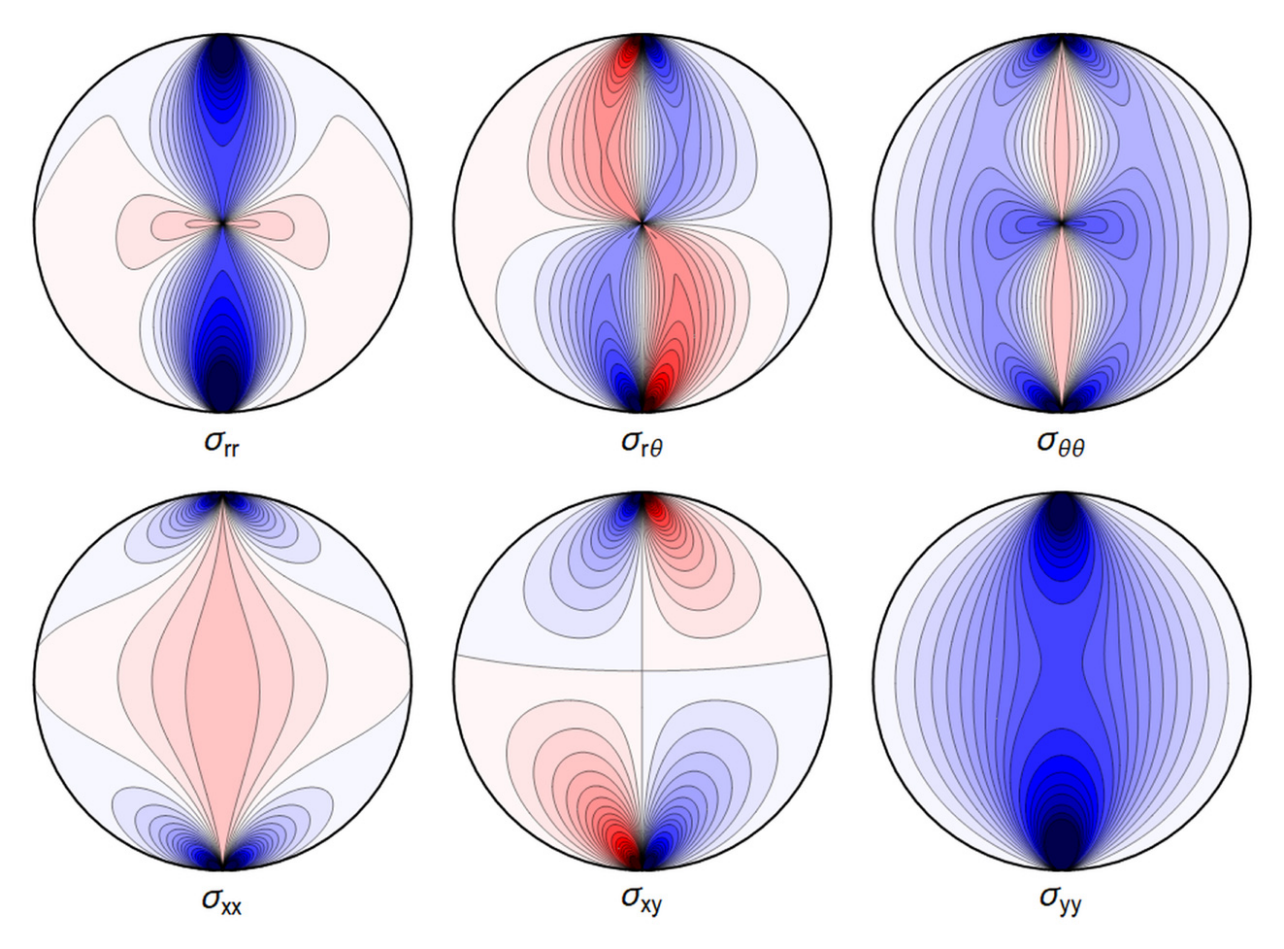
Typical plot of internal stresses for the case of a waveguide squeezed between two lines; same parameters and color coding as in Figure 4.1.

Denoting by $\sigma_{ij}^{I}$ the right-hand sides of ( 4.10)–( 4.12), the stress components read


$$\;\sigma_{rr}=\sigma_{rr}^{I}-B(a^{2}-r^{2})\left[a^{4}-2a^{2}r^{2}-r^{4}+2a^{4}\;\text{cos}(2\theta )\right],\;(4.20)$$

$$\;\sigma_{r\theta }=\sigma_{r\theta }^{I}+2Ba^{2}(a^{4}-r^{4})\text{sin}(2\theta ),\;(4.21)$$

$$\;\sigma_{\theta \theta }=\sigma_{\theta \theta }^{I}-B\left[a^{6}+5a^{4}r^{2}+a^{2}r^{4}+r^{6}-2a^{2}(a^{4}+a^{2}r^{2}+2r^{4})\;\text{cos}(2\theta )\right],\;(4.22)$$

with $B\;\text{:=}\;\frac{\tilde{P}}{\pi }(a^{2}-r^{2}){\left[a^{4}+r^{4}-2a^{2}r^{2}\;\text{cos}(2\theta )\right]}^{-2}$ . Again, visualizing the result gives a clear picture. See Figure 4.3.

**Figure 4.4.**
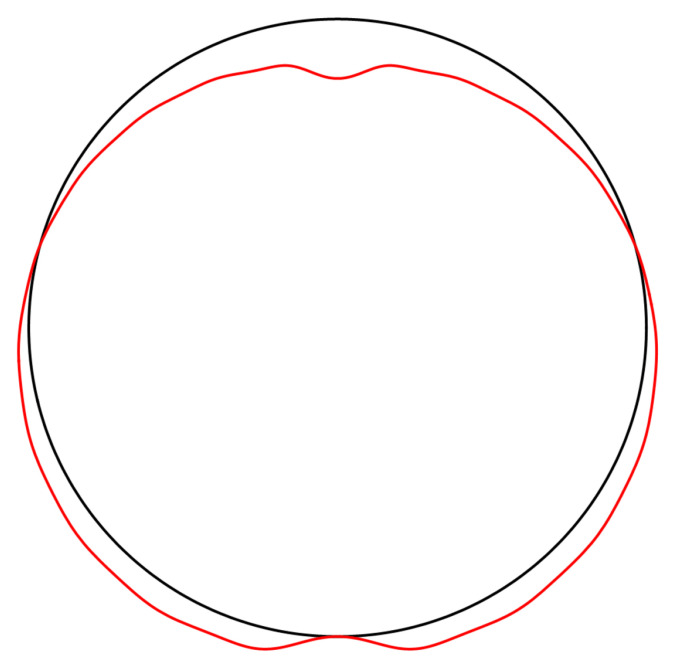
Illustrative deformation for the case of a waveguide squeezed between two lines, after truncating the sums in ( 2.27)-( 2.29) to *n* = 20, drawn in red. The undeformed reference is drawn in black.

As before, the deformation diverges at the “contact wires”. A truncated sum is shown in Figure 4.4.

### 4.2 Hertz contact deformations

The proper solution for the contact problem in linear elasticity is given by the Hertz contact deformation [ 10], with a modern derivation given by [ 3, Chapter 9]. In the special case of two cylinders contacting length-wise the two-dimensional contact region extends to a contact strip, which can be described as a contact line in terms of the cross-section (cf. [ 3, Problem 2 of Chapter 9]). The total force per unit length pushing two bodies into each other is distributed over a region { *x* : *−s ≤ x ≤ s*}, where


$$\;s=\sqrt{\frac{4F}{\pi }\;\left(\frac{1-\nu^{2}}{E}+\frac{1-{\nu^{\prime }}^{2}}{E^{\prime }}\right)\;\frac{RR^{\prime }}{R+R^{\prime }}},\;(4.23)$$

with *{ν, E, R}* the material properties and radius of curvature of the body in question and { *ν′, E′, R′}* for the body in contact. The pressure distribution over the contact region, which we denote by *P _y_*, is taken to be


$$\;P_{y}=\frac{2F}{\pi s}\sqrt{1-\frac{x^{2}}{s^{2}}}.\;(4.24)$$

This can be restated in the form of boundary conditions


$$\;\sigma_{r\theta }{\text{|}}_{\partial U}=0,\;(4.25)$$

$$\;\sigma_{rr}{\text{|}}_{\partial U}=\{\begin{array}{ll} \frac{2F}{\pi a\;\text{tan}(\text{Θ})}\sqrt{1-\frac{{\text{tan}}^{2}(\theta )}{{\text{tan}}^{2}(\text{Θ})}} & -\text{Θ}\leq \theta \leq \text{Θ}\\ 0 & \text{otherwise},\;(4.26) \end{array}$$

where


$$\;\text{Θ}=\text{arctan}\left(\frac{s}{a}\right).\;(4.27)$$

For the case of an infinite cylinder resting on a rigid plane, we simply take *R′, E′ → ∞* and *ν′ →* 0.

**Figure 4.5.**
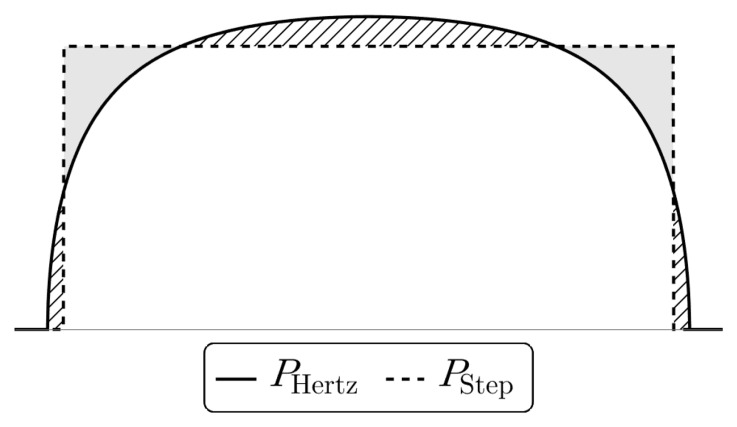
Comparison of the pressure distribution in the contact region for the exact Hertz solution ( 4.26) and an approximation using constant pressure in the contact region. The shaded and dashed regions cause overshoot and undershoot in the displacement figures below.

Finding the cosine expansion of ( 4.26) is not obvious. Instead, one can approximate the Hertz profile with a step-function as in Figure 4.5, with straightforward cosine expansion, allowing us to find exact expressions for the Airy functions and stresses in the settings discussed below. The half-width of the step function can be determined, based on the Hertz solution, to


$$\;s_{\text{Hertz}}=\sqrt{\frac{4aF}{\pi }\frac{1-\nu^{2}}{E}},\;(4.28)$$

$$\;{\text{Θ}}_{\text{Hertz}}=\text{arctan}\left(\frac{s_{\text{Hertz}}}{a}\right),\;(4.29)$$

via ( 4.23) and ( 4.27), ensuring a physically motivated boundary pressure distribution in response to external forces.

This approach yields exact solutions closely matching the full Hertz problem, avoiding the undesirable behaviour at the contact point exhibited by the solutions above. We expect that this approach is sufficient for weak forces and away from the contact region.

***4.2.1 Resting on a rigid plane*.** Approximating the Hertz contact solution of Section 4.2 by a step-function of constant pressure *P* for a single contact region from below leads to the boundary conditions


$$\;\sigma_{r\theta }{\text{|}}_{\partial U}=0,\;(4.30)$$

$$\;\sigma_{rr}{|}_{\partial U}=P_{\chi \left[-\Theta ,\Theta \right]}(\theta )-p(1-\chi_{\left[-\Theta ,\Theta \right]}(\theta ))=:P^{\prime }\chi_{\left[-\Theta ,\Theta \right]}(\theta )-p,\;(4.31)$$

where


$$\;\chi_{A}(x):=\{\begin{array}{ll} 1 & x\in A\\ 0 & \text{else},\;(4.32) \end{array}$$

is the indicator function and Θ *≈* Θ _Hertz_.

A Fourier cosine series for the boundary-values of the Airy function leads to the following coefficients in ( 2.26):


$$\;D_{0}=\frac{P^{\prime }\text{Θ}}{2\pi }-\frac{p}{2},\;(4.33)$$

$$\;C_{1}=0,\;(4.34)$$

$$\;A_{n}=-\frac{P^{\prime }\text{sin}(n\text{Θ})}{\pi a^{n-2}n(n-1)},\;(4.35)$$

$$\;C_{n}=\frac{P^{\prime }\text{sin}(n\text{Θ})}{\pi a^{n}n(n+1)},\;(4.36)$$

with


$$\;P^{\prime }=\frac{ag\rho \pi }{2\text{sin}(\text{Θ})}.\;(4.37)$$

The summed expression for the Airy function is


$$\begin{array}{l} \phi_{\text{single}}=\frac{1}{4}g\rho \left[\left(2a^{2}+r^{2})r\;\text{cos}(\theta )-ar^{2}\frac{\text{Θ}}{\text{sin}(\text{Θ})}+\frac{a}{\text{sin}(\text{Θ})}(\zeta (\theta -\text{Θ})-\zeta (\theta +\text{Θ})\right)\right]-\frac{1}{2}r^{2}p,\;(4.38) \end{array}$$

where the subscript “single” refers to a single contact region, with


$$\;\zeta (x)\;:=\left[a^{2}+r^{2}-2ar\;\text{cos}(x)\right]\;\text{arctan}\;\left(\frac{r\;\text{sin}(x)}{a-r\;\text{cos}(x)}\right).\;(4.39)$$

**Figure 4.6.**
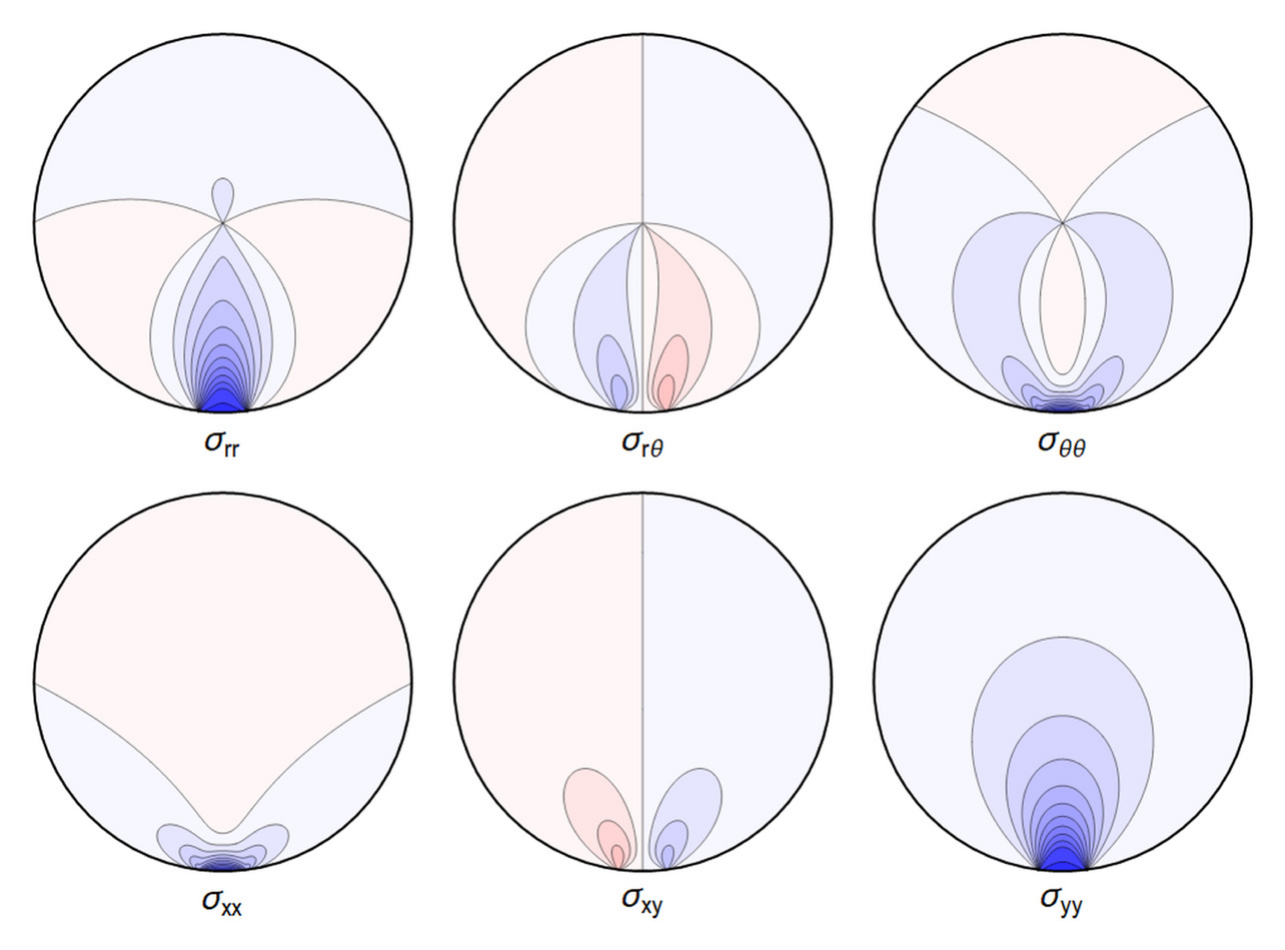
Typical internal stresses for a waveguide resting on an extended contact zone. Here, and in the following figures, we use the same parameters and the same color coding as in Figure 4.1.

**Figure 4.7.**
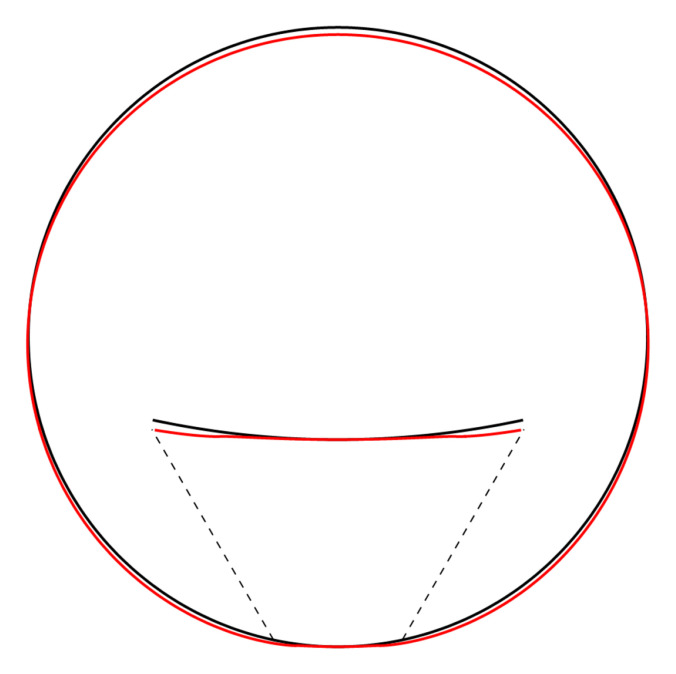
Deformation for the case of a waveguide resting on a rigid plane, drawn in red. The undeformed reference is drawn in black. Here, and in similar figures that follow, the (barely visible) indentation is an artefact of the approximation illustrated in Figure 4.5.

All the sums converge, leading to an admissible displacement field. The stresses and the displacements are shown in Figure 4.6 and Figure 4.7.

***4.2.2 Squeezed between two rigid planes*.** The next simplest description of a spooled waveguide is one with extended contact regions both above and below. We model this by squeezing the fiber between two rigid planes in the spirit of Section 4.2.1, i.e. boundary conditions


$$\sigma_{r\theta }{|}_{\partial U}=0,\;(4.40)$$

$$\begin{array}{l} \sigma_{rr}{|}_{\partial U}=P_{\chi \left[-\Theta ,\Theta \right]}(\theta )+\tilde{P}\chi_{\left[\pi -\tilde{\Theta },\pi +\tilde{\Theta }\right]}(\theta )-p\left[1-\chi_{\left[-\Theta ,\Theta \right]}(\theta )-\chi_{\left[\pi -\tilde{\Theta },\pi +\tilde{\Theta }\right]}(\theta )\right]\\ \;=:\;P^{\prime }\chi_{\left[-\Theta ,\Theta \right]}(\theta )+{\tilde{P}}^{\prime }\chi_{\left[\pi -\tilde{\Theta },\pi +\tilde{\Theta }\right]}(\theta )-p,\;(4.41) \end{array}$$

with Θ now an angle which approximates half of the contact region from below and $\tilde{\Theta }$ the same from above.

The Fourier coefficients for the Airy function are found to be


$$\;D_{0}=\frac{P^{\prime }\text{Θ}+{\tilde{P}}^{\prime }\tilde{\text{Θ}}}{2\pi }-\frac{p}{2},\;(4.42)$$

$$\;C_{1}=0,\;(4.43)$$

$$\;A_{n}=-\frac{P^{\prime }\;\text{sin}(n\text{Θ})+{\left(-1\right)}^{n}{\tilde{P}}^{\prime }\;\text{sin}(n\tilde{\text{Θ}})}{\pi a^{n-2}n(n-1)},\;(4.44)$$

$$\;C_{n}=\frac{P^{\prime }\text{sin}(n\text{Θ})+{\left(-1\right)}^{n}{\tilde{P}}^{\prime }\;\text{sin}(n\tilde{\text{Θ}})}{\pi a^{n}n(n+1)},\;(4.45)$$

with


$$\;2\;\text{sin}(\text{Θ})P^{\prime }=2{\tilde{P}}^{\prime }\;\text{sin}(\tilde{\text{Θ}})-ag\rho \pi .\;(4.46)$$

The re-summed expression for the Airy function is


$$\begin{array}{l} \phi_{\text{double}}=\phi_{\text{single}}+\frac{{\tilde{P}}^{\prime }}{2\pi }[r^{2}\;\left(\tilde{\text{Θ}}+\text{Θ}\frac{\text{sin}(\tilde{\text{Θ}})}{\text{sin}(\text{Θ})}\right)+\xi (\theta -\tilde{\text{Θ}})-\xi (\theta +\tilde{\text{Θ}})-\frac{\text{sin}(\tilde{\text{Θ}})}{\text{sin}(\text{Θ})}\left(\zeta (\theta -\text{Θ})-\zeta (\theta +\text{Θ})\right)],\;(4.47) \end{array}$$

where


$$\;\xi (x)\;:=\left[a^{2}+r^{2}+2ar\;\text{cos}(x)\right]\;\text{arctan}\;\left(\frac{r\;\text{sin}(x)}{a+r\;\text{cos}(x)}\right).\;(4.48)$$

**Figure 4.8.**
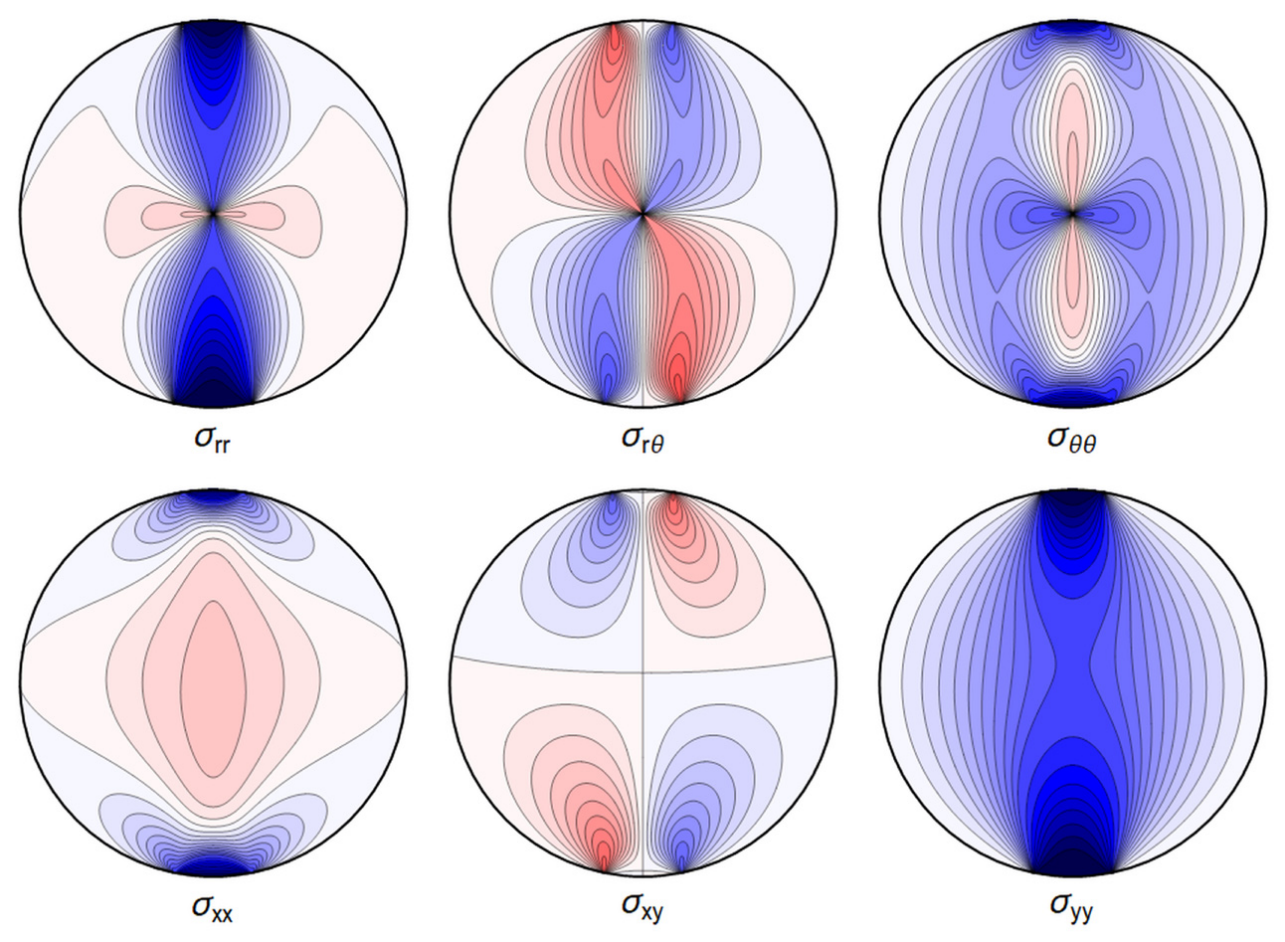
Internal stresses for the case of a waveguide squeezed between two rigid planes with extended contact region.

**Figure 4.9.**
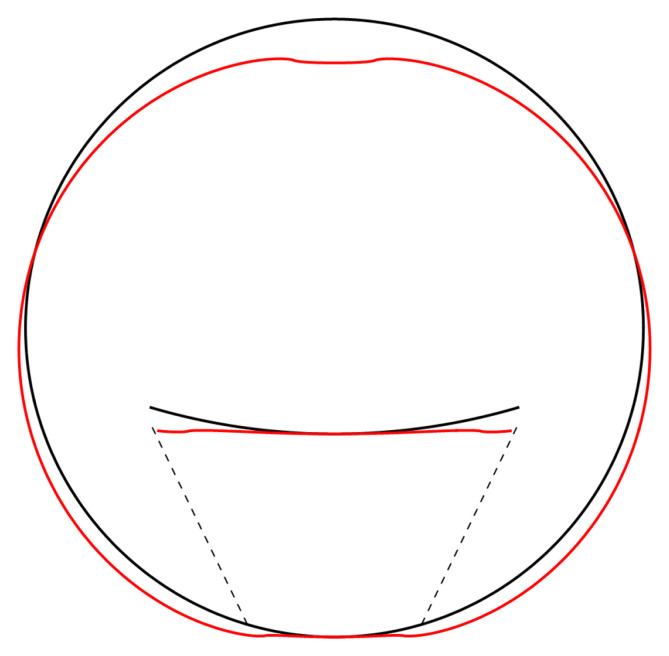
Deformation for the case of a waveguide squeezed between rigid planes of finite width, drawn in red. The undeformed reference is drawn in black.

All the sums converge, leading to a finite displacement field. The stresses and the displacements can be seen in Figure 4.8 and Figure 4.9 respectively. Here, and in the plots that follow, the parameters are related to those of the two contact-lines case via the correspondence


$$\;{\tilde{P}}_{\text{contact}\;\text{line}}={\tilde{P}}_{\text{extended}\;\text{contact}\;\text{region}}\times 2\tilde{\text{Θ}},\;(4.49)$$

**Figure 4.10.**
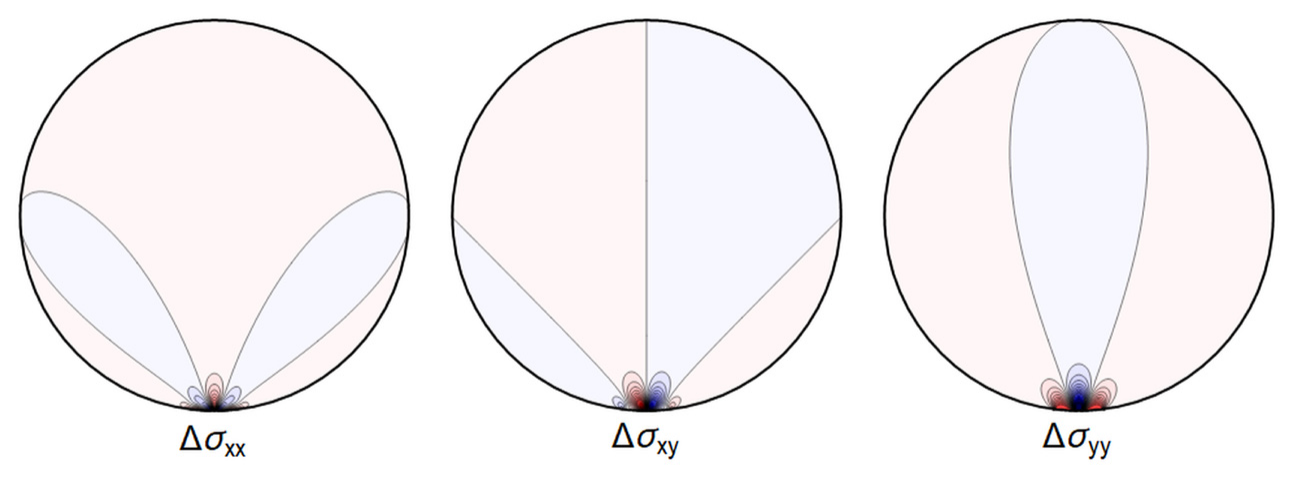
The difference ∆ _*σ*_ between the line contact Figure 4.1 and extended contact region Figure 4.6.

**Figure 4.11.**
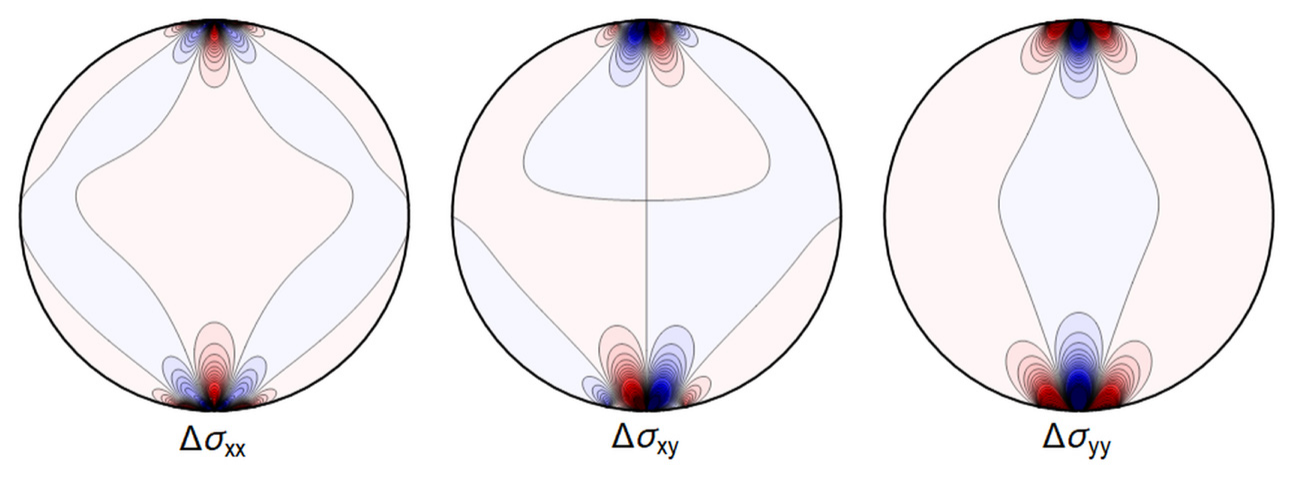
Difference ∆ *σ* between the line contact of Figure 4.3 and the extended contact region of Figure 4.8.

*P*contact line = *P*extended contact region *×* 2Θ *, *(4.49)

which guarantees an identical integrated reaction force from the support. It is of interest to compare the line-contact cases to the corresponding extended region cases, focusing on the central region containing the guiding core of the waveguide. In Figure 4.10 and Figure 4.11 we show the differences for the same set of parameters as used in the plots. We can quantify the differences away from the contact points by comparing the maximal values over the regions


$$U_{1/2}=\{(r,\theta )\;:0<r\leq \frac{a}{2},-\pi <\theta \leq \pi \}$$

and


$$U_{1/10}=\{(r,\theta )\;:0<r\leq \frac{a}{10},-\pi <\theta \leq \pi \}\text{,}$$

and setting


$$\;{\text{Δ}}_{U}(\sigma_{1},\sigma_{2})=\frac{2\;{\text{max}}_{U}|\sigma_{1}-\sigma_{2}|}{{\text{max}}_{U}|\sigma_{1}|+{\text{max}}_{U}|\sigma_{2}|}.\;(4.50)$$

The results, for the parameters of Table 4.1 (used in all our figures), are shown in Table 4.2 and Table 4.3.

**Table 4.2.** Relative differences for the single contact region models.

	*σ _rr_*	*σ _rθ_*	*σ _θθ_*	*σ _xx_*	*σ _xy_*	*σ _yy_*
∆ _*U*_1/2__	2 *.*9%	4 *.*4%	5 *.*5%	11 *.*7%	6 *.*5%	2 *.*9%
∆ _*U*_1/10__	0 *.*6%	1 *.*1%	0 *.*9%	2 *.*5%	2 *.*3%	0 *.*6%

A reasonable approximation is obtained in the central region for the values of parameters considered if a 11% error is less than the measurement errors at hand.

**Table 4.3.** Relative differences for the double-contact-region models.

	*σ _rr_*	*σ _rθ_*	*σ _θθ_*	*σ _xx_*	*σ _xy_*	*σ _yy_*
∆ _*U*_1/2__	2.4%	9.9%	7.7%	11.0%	17.2%	3.9%
∆ _*U*_1/10__	1.4%	2.0%	1.7%	5.1%	10.4%	1.5%

***4.2.3 Four contact regions*.** We consider now a configuration as in Figure 1.2c, with four contact regions for the waveguide. Using step-function boundary-stresses for the contact region gives


$$\sigma_{r\theta }{|}_{\partial U}=0,\;(4.51)$$

$$\begin{array}{l} \sigma_{rr}{|}_{\partial U}=P_{\chi \left[-\text{Θ},\text{Θ}\right]}(\theta )+\tilde{P}\chi_{\left[\pi -\tilde{\text{Θ}},\pi +\tilde{\text{Θ}}\right]}(\theta )+\bar{P}\left[\chi_{\left[\frac{\pi }{2}-\hat{\text{Θ}},\frac{\pi }{2}+\hat{\text{Θ}}\right]}(\theta )+\chi_{\left[\frac{3\pi }{2}-\hat{\text{Θ}},\frac{3\pi }{2}+\hat{\text{Θ}}\right]}(\theta )\right]\\ \;-p\left[1-\chi_{\left[-\text{Θ},\text{Θ}\right]}(\theta )-\chi_{\left[\pi -\tilde{\text{Θ}},\pi +\tilde{\text{Θ}}\right]}(\theta )-\chi_{\left[\frac{\pi }{2}-\hat{\text{Θ}},\frac{\pi }{2}+\hat{\text{Θ}}\right]}(\theta )-\chi_{\left[\frac{3\pi }{2}-\hat{\text{Θ}},\frac{3\pi }{2}+\hat{\text{Θ}}\right]}(\theta )\right]\\ \;=:P^{\prime }\chi_{\left[-\text{Θ},\text{Θ}\right]}(\theta )+{\tilde{P}}^{\prime }\chi_{\left[\pi -\tilde{\text{Θ}},\pi +\tilde{\text{Θ}}\right]}(\theta )+{\bar{P}}^{\prime }\left[\chi_{\left[\frac{\pi }{2}-\hat{\text{Θ}},\frac{\pi }{2}+\hat{\text{Θ}}\right]}(\theta )+\chi_{\left[\frac{3\pi }{2}-\hat{\text{Θ}},\frac{3\pi }{2}+\hat{\text{Θ}}\right]}(\theta )\right]-p,\;(4.52) \end{array}$$

with Θ now an angle which approximates half of the contact region from below and $\tilde{\Theta }$ the same from above and $\hat{\text{Θ}}$ from the sides.

The resulting Fourier coefficients in the Airy function are 


$$\;D_{0}=\frac{P^{\prime }\text{Θ}+{\tilde{P}}^{\prime }\tilde{\text{Θ}}+2{\tilde{P}}^{\prime }\hat{\text{Θ}}}{2\pi }-\frac{p}{2},\;(4.53)$$

$$\;C_{1}=0,\;(4.54)$$

$$\;A_{n}=-\frac{P^{\prime }\;\text{sin}(n\text{Θ})+{\left(-1\right)}^{n}{\tilde{P}}^{\prime }\;\text{sin}(n\tilde{\text{Θ}})+2{\bar{P}}^{\prime }\;\text{cos}\left(\frac{n\pi }{2}\right)\;\text{sin}(n\hat{\text{Θ}})}{\pi a^{n-2}n(n-1)},\;(4.55)$$

$$\;C_{n}=\frac{P^{\prime }\;\text{sin}(n\text{Θ})+{\left(-1\right)}^{n}{\tilde{P}}^{\prime }\;\text{sin}(n\tilde{\text{Θ}})+2{\bar{P}}^{\prime }\;\text{cos}\left(\frac{n\pi }{2}\right)\;\text{sin}(n\hat{\text{Θ}})}{\pi a^{n}n(n+1)},\;(4.56)$$

with 


$$\;P^{\prime }={\tilde{P}}^{\prime }\frac{\text{sin}(\tilde{\text{Θ}})}{\text{sin}(\text{Θ})}-\frac{ag\rho \pi }{2\;\text{sin}(\text{Θ})}.\;(4.57)$$

**Figure 4.12.**
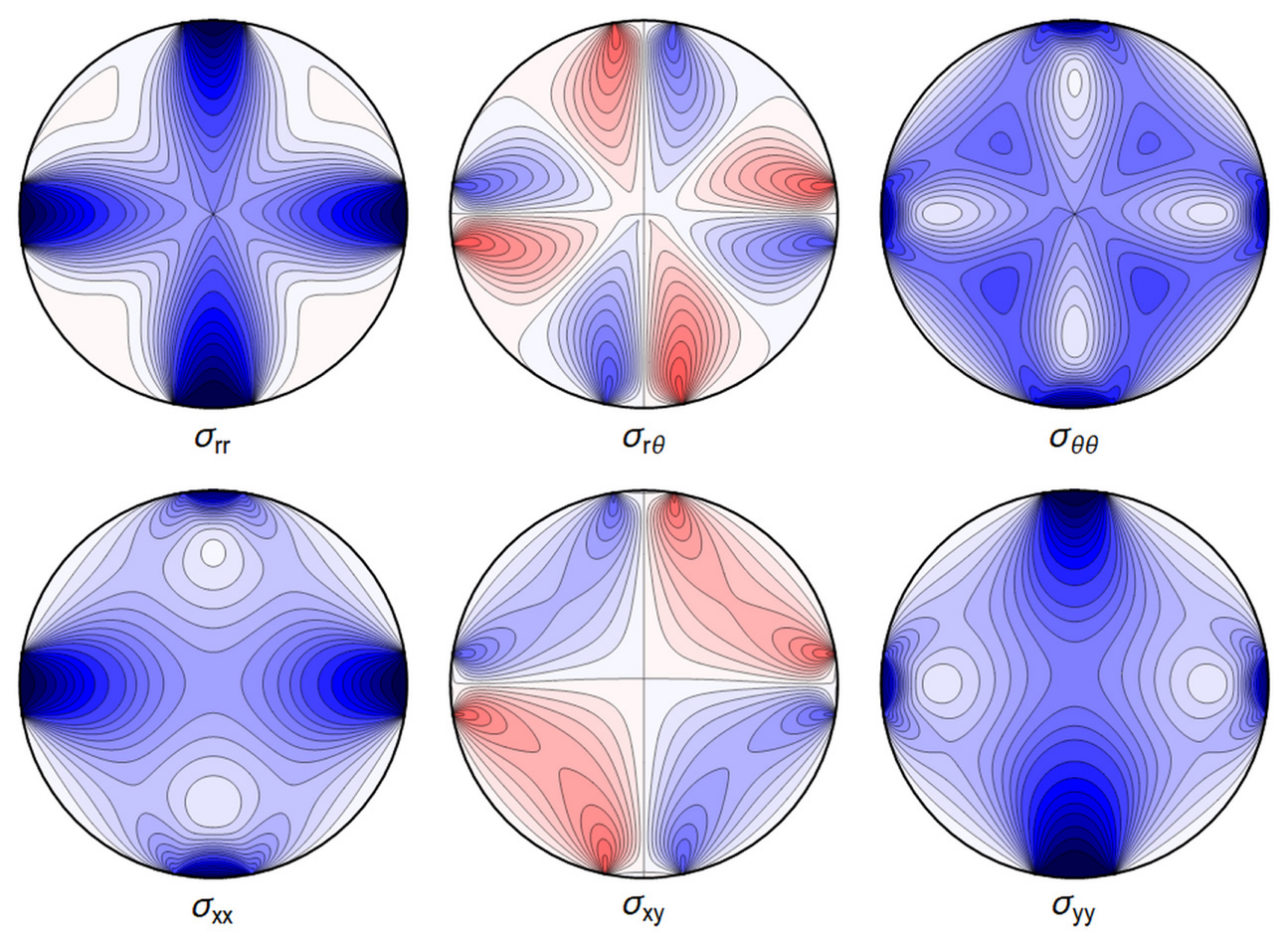
Internal stresses for the case of a waveguide squeezed between four rigid planes from below, above and the sides as depicted in Figure 1.2c, with extended contact region.

**Figure 4.13.**
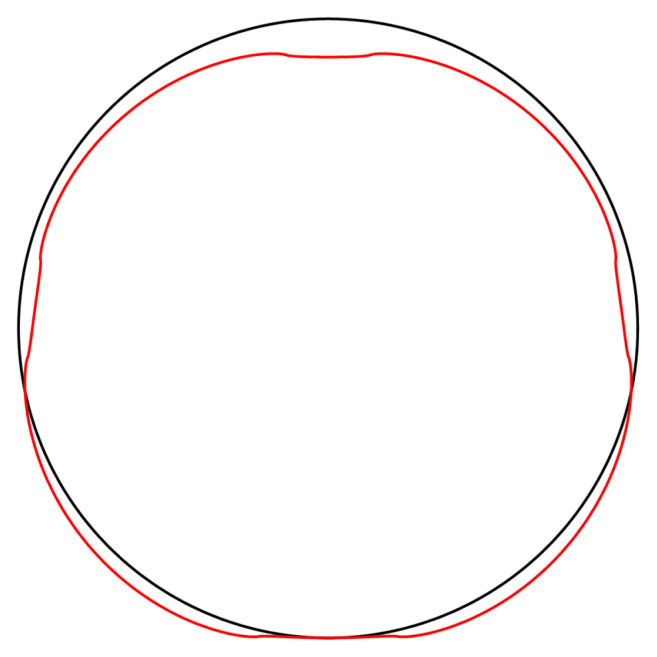
Deformation for the case of a waveguide squeezed between rigid planes of finite width as in Figure 1.2c, drawn in red. The undeformed reference is drawn in black.

All the sums converge, leading to an admissible displacement field. The stresses and the displacement can again be illustrated graphically – see Figure 4.12 and Figure 4.13.

***4.2.4 Squeezed between six rigid planes*.** Our final model for spooled waveguides is given by Figure 1.2d, which we model by imposing the boundary conditions


$$\;\sigma_{r\theta }{|}_{\partial U}=0,\;(4.58)$$

$$\;\begin{array}{l} \sigma_{rr}{|}_{\partial U}=P_{\chi \left[-\text{Θ},\text{Θ}\right]}(\theta )+\tilde{P}\chi_{\left[\pi -\tilde{\text{Θ}},\pi +\tilde{\text{Θ}}\right]}(\theta )+\hat{P}\left[\chi_{\left[\frac{\pi }{3}-\hat{\text{Θ}},\frac{\pi }{3}+\hat{\text{Θ}}\right]}(\theta )+\chi_{\left[\frac{5\pi }{3}-\hat{\text{Θ}},\frac{5\pi }{3}+\hat{\text{Θ}}\right]}(\theta )\right]\\ \;+\overset{\vee }{P}\left[\chi_{\left[\frac{2\pi }{3}-\overset{\vee }{\text{Θ}},\frac{2\pi }{3}+\overset{\vee }{\text{Θ}}\right]}(\theta )+\chi_{\left[\frac{4\pi }{3}-\overset{\vee }{\text{Θ}},\frac{4\pi }{3}+\overset{\vee }{\text{Θ}}\right]}(\theta )\right]\\ \;-p[1-\chi_{\left[-\text{Θ},\text{Θ}\right]}(\theta )-\chi_{\left[\pi -\tilde{\text{Θ}},\pi +\tilde{\text{Θ}}\right]}(\theta )-\chi_{\left[\frac{\pi }{3}-\hat{\text{Θ}},\frac{\pi }{3}+\hat{\text{Θ}}\right]}(\theta )-\chi_{\left[\frac{5\pi }{3}-\hat{\text{Θ}},\frac{5\pi }{3}+\hat{\text{Θ}}\right]}(\theta )\\ \;-\chi_{\left[\frac{2\pi }{3}-\overset{\vee }{\text{Θ}},\frac{2\pi }{3}+\overset{\vee }{\text{Θ}}\right]}(\theta )+\chi_{\left[\frac{4\pi }{3}-\overset{\vee }{\text{Θ}},\frac{4\pi }{3}+\overset{\vee }{\text{Θ}}\right]}(\theta )]\\ \;=P^{\prime }\chi_{\left[-\text{Θ},\text{Θ}\right]}(\theta )+\tilde{P}\prime \chi_{\left[\pi -\tilde{\text{Θ}},\pi +\tilde{\text{Θ}}\right]}(\theta )+\hat{P}\prime \left[\chi_{\left[\frac{\pi }{3}-\hat{\text{Θ}},\frac{\pi }{3}+\hat{\text{Θ}}\right]}(\theta )+\chi_{\left[\frac{5\pi }{3}-\hat{\text{Θ}},\frac{5\pi }{3}+\hat{\text{Θ}}\right]}(\theta )\right]\\ \;+\overset{\vee }{P^{\prime }}\left[\chi_{\left[\frac{2\pi }{3}-\overset{\vee }{\text{Θ}},\frac{2\pi }{3}+\overset{\vee }{\text{Θ}}\right]}(\theta )+\chi_{\left[\frac{4\pi }{3}-\overset{\vee }{\text{Θ}},\frac{4\pi }{3}+\overset{\vee }{\text{Θ}}\right]}(\theta )\right]-p,\;(4.59) \end{array}$$

with Θ now an angle which approximates half of the contact region from below, and $\tilde{\Theta }$ the same from above, and $\hat{\text{Θ}}$ and $\overset{\vee }{\text{Θ}}$ from the sides.

The associated Fourier cosine series for the Airy functions has coefficients


$$D_{0}=\frac{P^{\prime }\text{Θ}+{\tilde{P}}^{\prime }\tilde{\text{Θ}}+2{\hat{P}}^{\prime }\hat{\text{Θ}}+2\overset{\vee }{P^{\prime }}\overset{\vee }{\text{Θ}}}{2\pi }-\frac{p}{2},\;(4.60)$$

$$C_{1}=0,\;(4.61)$$

$$\;A_{n}=-\frac{1}{\pi a^{n-2}n(n-1)}\left[P^{\prime }\;\text{sin}(n\text{Θ})+{\left(\text{−}1\right)}^{n}{\tilde{P}}^{\prime }\;\text{sin}(n\tilde{\text{Θ}})+2{\hat{P}}^{\prime }\;\text{cos}\left(\frac{n\pi }{3}\right)\;\text{sin}(n\hat{\text{Θ}})+\overset{\vee }{2P^{\prime }}\text{cos}\left(\frac{2n\pi }{3}\right)\;\text{sin}(n\overset{\vee }{\text{Θ}})\right],\;(4.62)$$

$$\;C_{n}=\frac{1}{\pi a^{n}n(n+1)}\left[P^{\prime }\;\text{sin}(n\text{Θ})+{\left(\text{−}1\right)}^{n}{\tilde{P}}^{\prime }\;\text{sin}(n\tilde{\text{Θ}})+2{\hat{P}}^{\prime }\;\text{cos}\left(\frac{n\pi }{3}\right)\;\text{sin}(n\hat{\text{Θ}})+\overset{\vee }{2P^{\prime }}\text{cos}\left(\frac{2n\pi }{3}\right)\;\text{sin}(n\overset{\vee }{\text{Θ}}\right)],\;(4.63)$$

with


$$\;P^{\prime }={\tilde{P}}^{\prime }\;\frac{\text{sin}(\tilde{\text{Θ}})}{\text{sin}(\text{Θ})}-{\hat{P}}^{\prime }\;\frac{\text{sin}(\hat{\text{Θ}})}{\text{sin}(\text{Θ})}+\overset{\vee }{P^{\prime }}\frac{\text{sin}(\overset{\vee }{\text{Θ}})}{\text{sin}(\text{Θ})}-\frac{ag\rho \pi }{2\;\text{sin}(\text{Θ})}.\;(4.64)$$

**Figure 4.14.**
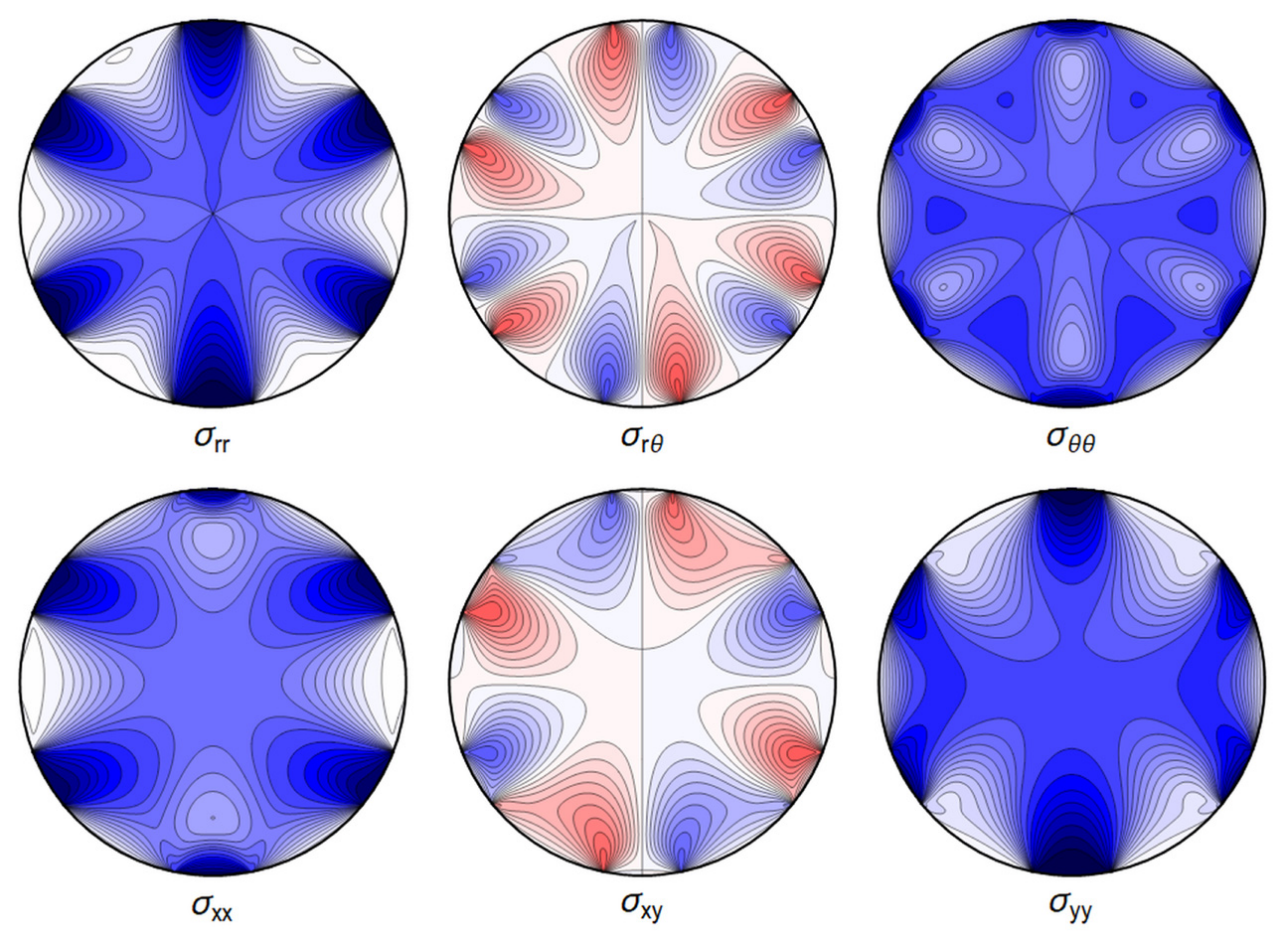
Internal stresses for the case of a waveguide squeezed between six rigid planes with extended contact regions, as in Figure 1.2d.

**Figure 4.15.**
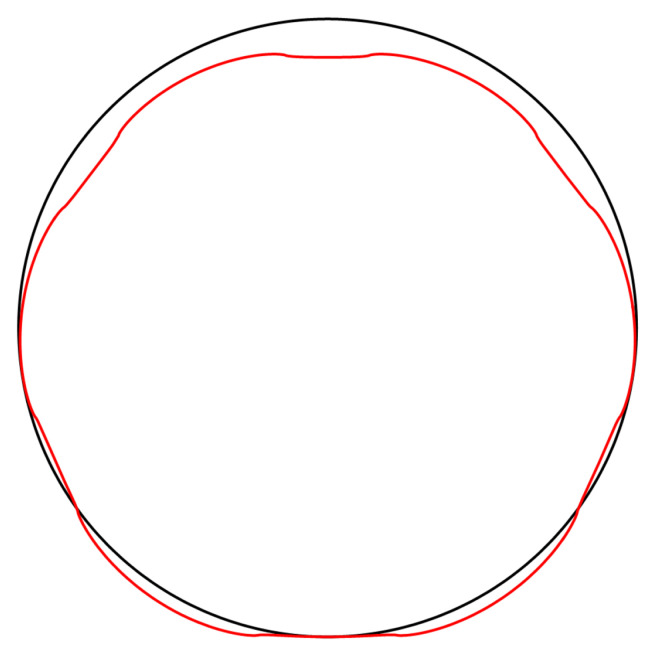
Deformation for the case of a waveguide squeezed between rigid planes of finite width as in Figure 1.2d, drawn in red. The undeformed reference is drawn in black.

All the sums converge, leading to a finite displacement field. The stresses and the displacement are seen in Figure 4.14 and Figure 4.15.

## 5 Estimates for GRAVITES

The planned GRAVITES experiment [ 1] will use single-mode optical fibers made of silica glass, which we assume to be homogeneous, with material properties and experimental parameters given in Tables 5.1– 5.2. The numbers used below, such as the change of pressure *δ* and the change of temperature *T*− *T*_0_, and their outcome for the experiment, correspond to a change in height of 1 m at sea level for an unshielded experiment.

**Table 5.1.** Properties of silica oxide glass from [ 11].

*ν*	*E*	*ρ*	*α*
0.17	73.1 GPa	2.2 g/cm ^3^	1.8 · 10 ^−7^ K ^−1^

**Table 5.2.** Parameters for GRAVITES from [ 1].

*a*	*δ*g	*δp*	*T − T*_0_	*L*	*β*
62.5 µm	3 µm/s ^2^	10 Pa	10 ^–2^ K	10 ^5^ m	6 · 10 ^6^ m ^–1^

It should be kept in mind that the real experiment will be in a vacuum chamber. There are various outcomes possible, depending upon the details of the experimental implementation of the problem:

1. The effects scale in an obvious way with the residual pressure, in which case our calculations provide bounds on the quality of the vacuum needed for measurability of the gravitational effect.
2. The gradients of the temperature of the residual gas affect the temperature of the waveguide during the timescale of the experiment, in which case our calculations provide bounds on the residual temperature gradients.

The above can of course be circumvented by ensuring that there are neither pressure gradients nor temperature gradients, of the kind considered here, inside the vacuum chamber at the timescale of the experiment, so that the effects determined here become irrelevant. Our calculations thus give guidance concerning the experimental setup.

In what follows we will also need to estimate the spooling force. We choose


$$F_{T}=1N,$$

which appears to be standard for commercially available spooling machines. In a first very rough estimate the lateral pressures $\hat{P}$ and $\overset{\vee }{P}$ , for the hexagonal case, are taken to be the same as $\bar{P}$ from the quadrilateral case, as given in ( A.2).

Finally, the maximal pressure $\tilde{P}$ for fibers at the bottom can be estimated by the weight of the spooled fiber *~* 10 kg distributed over the area of a horizontal cross-section of the spool, resulting in


$${\tilde{P}}_{\text{max}}=1.3\text{kPa}.$$

The actual value of $\tilde{P}$ decreases with height along the spool, vanishing for the top layer.

 For the following numeric estimates we use this maximal value.

The GRAVITES estimates for the averaged differences of stresses are denoted by,


$$\;\delta {\bar{\sigma }}_{rr}:=\frac{1}{\pi a^{2}}\int_{U}\left|\sigma_{rr}(r,\theta ){|}_{@1\;\text{m}}-\sigma_{rr}(r,\theta ){|}_{@0\;\text{m}}\right|r\;\text{d}r\;\text{d}\theta ,\;(5.1)$$

the averaged difference of displacements at the boundary are written as


$$\;\delta \bar{u}=\frac{1}{2\pi }\int_{\partial U}\|u{|}_{@1\;\text{m}}-u{|}_{@0\;\text{m}}\text{‖}\;\text{d}\theta ,\;(5.2)$$

where *u* = *u _r_ e _r_* + *u _θ_ e _θ_* . We estimate the change in fiber length as


$$\;\delta L=L\left[-\frac{\nu }{E}(\delta {\bar{\sigma }}_{rr}+\delta {\bar{\sigma }}_{\theta \theta })+\alpha (T-T_{0})\right],\;(5.3)$$

and we use


δφ=βδL(5.4)

to denote the change of phase of light due to the change of length of the waveguide. The results are summarised in Table 5.3 for the single and double plane cases covered in Sections 4.2.1 and 4.2.2. The configurations with four and six contact planes only give small corrections compared to the two plane case, since the additional pressures are independent of the height. This should be compared with the gravitational effect

**Table 5.3.** Estimates for the GRAVITES experiment described in [ 1]. The numbers correspond to a difference of height between the arms of 1 m. The columns isolate the effects with respect to their sources. We write *δ*g ^single^ for the effect calculated in the configuration of Section 4.2.1 and *δ*g ^double^ for that in Section 4.2.2; *δ* $\bar{\sigma }$ _*rr*_ is defined in ( 5.1); *δ* $\bar{u}$ is the average transverse deformation of the waveguide as defined in ( 5.2); *δφ* is the change of phase of light due to the elastic elongation of the waveguide.

	*δ*g ^single^	*δ*g ^double^	*δ* = 10 Pa	*δT* = 10 ^–2^ K
*δ* $\bar{\sigma }$ _*rr*_[Pa]	2.5 · 10 ^–7^	3.4 · 10 ^–7^	10	0
*δ* $\bar{u}$ [µm]	8.3 · 10 ^–15^	9.6 · 10 ^–15^	8.4 · 10 ^–9^	1.7 · 10–7
*δL*[µm]	1.2 · 10 ^–7^	1.6 · 10 ^–7^	4.7	180
*δφ*[rad]	7.0 · 10 ^–7^	9.4 · 10 ^–7^	28	1080


$$\;\;(5.5)$$

in the expected GRAVITES signal.

It has been proposed to place the interferometer in a centrifuge, with horizontal plane of rotation, with the arms of the interferometer placed at different distances from the center of the centrifuge, achieving an acceleration gradient *δ*g *≈* 10 m/s ^2^ between the arms. To estimate the difference of phase between the arms we invoke the equivalence principle, modelling this problem by a radial gravitational field directed away from the axis of rotation of the centrifuge, with strength depending upon the distance from the axis, and ignoring the Earth gravitational field which acts with constant strength normal to the plane of rotation. (Clearly a more precise model would be needed for the real experiment.) For future reference, the corresponding values are given in Table 5.4; we do not include effects related to the change of temperature or pressure there, as such effects would depend strongly upon the experimental setup.

**Table 5.4.** Estimates for a centrifuge experiment with *δ*g = 10 m/s ^2^ at one meter separation. Notation as in Table 5.3.

	*δ*g ^single^	*δ*g ^double^
*δ* $\bar{\sigma }$ [Pa]	0.8	1.8
*δ* $\bar{u}$ [µm]	2.3 · 10 ^−8^	3.8 · 10 ^−8^
*δL*[µm]	0.4	0.8
*δφ*[rad]	2.3	4.8

### A   Relation between $\bar{P}$ and *κ*

The pressure terms $\bar{P}$ in the quadrilateral configuration, as well as $\bar{P}$ and $\overset{\vee }{P}$ in the hexagonal configuration are linked to the spooling tension. We make this relation explicit for the case of a waveguide pressing against a rigid cylinder of radius *R*.

We assume a static configuration, and we ignore boundary effects at the point where the contact between the waveguide and the spool is lost. We consider a waveguide, consisting of an optical fiber, which is wound *N* times in one layer around a rigid cylinder of radius *R*. We suppose that the contact interface of the waveguide and the cylinder has constant width *w*, and that the pressure, which we denote by $\bar{P}$ , exerted on the cylinder by the waveguide is constant across the whole area of contact. We assume that the axis of the cylinder is vertical, and we are interested in the pressure felt by the waveguide at the contact interface between the waveguide and the cylinder.

In a real-life situation there would be several layers of the waveguide, with various radii, with only the lowest one in contact with the cylinder, and the further ones in contact with neighbouring layers. Here we only consider the first layer, which is in contact with the cylinder. Similar considerations can be applied to describe successive layers; one would then also need to take into account the fact that the neighbouring layers are elastic and not rigid, as well as deformations of the neighbouring layers arising from the Poisson ratio. Our analysis in the main body of the paper sets the ground for further such investigations, which are left to future work.

**Figure A.1.**
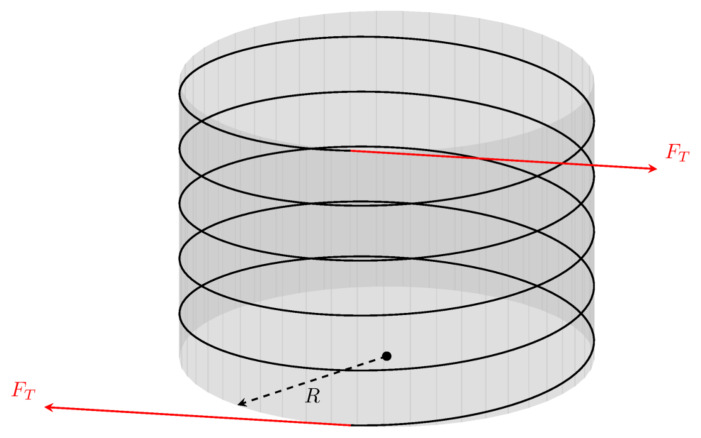
A waveguide wound around a rigid cylinder with radius *R*. We show a single layer of waveguide wrapped around the spool. Equal tension forces are applied at both the starting and end points so that the spool is in equilibrium. The spacing between the neighboring sections of the fiber has been increased for visual clarity.

**Figure A.2.**
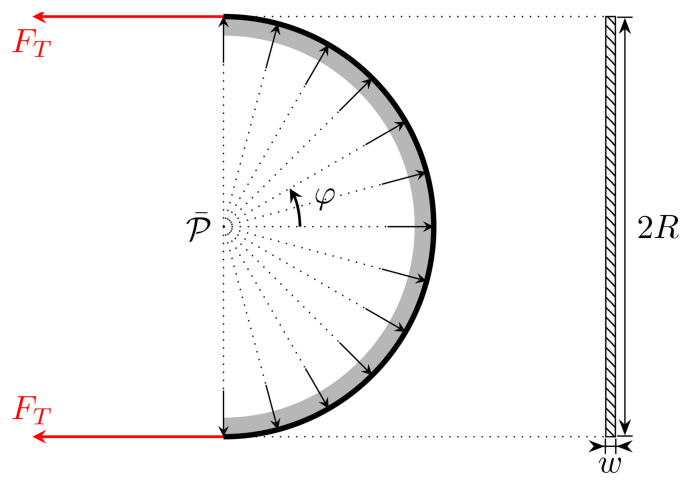
Free-body diagram of half of a single turn of waveguide around the spool (this corresponds to a view from above in Figure 1.2c or Figure A.1, so that the vertical is orthogonal to the plane of the picture). The rectangle on the right-hand side shows the contact area with the width *w* as viewed from the left. The arrows illustrate the direction of the (uniform) reaction force.

We relate the pressure $\bar{P}$ of Figure 1.2c to the tension force *F _T_* by considering the free-body diagram of a half of a single turn of a waveguide, i.e., we consider the intersection of the spool together with the waveguide and a plane on which the axis of the spool lies. The left Figure A.2 shows the intersection when viewed from above the spool of Figure A.1.

Let us imagine that we have a constant pressure $\bar{P}$ acting from the side as in Figure 1.2c, generating a force orthogonal to the contact region with the spool. This pressure generates a force *ℱ* which should equate the tension forces, i.e., *ℱ* = 2 *F _T_*, where


$$\;ℱ\;\text{:=}\;\int_{-\pi /2}^{\pi /2}\bar{P}\;\text{cos}\;\varphi \;A\;\text{d}\varphi =2Rw\;\int_{-\pi /2}^{\pi /2}\bar{P}\;\text{cos}\;\varphi \;\text{d}\varphi \;(\text{A.1})$$

is the normal force due to the pressure $\bar{P}$ acting on the contact area and *φ* is the angle shown in Figure A.2 cover 180 degrees of a semicircle. Hence, *ℱ* is the effective force caused by the pressure $\bar{P}$ .

We assume that the tension force is constant along the waveguide. Therefore, we find *ℱ* = 4 *Rw* $\bar{P}$ , which after equating the forces yields


$$\;\bar{P}=\frac{F_{T}}{2Rw}.\;(\text{A.2})$$

Recall that the constant appearing in the Hertz constant problem is defined as the total force per unit length pushing the bodies into each other, which in our context translates to $\bar{P}$ = / *w*. Comparing with ( A.2) we find


$$\;F=\frac{F_{T}}{2R}.\;(\text{A.3})$$

(Note that this is independent of the number of windings in the spool, and that a virtual work calculation gives the same result.) This value of can now be used in Section 4.2 to calculate *w* by solving the contact problem.

This formula is valid for the first layer, as counted starting from the core of the spool. One can generalize the result for the case where we have more layers that exert forces upon each other by writing


$$\;{\bar{P}}_{n}-{\bar{P}}_{n+1}=\frac{F_{T}}{2[R+(n-1)a]w_{n}},\;(\text{A.4})$$

where *w _n_* is the contact width at the *n*’th layer, while $\bar{P}$ _*n*_ and $\bar{P}$ _*n*+1_ are the contact pressures there.

### B   Extending to non-symmetric solutions

Although the mirror symmetry assumption made in Section 2 is well-motivated for our purposes, we nevertheless give the full solution for completeness, demanding only regularity at *r* = 0 and 2 *π*-periodicity in *θ*.

Discarding only terms incompatible with regularity and 2 *π*-periodicity in ( 2.25), as well as *B*_0_, *D*_1_ and *d*_1_ which do not contribute to the stresses, we have


$$\;\begin{array}{l} \phi (r,\theta )=D_{0}r^{2}+C_{1}r^{3}\text{cos(}\theta \text{)}+c_{1}r^{3}\text{sin(}\theta \text{)}\\ \;+\sum_{n\geq 2}\left[\left(A_{n}r^{n}+C_{n}r^{n+2}\right)\text{cos(}n\theta )+\left(a_{n}r^{n}+c_{n}r^{n+2}\right)\text{sin(}n\theta \text{)}\right].\;(\text{B.1}) \end{array}$$

Again, the displacement can be derived by integrating ( 2.21)–( 2.23), yielding


$$\;\begin{array}{l} u_{r}(r,\theta )=\frac{1}{2\mu }\{2(1-2\nu )D_{0}r-\frac{1}{2}\text{g}\rho (1-2\nu )r^{2}\text{cos}(\theta )+(1-4\nu )r^{2}(C_{1}\text{cos}(\theta )+c_{1}\;\text{sin}(\theta ))\\ \;+\sum_{n\geq 2}(\left[-nA_{n}r^{n-1}+(2-4\nu -n)C_{n}r^{n+1}\right]\text{cos(}n\theta \text{)}\\ \;+\left[-na_{n}r^{n-1}+(2-4\nu -n)c_{n}r^{n+1}\right]\text{sin(}n\theta \text{)})\}\\ \;-\text{Ξ}\;\text{cos(}\theta \text{)}+{\text{Ξ}}_{2}\text{sin(}\theta \text{)}-\nu \kappa r+(1+\nu )\alpha (T-T_{0})r,\;(\text{B.2}) \end{array}$$

$$\;\begin{array}{l} u_{\theta }(r,\theta )=\frac{1}{2\mu }\{\left(5-4\nu )r^{2}(C_{1}\;\text{sin}(\theta )+c_{1}\;\text{cos}(\theta ))-\frac{1}{2}\text{g}\rho (1-2\nu )r^{2}\;\text{sin}(\theta \right)\\ \;+\sum_{n\geq 2}(\left[nA_{n}r^{n-1}+(4-4\nu +n)C_{n}r^{n+1}\right]\text{sin(}n\theta \text{)}\\ \;\;+\left[-na_{n}r^{n-1}-(4-4\nu +n)c_{n}r^{n+1}\right]\text{cos(}n\theta \text{)})\}\\ \;+\text{Ξ}\;\text{sin(}\theta )+{\text{Ξ}}_{2}\text{cos(}\theta )+c^{*}r,\;(\text{B.3}) \end{array}$$

with the integration constants determined by the boundary conditions *u _r_* ( *a*, 0) = 0 = *u _θ_* ( *a*, 0) as above. Thus,


$$\;\begin{array}{l} \text{Ξ}=\frac{1}{2\mu }\{2(1-2\nu )D_{0}a+(1-4\nu )C_{1}a^{2}-\frac{1}{2}\text{g}\rho (1-2\nu )a^{2}\\ \;+\sum_{n\geq 2}\left[-nA_{n}a^{n-1}+(2-4\nu -n)C_{n}a^{n+1}\right]\}-\nu \kappa a+(1+\nu )\alpha (T-T_{0})a,\;(\text{B.4}) \end{array}$$

and further


$$\;{\text{Ξ}}_{2}=\frac{1}{2\mu }\left\{(5-4\nu )a^{2}c_{1}+\sum_{n\geq 2}\left[na_{n}a^{n-1}+(4-4\nu +n)c_{n}a^{n+1}\right]\right\}-c^{*}a,\;(\text{B.5})$$

provided that the sums converge.

Continuing to the boundary conditions, we immediately restrict to the frictionless case, taking


$$\;\sigma_{r\theta }{\text{|}}_{\partial U}=0.\;(\text{B.6})$$

The boundary condition for *σ _rr_* can now be a general Fourier series which we write in the suggestive form


$$\;\sigma_{rr}{\text{|}}_{\partial U}=f(\theta )=f_{0}+\sum_{n\geq 1}[(f_{-n}+f_{n})\text{cos}(n\theta )+i(f_{-n}-f_{n})\text{sin}(n\theta )],\;(\text{B.7})$$

with


$$\;f_{n}=\frac{1}{2\pi }\int_{-\pi }^{\pi }\text{d}\theta \;f\text{(}\theta \text{)}e^{in\theta }.\;(\text{B.8})$$

These boundary conditions determine the coefficients in ( B.1) algebraically by comparing to the corresponding stresses ( 2.14)–( 2.16). We find


$$\;C_{1}=c_{1}=0,\;C_{n}=\frac{1-n}{a^{2}(1+n)}A_{n}\;\text{and}\;c_{n}=\frac{1-n}{a^{2}(1+n)}a_{n},\;(\text{B.9})$$

via ( B.6), leading to


$$\;\sigma_{rr}{\text{|}}_{\partial U}=2D_{0}-a\text{g}\rho \;\text{cos(}\theta \text{)}\;+\sum_{n\geq 2}2(1-n)a^{n-2}[\text{sin}(n\theta )a_{n}+\text{cos}(n\theta )A_{n}],\;(\text{B.10})$$

and comparing with ( B.7) for the remaining coefficients


$$\;D_{0}=\frac{1}{2}f_{0},\;(B.11)$$

$$\;A_{n}=-\frac{1}{2(n-1)}a^{2-n}(f_{-n}+f_{n}),\;(B.12)$$

$$\;a_{n}=-\frac{i}{2(n-1)}a^{2-n}(f_{-n}-f_{n}),\;(B.13)$$

for *n* ≥ 2, as well as


$$\;f_{-1}+f_{1}=-ag\rho ,\;(B.14)$$

$$\;f_{-1}-f_{1}=0.\;(B.15)$$

Note that, similarly to the symmetric case the space of possible boundary conditions is constrained by the body force. Without any body forces oriented along the *x*-direction ( B.15) requires that the sin( *θ*) term in ( B.7) vanishes.

In the simplest case, considering line forces from the left and right and a line support from the bottom, i.e. boundary conditions


$$\;\sigma_{r\theta }{|}_{\partial U}=0,\;(B.16)$$

$$\;\sigma_{rr}{|}_{\partial U}=\delta (\theta )+\hat{P}\delta (\theta -\pi /2)+\overset{\vee }{P}\delta (\theta +\pi /2),\;(B.17)$$

Equation ( B.15) implies $\hat{P}$ = $\overset{\vee }{P}$ , thus forcing mirror symmetry.

## Data Availability

No data are associated with this article.
