# On elastic deformations of cylindrical bodies under the influence of the gravitational field

**DOI:** 10.12688/openreseurope.17329.2

**Published:** 2024-12-04

**Authors:** Hamed Barzegar, Piotr T. Chruściel, Elisabeth Steininger

**Affiliations:** 1 Centre de Recherche Astrophysique de Lyon, Universite Claude Bernard Lyon 1, Villeurbanne, Auvergne-Rhône-Alpes, 69622, France; 2 Faculty of Physics, University of Vienna, Vienna, Vienna, 1090, Austria

**Keywords:** linear elasticity, GRAVITES, waveguides, Airy stress, Michell solution

## Abstract

**Background:**

Elastic deformations of gravitating cylindrical bodies are relevant for state-of-the-art photonic experiments, as they affect the physical properties of materials under consideration, impacting wave propagation. This is of key importance for a recently planned experiment to explore the influence of the gravitational field on entangled photons propagating in waveguides. The purpose of this work is to determine these elastic deformations as functions of temperature, pressure, and of the gravitational field. We thus determine the deformations of the body due to changes of the gravitational field, and obtain stringent bounds on the control of temperature and pressure so that the effects of the associated elastic deformations on the photons propagating in a waveguide are smaller than the phase shifts associated with the change of the gravitational field.

**Methods:**

We use the methods of linear elasticity, including thermoelasticity, to determine the stresses and strains of the medium. For this, the symmetry of the cylinder allows us to solve the problem by using Mitchell’s solutions of the equations satisfied by the Airy functions. The boundary conditions are implemented by an approximation of the Hertz contact method.

**Results:**

We calculate the displacements, the stresses and strains for several classes of boundary conditions, and give explicit solutions for a number of physically motivated configurations. The influence of the resulting deformations on the planned GRAVITES experiment is determined.

**Conclusions:**

The results are relevant for fiber interferometry experiments sensitive to the effects of the gravitational field on photon propagation. Our calculations give stringent bounds on the environmental variables, which need to be controlled in such experiments.

## 1 Introduction

**Figure 1.1.**
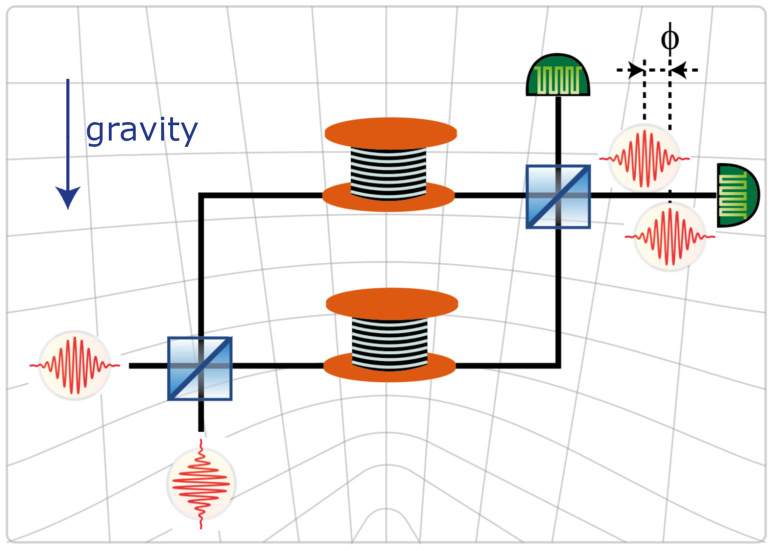
The GRAVITES experiment. The upper arm of the interferometer is moved vertically in the height-dependent gravitational field of Earth, indicated by a curving background. The change of the gravitational field due to the change of height affects the propagation of light, resulting in a height-dependent phase shift *φ*. Both here and in our calculations the *y*-axis is aligned vertically, so that the gravitational force, indicated by the down-pointing arrow, acts anti-colinearly along the *y*-axis. In the experimental configuration the axis of the spool is vertical, in order to minimize the deformations of the spool. The radius of the waveguide is much smaller than the radius of the spool, and the slope of the waveguide arising from the spooling is small. This leads us to an approximation where the waveguide is “unwound” to be straight and horizontal. In our calculations the *z*-direction is aligned with the axis of the unwound waveguide and is horizontal. See also Figure 1.2. © C. Hilweg, reproduced with kind permission of the author.

An experiment is currently being built [ 1] with the aim to measure the effect of the gravitational field on entangled states of photons propagating in an optical fiber. The experiment requires displacing vertically an optical fiber, with circular cross-sections, in a spooled configuration. See Figure 1.1 and Figure 1.3 for the configuration of the interferometer after an arm has been moved to a higher position. Such a displacement in the gravitational field of the Earth leads to a minute phaseshift, which is expected to be measurable with the current state-of-the art photonic technology. The displacement is associated with a change of the ambient gravitational acceleration, of temperature, and of atmospheric pressure, leading to an elastic deformation of the fiber. The deformation affects the shape, the length, and the propagation properties of light in the fiber, leading to an additional phaseshift which needs to be determined for a correct interpretation of the results of the experiment.

The objective of this work is to develop a framework which can be used to determine this elastic deformation. This requires in principle a general relativistic theory of elasticity [ 2– 6]. However, it has been shown in [ 7] that, not unexpectedly, Newtonian elasticity provides a good approximation in the regime we are interested in. The resulting effects on the propagation properties of light in the fiber will be determined elsewhere [ 8].

**Figure 1.2.**
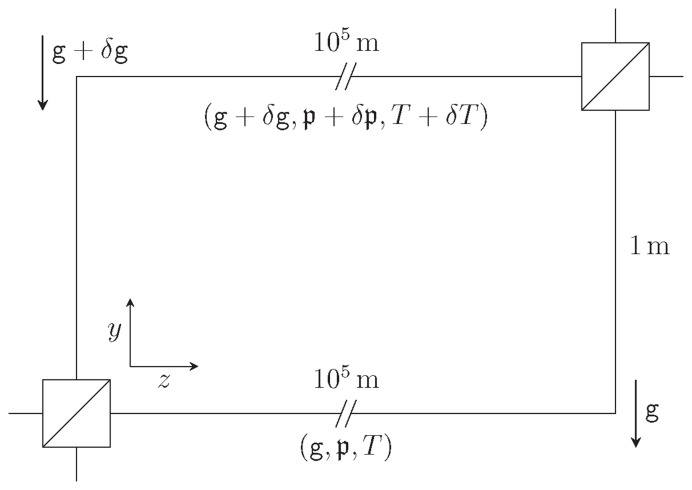
Our model for analysing the elastic deformations in GRAVITES. The spools have been unwound so that the center of the waveguide lies on the axis *x*= *y*=0. Hence we ignore the curving of the spool and the changes of physical parameters as we move along each individual arm. The coordinate *y* runs along the vertical. The coordinate *z* runs along the waveguide.

**Figure 1.3.**
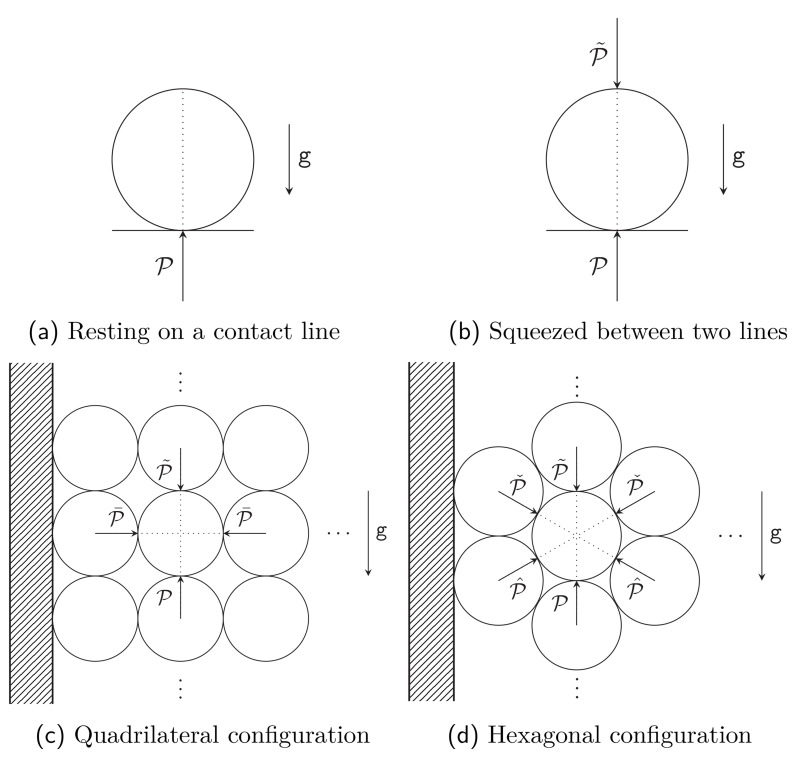
The four main models. The *y*-axis is again along the vertical in all four figures. Figures ( **a**) and ( **b**) show the cross-section of an infinite cylinder resting on a rigid support, with a supplementary pressure from the top in Figure ( **b**). Figures ( **c**) and ( **d**) show the spool of Figure 1.1 as cut by a vertical plane; we assume that the radius of the spool is very large compared to the radius of the waveguide. The circles represent the consecutive returns of the waveguide, exercising pressure on the neighboring strands. The dashed region represents the rigid spool.

The planned configuration is that of a spool with its axis of symmetry aligned vertically. Since the radius of the spool is very large compared to the radius of the fiber, we ignore the spooling and consider a very long elastic cylinder. We then consider several models for the problem at hand:

As a first model we start with a cylindrical waveguide resting on a horizontal contact line (see Figure 1.3a and Section 4.1.1). The next model is a configuration where the waveguide is squeezed between two contact lines, to take into account the pressure arising from the layers pressing from above (see Figure 1.3b and Section 4.1.2). The results obtained in both cases are unacceptable, with an infinite deformation at each contact line; this is of course a well known problem of such models (cf. [ 9, Chapter 8.4.7]). However, the model appears useful for its simplicity, we will return to this shortly.

To avoid the above problems we pass to a Hertz-contact-type calculation, where we first analyse a configuration with the waveguide resting on a rigid support, followed by one where the waveguides are stacked upon each other; in the last case the influence of the upper layer on a lower one is modelled by contact with a rigid plane. There arises a parameter describing the pressure from the waveguides stacked above the section of the fiber under consideration. We leave this parameter free in our calculations, its value can be determined by the number of layers and windings of the spool whenever a specific configuration is considered.

The above takes into account the vertical neighbours of any given section of the fiber, but ignores the side neighbours. To address this we consider two further configurations, where each fiber has four neighbours as in Figure 1.3c (see Section 4.2.4), or each fiber has six neighbours as in Figure 1.3d and Section 4.2.5. The last model seems to us to provide the best approximation to the problem at hand. In such models new parameters arise, associated with presence of new neighbours. We leave these parameters free again, and show in Appendix A how one of these parameters can be determined for each layer in the quadrilateral-contact case.

Our results show that in all models the relative deformations are similar, for the practical purposes of our interest, close enough to the center of waveguide, where the guiding core resides. Therefore we expect that the first, simplest model, will be sufficient for the applications we have in mind. We address this question in a future full treatment of the influence of small elastic deformations on the dispersion relation in optical fibers [ 8].

We include a constant ambient pressure term, as well as temperature effects in all configurations, in order to model possible variations of the environment.

## 2 Waveguide deformation in Earth’s gravity

**Figure 2.1.**
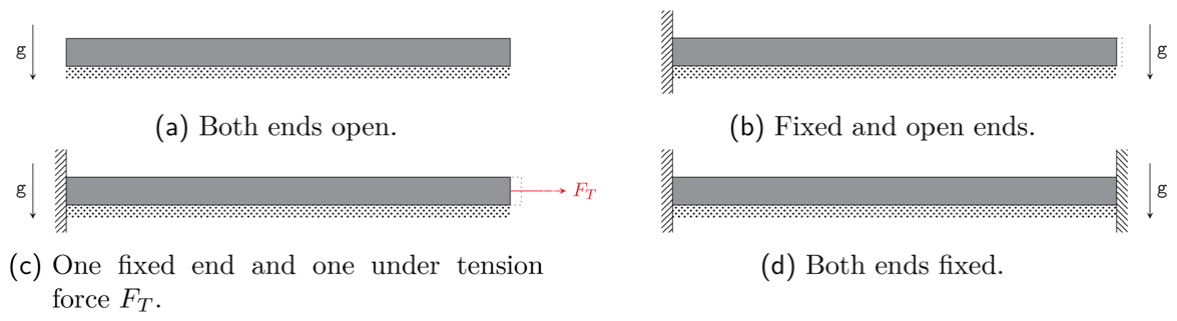
Four models for a very long cylinder with boundary conditions covered by our calculations. Vertical dashed regions represent fixed ends in the *z*-direction, whereas the dotted bars represent rigid supports. The remaining boundaries in the figures can move freely. The presence of a tension force *F _T_* in Figure (c) is relevant for the boundary conditions in the *z*-direction and its effect will be analysed in Section 3. Note that the model ( **d**) requires the vanishing of the elongation coefficient *κ* of ( 2.6).


The unperturbed waveguide is modelled as a very long, homogeneous cylinder with radius *a*, length *L* and density *ρ*, supported by a rigid plane with gravity acting as a body force via a constant gravitational acceleration g. We take *U* = {( *r, θ, z*) : 0 *< r ≤ a, –π < θ ≤ π,* 0 *≤ z ≤ L*} to be the interior of the waveguide and write *∂U* for the surface *r* = *a*. Note that *a ⪡ L*. We will consider the four configurations depicted in Figure 2.1 (a)–(d). The elastic deformations that are of interest to us are minute, therefore we will apply the linear theory of elastic deformations, including thermoelasticity. Such deformations are described by a field *u _i_* which describes the (small) displacements of a body part from a position which arises when no forces are applied on the system. One then defines the associated *strain tensor*:


$$\;\epsilon_{ij}=\frac{1}{2}(\partial_{i}u_{j}+\partial_{j}u_{i}).\;(2.1)$$

This induces stresses in the material, described by the stress tensor *σ _ij_*. Assuming isotropy, the generalized Hooke law in three dimensions takes the form of the following stress-strain relation (cf., e.g., [ 9, Eq. (4.2.7)] and [ 10, Eq. ( 4.6)]),


$$\;\sigma_{ij}=\lambda \epsilon_{kk}\delta_{ij}+2\mu \epsilon_{ij},\;(2.2)$$

Here *λ* and *µ* are respectively Lamé’s first and second parameters of the material. The parameter *µ*, called the shear modulus, is sometimes denoted by *G* in the literature. We only consider homogeneous materials, where the Lamé parameters are taken to be constant throughout the material. Repeated indices are summed over unless explicitly indicated otherwise. Here, and elsewhere, all tensors are expressed in orthonormal frames. With *σ _kk_* = (3 *λ* + 2 *µ*) *ϵ _kk_* we find


$$\;\epsilon_{ij}=\frac{1+\nu }{E}\sigma_{ij}-\frac{\nu }{E}\sigma_{kk}\delta_{ij},\;(2.3)$$

where *E* := *µ*(3 *λ* + 2 *µ*) */*( *λ* + *µ*) is Young’s modulus and *ν* := *λ/*[2( *λ* + *µ*)] is Poisson’s ratio. Note that *E* = 2 *µ*(1 + *ν*).

When the changes of temperature are *not* negligible, the above needs to be revised as follows: According to [ 9, Section 4.4], in a thermally-isotropic and thermally-linear medium, Equation (2.3) should be replaced by


$$\;\epsilon_{ij}=\frac{1+\nu }{E}\sigma_{ij}-\frac{\nu }{E}\sigma_{kk}\delta_{ij}+\alpha (T-T_{0})\delta_{ij},\;(2.4)$$

where *α* is the *coefficient of linear thermal expansion*, which we assume to be constant throughout the material. This is a consequence of the assumptions of linear thermo-elasticity, for which the strain decomposes into independent thermal and elastic components. Inverting this relation, the stress tensor is given by


$$\;\sigma_{ij}=\lambda \epsilon_{kk}\delta_{ij}+2\mu \epsilon_{ij}-(3\lambda +2\mu )\alpha (T-T_{0})\delta_{ij}.\;(2.5)$$

We allow the waveguide to stretch in the *z* direction linearly in *z*,

$$\;u_{z}=\kappa z.\;(2.6)$$

All remaining fields are assumed to be *z*-independent. While we will consider all four configurations in Figure 2.1, the one that we are most interested in is configuration ( **c**). In that case we have displacement-free boundary conditions on one end while needing to match the stress at the other end to the externally imposed force *F _T_*. We assume that *F _T_* remains constant when moving the spools in the gravitational field, and its precise value drops out when calculating the changes of the deformations (cf. Equation (3.17) below).

Equation (2.6) leads to a non-vanishing *z*-component of the strain tensor, namely


$$\;\epsilon_{zz}=\kappa ,\;\text{but}\;\epsilon_{xz}=\epsilon_{yz}=0,\;(2.7)$$

giving an additional contribution to the usual plane-strain relations (cf. [ 11] for related considerations).

Having reduced the dimensionality of the problem, the equilibrium equations (cf., e.g., [ 9, Eq. (3.6.4)] or [ 12, Eq. (2.34)]),


$$\;\partial_{j}\sigma_{ij}+F_{i}=0,\;(2.8)$$

with *σ _ij_* the stress tensor and *F _i_* = *−∂ _i_V* the body force due to gravity, where *V* = g *ρy*, simplify to


$$\;\partial_{x}\sigma_{xx}+\partial_{y}\sigma_{xy}-\partial_{x}V=0,\;(2.9)$$

$$\;\partial_{x}\sigma_{xy}+\partial_{y}\sigma_{yy}-\partial_{y}V=0.\;(2.10)$$

where we used Cartesian coordinates ( *x*, *y*, *z*), in which the Euclidean metric equals *dx* ^2^ + *dy* ^2^ + *dz* ^2^. Note that the stress in the *z*-direction does not necessarily vanish. Indeed, the *zz*-component of ( 2.4) gives


$$\;\kappa =\frac{\sigma_{zz}}{E}-\frac{\nu }{E}(\sigma_{xx}+\sigma_{yy})+\alpha (T-T_{0}),\;(2.11)$$

so that all the elasticity equations, including Equation (2.8) will be satisfied by setting *σ*_*zx*_ = *σ*_*zy*_ = 0 and


$$\;\sigma_{zz}=\nu (\sigma_{xx}+\sigma_{yy})+E\kappa -E\alpha (T-T_{0}).\;(2.12)$$

We wish to solve Equation (2.9)– Equation (2.10) for gravitating systems which include the GRAVITES experiment. For this we will use the fact that solutions of these equations can be parameterised by the *Airy stress function ϕ* (cf. [ 13] and, e.g., [ 14, Eqs. (8.12)–(8.13)]) as


$$\;\sigma_{ij}=\left({\text{Δ}}_{\delta }\phi +V\right)\delta_{ij}-D_{i}D_{j},\;(2.13)$$

where *D _i_* denotes the covariant derivative of the Euclidean metric *dx* ^2^ + *dy* ^2^ = *dr* ^2^ + *r* ^2^ *dθ* ^2^ on ℝ ^2^, and Δ _*δ*_ its Laplacian (the formula with *V* = 0 parameterises thus all divergence-free symmetric tensors).

Adapting our coordinate system to the symmetry of the setup by choosing cylindrical coordinates, from now on tensor components will refer to the following orthonormal frame, which in Cartesian coordinates is given by


$$\;e_{r}\;\text{:=}\;(\cos(\theta ),\;\sin(\theta ),0),\;e_{\theta }\;\text{:=}\;(\text{−}\sin(\theta ),\;\cos(\theta ),0),\;e_{z}\;\text{:=}\;(0,0,1).\;(2.14)$$

In this frame the stress tensor can be expressed in terms of derivatives of the Airy stress function *ϕ* and of the gravitational potential *V* as


$$\;\sigma_{rr}=\frac{1}{r}\partial_{r}\phi +\frac{1}{r^{2}}\partial_{\theta }^{2}\phi +V,\;(2.15)$$

$$\;\sigma_{\theta \theta }=\partial_{r}^{2}\phi +V,\;(2.16)$$

$$\;\sigma_{r\theta }=\text{−}\partial_{r}(\frac{1}{r}\partial_{\theta }\phi ),\;(2.17)$$

The Airy function has to satisfy the "compatibility condition" (see, e.g., [ 6, Proposition 4.31], [ 9, Eq. (7.5.5) or [ 12, Eq. (7.17b)], together with [ 9, Eq. (12.3.7)]),

$$\;{\text{Δ}}_{\delta }^{2}\phi =\text{−}\frac{1-2\nu }{1-\nu }{\text{Δ}}_{\delta }V-\frac{E\alpha }{1-\nu }{\text{Δ}}_{\delta }T,\;(2.18)$$

(Note that *V* is only defined up to a constant, which can be absorbed by a redefinition of *ϕ*.) In this work we consider a steady-state configuration, which requires Δ *_δ_T* = 0 (cf. [ 9, Section 12.1]). Further, since *V* = g *ρy* we have Δ *_δ_V* = 0 as well, implying that the compatibility conditions reduce to the homogeneous biharmonic equation.

Since the frame { *e _r_, e _θ_, e _z_*} is orthonormal, the strain is related to the stress via ( 2.4):


$$\;\epsilon_{rr}=\frac{1}{2\mu }[(1-\nu )\sigma_{rr}-\nu \sigma_{\theta \theta }]-\nu \kappa +(1+\nu )\alpha (T-T_{0}),\;(2.19)$$

$$\;\epsilon_{\theta \theta }=\frac{1}{2\mu }[(1-\nu )\sigma_{\theta \theta }-\nu \sigma_{rr}]-\nu \kappa +(1+\nu )\alpha (T-T_{0}),\;(2.20)$$

$$\;\epsilon_{r\theta }=\frac{1}{2\mu }\sigma_{r\theta }.\;(2.21)$$

One can now determine the displacement vector *u _i_* from the usual equations, where *u _r_* and *u _θ_* are frame components of *u* (cf., e.g., [ 9, Eq. (7.6.1)])


$$\;\epsilon_{rr}=\partial_{r}u_{r},\;(2.22)$$

$$\;\epsilon_{\theta \theta }=\frac{1}{r}(\partial_{\theta }u_{\theta }+u_{r}),\;(2.23)$$

$$\;\epsilon_{r\theta }=\frac{1}{2}\left(\frac{1}{r}\partial_{\theta }u_{r}+\partial_{r}u_{\theta }-\frac{1}{r}u_{\theta }\right).\;(2.24)$$

**Figure 2.2.**
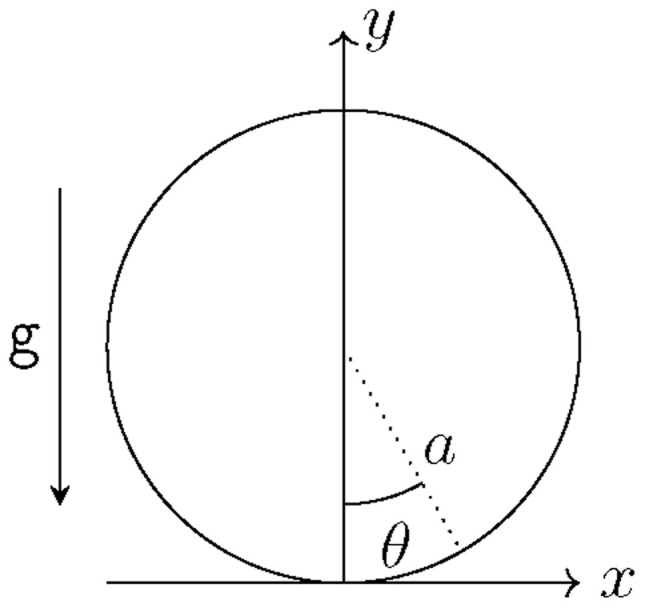
Convention for polar coordinates.

Having compiled all relevant equations from linear elasticity, we set up an adapted coordinate system as in Figure 2.2, with 


r∈(0,a]andθ∈(−π,π].(2.25)

There exists a general solution for ( 2.18) in polar coordinates, denoted by ^1^


$$\;\begin{array}{l} \phi (r,\theta )=A_{0}\;\text{ln}\;r+B_{0}+C_{0}r^{2}\;\text{ln}\;r+D_{0}r^{2}\\ \;+\left(a_{0}\;\text{ln}\;r+b_{0}+c_{0}r^{2}\;\text{ln}\;r+d_{0}r^{2}\right)\theta \\ \;+\left(A_{1}r\;\text{ln}\;r+\frac{B_{1}}{r}+C_{1}r^{3}+D_{1}r+E_{1}r\theta +F_{1}r\theta \;\text{ln}\;r\right)\text{cos(}\theta \text{)}\\ \;+\left(a_{1}r\;\text{ln}\;r+\frac{b_{1}}{r}+c_{1}r^{3}+d_{1}r+e_{1}r\theta +f_{1}r\theta \;\text{ln}\;r\right)\text{sin(}\theta \text{)}\\ \;+\sum_{n\geq 2}[\left(A_{n}r^{n}+B_{n}r^{-n}+C_{n}r^{n+2}+D_{n}r^{2-n}\right)\text{cos(}n\theta \text{)}\\ \;+\left(a_{n}r^{n}+b_{n}r^{-n}+c_{n}r^{n+2}+d_{n}r^{2-n}\right)\text{sin(}n\theta \text{)}],\;(2.26) \end{array}$$

known as the ‘Michell solution’ [ 15].

In practical terms, we are mostly concerned with determining the coefficients in ( 2.26) from the boundary conditions we impose. An immediate simplification can be achieved by only considering solutions which have the mirror symmetry *x* ↦ *−x* (equivalently *θ* ↦ *−θ*), since the forces considered are invariant under this symmetry; see Appendix B for a treatment without imposing mirror symmetry at the outset. Additionally, we require regularity at *r* = 0 and 2 *π*-periodicity in *θ*. Restricting to mirror-symmetric boundary conditions finally leads to a requirement of mirror-symmetry in *σ _rr_* and *σ _θθ_* and antisymmetry for *σ _rθ_*. Lastly, note that the parameters *B*_0_ and *D*_1_ do not contribute to the stress as by Equation (2.15)– Equation (2.17), which is straightforwardly verified. They may be considered degeneracies of the solution space and set to zero without loss of generality (cf. [ 14]). The remaining terms in ( 2.26) are


$$\;\phi (r,\theta )=D_{0}r^{2}+C_{1}r^{3}\text{cos(}\theta \text{)}+\sum_{n\geq 2}\left(A_{n}r^{n}+C_{n}r^{n+2}\right)\text{cos(}n\theta \text{),}\;(2.27)$$

which is the form considered for all applications below.

The displacement can be calculated via ( 2.22)–( 2.24) ^2^. We have for the remaining terms in *ϕ*,


$$\;\begin{array}{l} u_{r}(r,\theta )=\frac{1}{2\mu }\{2(1-2\nu )D_{0}r+(1-4\nu )C_{1}r^{2}\cos(\theta )-\frac{1}{2}\text{g}\rho (1-2\nu )r^{2}\cos(\theta )\\ \;+\sum_{n\geq 2}\left[-nA_{n}r^{n-1}+(2-4\nu -n)C_{n}r^{n+1}\right]\text{cos(}n\theta \text{)}\}-\text{Ξ}\;\text{cos(}\theta \text{)}-\nu \kappa r\\ \;+(1+\nu )\alpha (T-T_{0})r,\;(2.28) \end{array}$$

$$\;\begin{array}{l} u_{\theta }(r,\theta )=\frac{1}{2\mu }\{\left(5-4\nu )C_{1}r^{2}\sin(\theta )-\frac{1}{2}\text{g}\rho (1-2\nu )r^{2}\sin(\theta \right)\\ \;+\sum_{n\geq 2}\left[nA_{n}r^{n-1}+(4-4\nu +n)C_{n}r^{n+1}\right]\text{sin(}n\theta \text{)}\}+\text{Ξ}\;\text{sin(}\theta \text{)}+c^{*}r,\;(2.29) \end{array}$$

where *c* ^∗^, Ξ are integration constants. These are fixed by the boundary conditions imposed at the contact point *u _r_* ( *a,* 0) = 0 = *u _θ_* ( *a,* 0), which imply *c* ^∗^ = 0 and


$$\begin{array}{l} \text{Ξ}\;\text{:=}\;\frac{1}{2\mu }\{2(1-2\nu )D_{0}a+(1-4\nu )C_{1}a^{2}-\frac{1}{2}\text{g}\rho (1-2\nu )a^{2}\\ \;+\sum_{n\geq 2}\left[-nA_{n}a^{n-1}+(2-4\nu -n)C_{n}a^{n+1}\right]\}-\nu \kappa a+(1+\nu )\alpha (T-T_{0})a,\;(2.30) \end{array}$$

provided that the sums converge.

## 3 Boundary conditions

We now have general expressions for the stresses ( 2.15)–( 2.17), the strains ( 2.19)–( 2.21), and the displacements ( 2.28)–( 2.29), in terms of the coefficients { *D*_0_, *C*_1_, *A _n_*, *C _n_*} for *n* ≥ 2. These coefficients can be determined from the boundary conditions algebraically.

To proceed further one needs to specify the boundary conditions satisfied by the bodies of interest. At the boundary of the cylinder we consider an angle dependent pressure *f*( *θ*) acting in the radial direction and a shear force per unit area *g*( *θ*). The boundary conditions in linear elasticity require the stresses at the boundary to react to the external forces as 


$$\;\sigma_{r\theta }{\text{|}}_{\partial U}=g(\theta ),\;(3.1)$$

$$\;\sigma_{rr}{\text{|}}_{\partial U}=f(\theta ).\;(3.2)$$

Our assumption of mirror-symmetry implies that the boundary conditions can be Fourier decomposed into sine and cosine series respectively,


$$\;\sigma_{r\theta }{\text{|}}_{\partial U}=\sum_{n\geq 0}g_{n}\text{sin(}n\theta \text{),}\;(3.3)$$

$$\;\sigma_{rr}{\text{|}}_{\partial U}=\frac{1}{2}f_{0}+\sum_{n\geq 1}f_{n}\text{cos(}n\theta \text{),}\;(3.4)$$

with the coefficients given explicitly by


$$\;g_{n}=\frac{2}{\pi }\int_{0}^{\pi }\text{d}\theta \;g\text{(}\theta \text{)sin(}n\theta \text{),}\;(3.5)$$

$$\;f_{n}=\frac{2}{\pi }\int_{0}^{\pi }\text{d}\theta \;f\text{(}\theta \text{)cos(}n\theta )\text{.}\;(3.6)$$

On the other hand, we know that the solution can be written using ( 2.15)–( 2.17) with ( 2.27) for the Airy stress function; explicitly 


$$\;\sigma_{r\theta }{\text{|}}_{\partial U}=2C_{1}a\;\text{sin(}\theta \text{)+}\sum_{n\geq 2}\left[(n-1)A_{n}+a^{2}(n+1)C_{n}\right]na^{n-2}\sin(n\theta ),\;(3.7)$$

$$\;\begin{array}{l} \sigma_{rr}{\text{|}}_{\partial U}=2D_{0}+(2C_{1}-\text{g}\rho )a\;\cos\text{(}\theta \text{)}-\sum_{n\geq 2}\left[(n-1)nA_{n}+a^{2}(n^{2}-n-2)C_{n}\right]a^{n-2}\cos(n\theta ).\;(3.8) \end{array}$$

Comparing term by term determines the coefficients. We are generally interested in the case with vanishing shear forces, i.e. *g*( *θ*) = 0, which corresponds to only normal forces or the frictionless limit. Then,


$$\;C_{1}=0\;\text{and}\;C_{n}=\frac{1-n}{a^{2}(1+n)}A_{n},\;(3.9)$$

and for the radial stress


$$\;\sigma_{rr}{\text{|}}_{\partial U}=2D_{0}-a\text{g}\rho \;\cos\text{(}\theta \text{)}\;-2\sum_{n\geq 2}A_{n}(n-1)a^{n-2}\cos(n\theta ).\;(3.10)$$

Comparing coefficients with ( 3.4), we find 


$$\;D_{0}=\frac{1}{4}f_{0},\;(3.11)$$

$$\;A_{n}=-\frac{1}{2(n-1)}a^{2-n}f_{n},\;(3.12)$$

for *n* ≥ 2, with


$$\;f_{1}=-a\text{g}\rho .\;(3.13)$$

We see that the Fourier coefficient *f*_1_ in the function *f* in ( 3.2) is not arbitrary, and is determined by the body force.

The boundary conditions in the *z*-direction are given by the models shown in Figure 2.1. We focus on subfigure (c), with the special case without tension force *F _T_* corresponding to subfigure (b). By definition, the displacement boundary conditions are given by ( 2.6), since we allow for elongation of the open end. The boundary condition for the stress is given by


$$\;F_{T}=\int_{U(z=L)}\sigma_{zz}r\text{d}r\;\text{d}\theta \text{,}\;(3.14)$$

i.e. the forces on the end face of the cylinder have to match the stresses on the end face. Using ( 2.12), this can be rewritten as


$$\;F_{T}=\int_{U(z=L)}\nu (\sigma_{xx}+\sigma_{yy})r\;\text{d}r\;\text{d}\theta +\pi a^{2}E[\kappa -\alpha (T-T_{0})].\;(3.15)$$

The integral can be calculated either explicitly or numerically for the solutions in Section 4, providing thus a relation between the forces acting on the system, *κ*, and the remaining parameters that appear in the problem.

This can be restated as an equation for the elongation coefficient *κ* as a function of tension *F _T_* and the plane stresses along the fiber:


$$\;\kappa =-\frac{1}{\pi a^{2}E}\int_{U(z=L)}\nu (\sigma_{xx}+\sigma_{yy})r\;\text{d}r\;\text{d}\theta +\alpha (T-T_{0})+\frac{F_{T}}{\pi a^{2}E}.\;(3.16)$$

For a waveguide of total length *L*, this implies a change in length 


$$L\mapsto L+\kappa L=L\left[1-\frac{1}{\pi a^{2}E}\int_{U(z=L)}\nu (\sigma_{xx}+\sigma_{yy})r\;\text{d}r\;\text{d}\theta +\alpha (T-T_{0})+\frac{F_{T}}{\pi a^{2}E}\right].\;(3.17)$$

Note that the length can change even if *F _T_* vanishes.

Given a spooling force and upon specifying ambient gravity, pressure, and temperature, Equation (3.17) allows the computation of the length of each arm of the interferometer. Now, due to the arms' one-meter vertical spacing, the ambient variables will be different for each arm. We should therefore expect each arm of the interferometer to be of different length. This difference in length will lead to an extra phase-shift that must be taken into account and that is derived directly from Equation (3.17). In Section 4 we proceed to derive expressions for σ _*xx*_ and σ _*yy*_ in terms of ambient pressure, temperature, and gravity, so that the differences *δ* p , *δ* *T*, and *δ*g of p , *T* and g indeed provide, via Equation (3.17), a formula for the change of the length of an arm after displacement.

Equation (3.17) is one of key results in this work, as it will allow us to determine the associated change of phase of photons exiting the waveguide. We expect this to be the dominant effect of elasticity on the phase in a perfectly isolated system. This expectation is confirmed in follow-up work [ 8], where the influence of all elastic deformations derived in this work on Maxwell fields is determined.

## 4 Contact models

Having formally solved the problem for arbitrary boundary conditions in the preceding section, we now implement the boundary conditions for the configurations shown in Figures 1.3a– 1.3d.

As already pointed out in the Introduction, the solutions for the configurations of Figures 1.3a– 1.3b, that we are about to derive, with the boundary conditions corresponding to contact lines, describe unphysical displacement fields, diverging at the contact interfaces. We show that this can be cured by deriving a solution involving extended contact regions, using the Hertz contact deformations formalism. We show that even though the displacements differ between these approaches, the stresses near the center of the waveguide are in reasonable agreement.

### 4.1 Line contacts

***4.1.1 Resting on a contact line*.** We start with the analysis of the simplest physical model, given by a cylindrical waveguide lying on an infinite plane, contacting (as a first approximation) only on a line. This is reminiscent of the famous Flamant solution with circular cross-section (see in particular [ 14, Problem 3 of Chapter 12]).

The boundary conditions are given by


$$\;\sigma_{r\theta }{\text{|}}_{\partial U}=0,\;(4.1)$$

$$\;\sigma_{rr}{\text{|}}_{\partial U}=\text{–}P\delta (\theta )-p,\;(4.2)$$

where P is the pressure with which the contact line is resisting the weight of the waveguide and the ambient pressure, taken to be constant. To put this into physical terms, we apply neither tangential nor radial forces, except for the contact line with the “contact wire”.

This model is particularly convenient, since the *δ*-distribution has a Fourier series expansion as


$$\;\delta (\theta )=\frac{1}{2\pi }\left[1+2\sum_{1\leq n}\cos(n\theta )\right].\;(4.3)$$

We find for the coefficients in the Michell solution


$$\;D_{0}=-\frac{P}{4\pi }-\frac{p}{2},\;(4.4)$$

$$\;C_{1}=0,\;(4.5)$$

$$\;A_{n}=\frac{P}{2\pi a^{n-2}(n-1)},\;(4.6)$$

$$\;C_{n}=-\frac{P}{2\pi a^{n}(n+1)},\;(4.7)$$

with


P=πagρ,(4.8)

as follows from the condition Equation (3.13).

This last constraint implements the physicality of the calculations, since in the absence of other forces there can only be an equilibrium configuration if the integrated body force of the waveguide


$$\int (-F_{y})r\;\text{d}r\text{d}\theta =\int \text{g}\rho \;r\;\text{d}r\text{d}\theta =\pi a^{2}\text{g}\rho \text{,}\;(4.9)$$

matches the reactive force exerted by the plane, given by


$$\int P\delta (\theta )r\;\text{d}\theta {|}_{\partial U}=-\pi a^{2}\text{g}\rho \text{.}\;(4.10)$$

The sum in ( 2.27) converges for coefficients ( 4.4)–( 4.7), yielding


$$\phi (r,\theta )=-\frac{1}{2}r^{2}p+\frac{1}{4}r\text{g}\rho \;\left[r(a+r\;\text{cos}(\theta ))-4a^{2}\arctan\left(\frac{r\;\text{sin}(\theta )}{a-r\;\text{cos}(\theta )}\right)\;\text{sin}(\theta )\right],\;(4.11)$$

and further for the stresses


$$\;\begin{array}{l} \sigma_{rr}=A\left[r(6a^{2}+r^{2})\text{cos}(\theta )-a\left(a^{2}+3r^{2}+2(a^{2}+r^{2})\text{cos}(2\theta )-ar\;\text{cos}(3\theta )\right)\right]-p,\;(4.12) \end{array}$$

$$\sigma_{r\theta }=A\left[4a(a^{2}+r^{2})\text{cos}(\theta )-r\left(5a^{2}+r^{2}+2a^{2}\text{cos}(2\theta )\right)\right]\text{sin}(\theta ),\;(4.13)$$

$$\;\begin{array}{l} \sigma_{\theta \theta }=A\left[-r(2a^{2}+r^{2})\text{cos}(\theta )+a\left(r^{2}-a^{2}+2(a^{2}+r^{2})\text{cos}(2\theta )-ar\;\text{cos}(3\theta )\right)\right]-p,\;(4.14) \end{array}$$

with


$$A\;\text{:=}\;\frac{\text{g}\rho (a^{2}-r^{2})}{2{\left[a^{2}+r^{2}-2ar\;\text{cos}(\theta )\right]}^{2}}.$$

**Figure 4.1.**
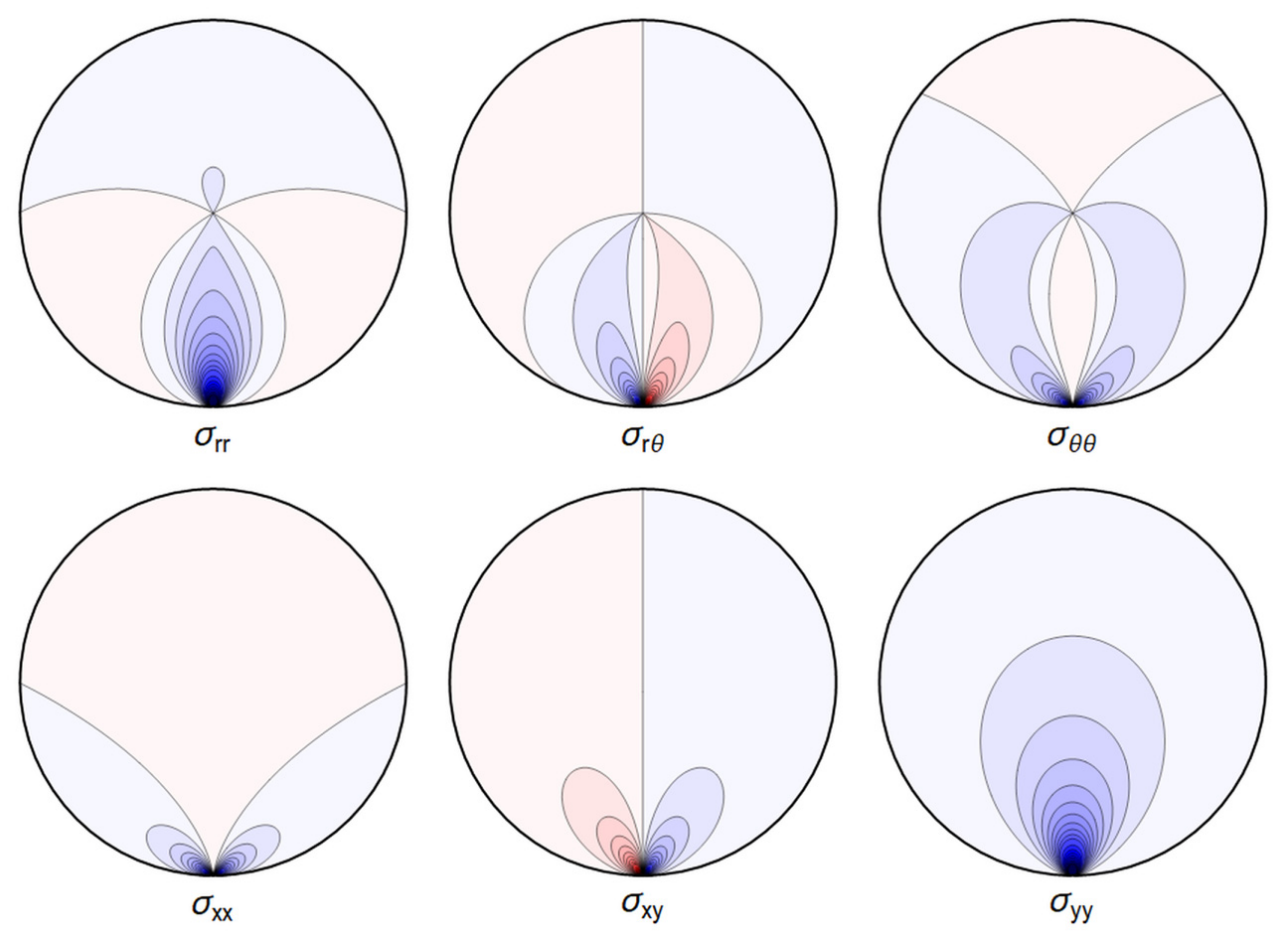
Typical plot of internal stresses for the case of a waveguide resting on an infinitely thin line, with unrealistic parameters arbitrarily chosen for illustration purposes listed in Table 4.1.

A representative plot of the stresses can be seen in Figure 4.1.

**Table 4.1.** Set of parameters chosen in our visualizations. For conciseness, we express lengths in multiples of *a*, and pressure in multiples of the shear modulus *μ*, leading to dimensionless quantities for the remaining parameters used in numerical calculations.

*ρ*g	*ν*	, *κ, α*	$\tilde{P}$	$\hat{P}$ = $\overset{\vee }{P}$
0.01	0.17	0	1	0.3

Here, and in all similar figures, shades of blue indicate compressive (negative) stresses, shades of red indicate positive ones, with darker colors encoding larger stresses.

**Figure 4.2.**
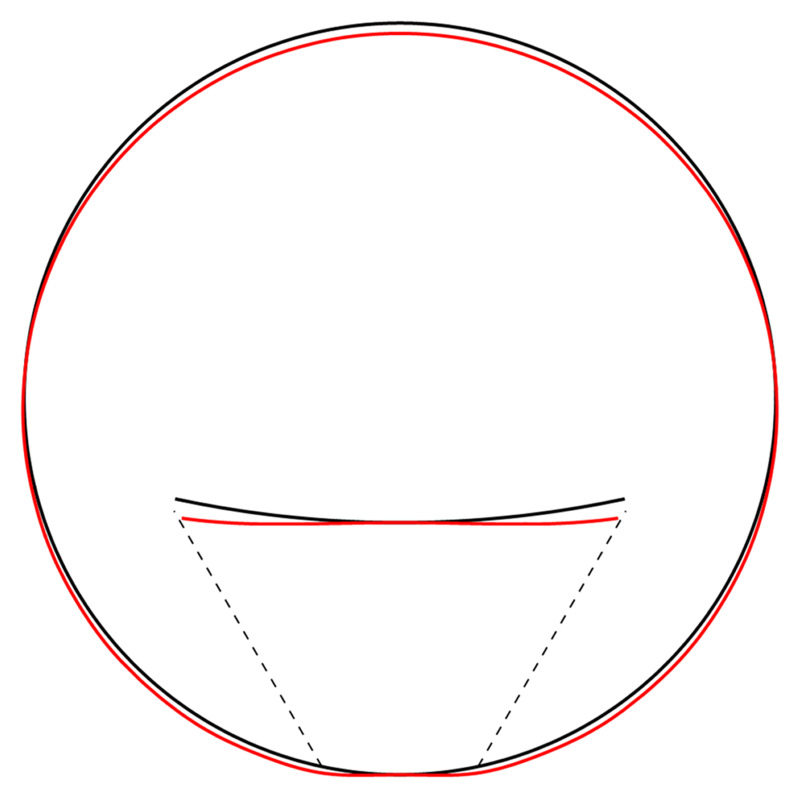
Illustrative deformation for the case of a waveguide resting on an infinitely thin line, after truncating the sums in ( 2.28)–( 2.30) to *n* = 20, drawn in red. The undeformed reference is drawn in black. Here and in similar figures below, the inset shows a magnified section around the contact point.

Equation (2.28)– Equation (2.30) do not make sense, as the sum in Ξ does not converge. But one can find explicit expressions for *u _r_* and *u _θ_* by integration; the resulting formulae are lengthy and not very enlightening, therefore we did not include them here. Not unexpectedly, and similar to the Flamant solution [ 9, Chapter 8.4.7] , the singularity in the function *A* at *r* = *a* and cos *θ* = 1 leads to an infinite displacement there. Hence the boundary condition *u*( *r* = *a, θ* = 0) = 0 cannot be imposed. However, the displacements obtained by direct integration are finite away from the contact point, in particular near the center of the waveguide (cf. [ 16]). One can also truncate the series ( 2.28)–( 2.29) which leads to finite solutions, illustrated in Figure 4.2.

***4.1.2 Squeezed between two lines*.** In a waveguide wound on a spool, consecutive layers of the waveguide press onto each other. The simplest model for this is a cylinder squeezed between two contact lines, with a pressure $\tilde{P}$ pushing from the top, as in Figure 1.3b. See also [ 9, Example 8–10] and [ 14, Problem 1 of Chapter 12], where a similar problem is modelled by superposition of three particular stress fields, including two Flamant solutions together with a uniform radial tension loading. The boundary conditions are given by


$$\;\sigma_{r\theta }{\text{|}}_{\partial U}=0,\;(4.15)$$

$$\;\sigma_{rr}{\text{|}}_{\partial U}=-P\delta (\theta )-\tilde{P}\delta (\theta -\pi )-p.\;(4.16)$$

The calculation is completely analogous to the above, with the boundary condition enforcing


$$\;D_{0}=-\frac{P+\tilde{P}}{4\pi }-\frac{p}{2},\;(4.17)$$

$$\;C_{1}=0,\;(4.18)$$

$$\;A_{n}=\frac{P+{\left(\text{−}1\right)}^{n}\tilde{P}}{2\pi a^{n-2}(n-1)},\;(4.19)$$

$$\;C_{n}=-\frac{P+{\left(\text{−}1\right)}^{n}\tilde{P}}{2\pi a^{n}(n+1)},\;(4.20)$$

with the force-balancing condition Equation (3.13) now reading


$$\;P=\tilde{P}+\pi a\text{g}\rho .\;(4.21)$$

Again, the physical interpretation is that now the force from above necessitates a reactive force from below larger than in ( 4.8) to achieve equilibrium.

**Figure 4.3.**
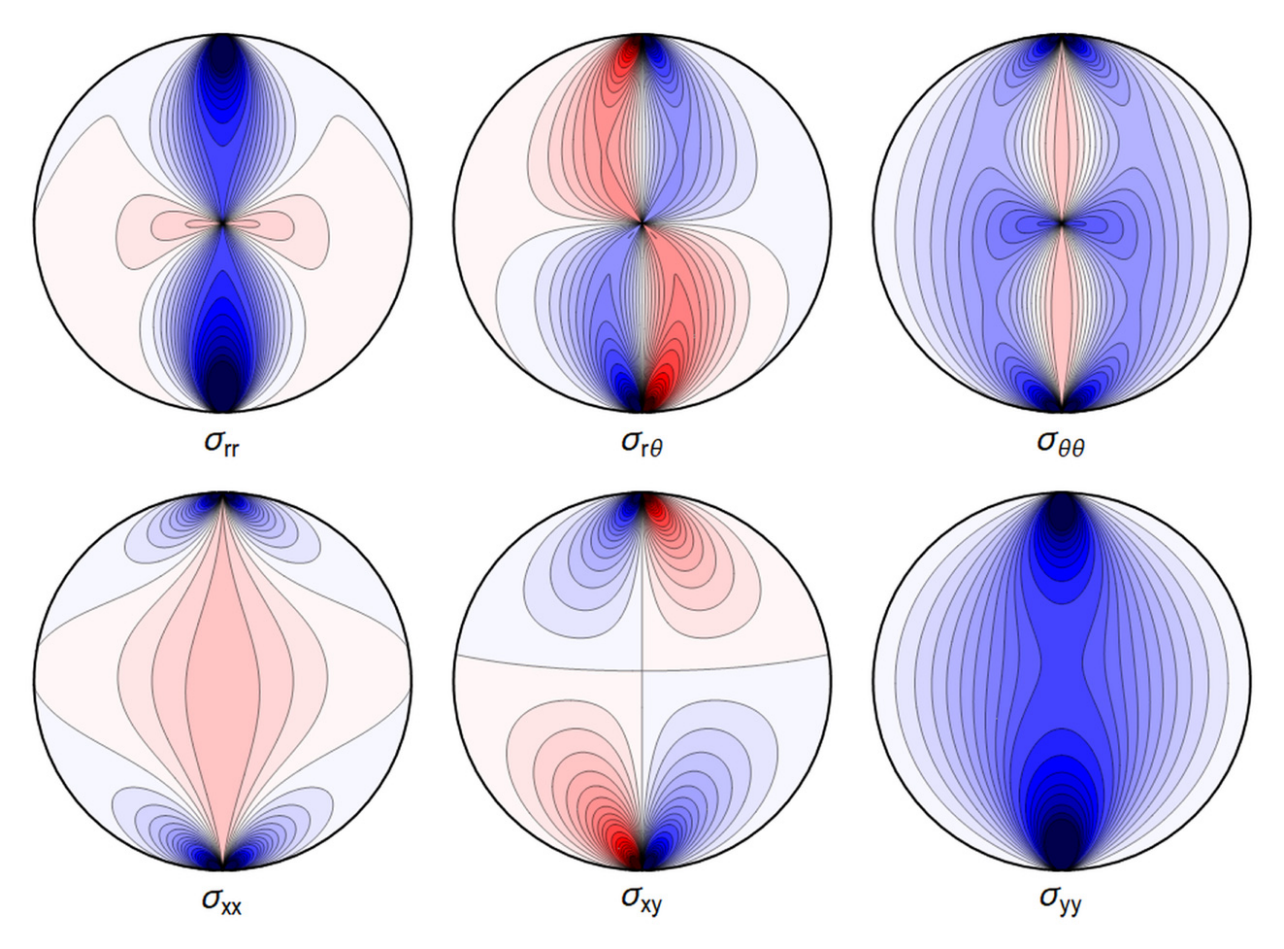
Typical plot of internal stresses for the case of a waveguide squeezed between two lines; same parameters and color coding as in Figure 4.1.

Denoting by $\sigma_{ij}^{I}$ the right-hand sides of ( 4.12)–( 4.14), the stress components read


$$\;\sigma_{rr}=\sigma_{rr}^{I}-B(a^{2}-r^{2})\left[a^{4}-2a^{2}r^{2}-r^{4}+2a^{4}\;\text{cos}(2\theta )\right],\;(4.22)$$

$$\;\sigma_{r\theta }=\sigma_{r\theta }^{I}+2Ba^{2}(a^{4}-r^{4})\text{sin}(2\theta ),\;(4.23)$$

$$\;\sigma_{\theta \theta }=\sigma_{\theta \theta }^{I}-B\left[a^{6}+5a^{4}r^{2}+a^{2}r^{4}+r^{6}-2a^{2}(a^{4}+a^{2}r^{2}+2r^{4})\;\text{cos}(2\theta )\right],\;(4.24)$$

with $B\;\text{:=}\;\frac{\tilde{P}}{\pi }(a^{2}-r^{2}){\left[a^{4}+r^{4}-2a^{2}r^{2}\;\text{cos}(2\theta )\right]}^{-2}$ . Again, visualizing the result gives a clear picture. See Figure 4.3.

**Figure 4.4.**
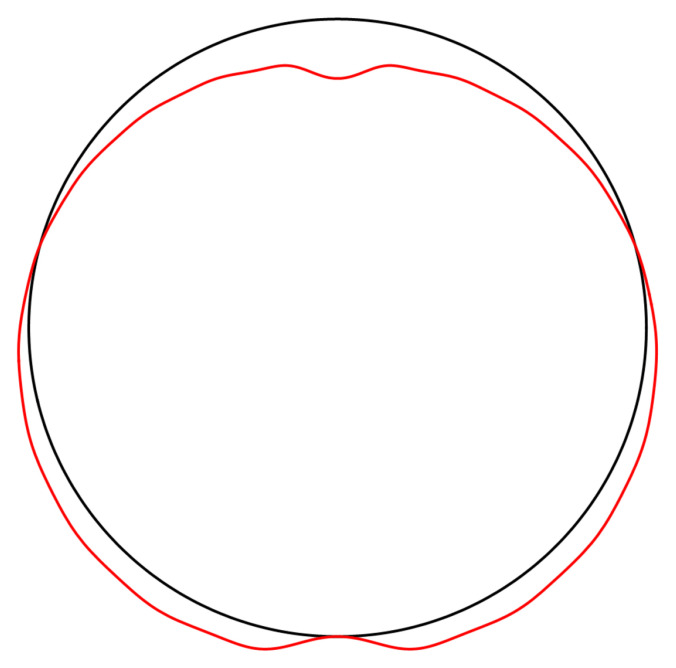
Illustrative deformation for the case of a waveguide squeezed between two lines, after truncating the sums in ( 2.28)–( 2.30) to *n* = 20, drawn in red. The undeformed reference is drawn in black.

As before, the deformation diverges at the “contact wires”. A truncated sum is shown in Figure 4.4.

### 4.2 Hertz contact deformations

In view of the above divergences, a better model for the contact between the waveguide and its support, or between neighbouring strands of the waveguide, is needed. A possible solution for this in linear elasticity is given by the Hertz contact deformation [ 17], with a modern derivation given by [ 10, Chapter 9].

To implement this we consider two cylinders contacting lengthwise. The contact region, which was previously described as a line, becomes a strip. On each cross-section, the contact point of the previous analysis becomes an interval (cf. [ 10, Problem 2 of Chapter 9]). The total force per unit length pushing two bodies into each other is now distributed over a region { *x* : *−s ≤ x ≤ s*}, where


$$\;s=\sqrt{\frac{4F}{\pi }\;\left(\frac{1-\nu^{2}}{E}+\frac{1-{\nu^{\prime }}^{2}}{E^{\prime }}\right)\;\frac{RR^{\prime }}{R+R^{\prime }}},\;(4.25)$$

with *{ν, E, R}* the material properties and radius of curvature of the body in question and { *ν′, E′, R′}* for the body in contact. In this model the pressure distribution over the contact region, which we denote by *P _y_*, equals


$$\;P_{y}=\frac{2F}{\pi s}\sqrt{1-\frac{x^{2}}{s^{2}}}.\;(4.26)$$

This can be restated in the form of boundary conditions


$$\;\sigma_{r\theta }\text{|}\partial U=0,\;(4.27)$$

$$\;\sigma_{rr}\text{|}\partial U=\{\begin{array}{ll} \frac{2F}{\pi a\;\text{tan}(\text{Θ})}\sqrt{1-\frac{{\text{tan}}^{2}(\theta )}{{\text{tan}}^{2}(\text{Θ})}} & -\text{Θ}\leq \theta \leq \text{Θ}\\ 0 & \text{otherwise},\;(4.28) \end{array}$$

where


$$\;\text{Θ}=\text{arctan}\left(\frac{s}{a}\right).\;(4.29)$$

For the case of an infinite cylinder resting on a rigid plane, we simply take *R′, E′ → ∞* and *ν′ →* 0.

**Figure 4.5.**
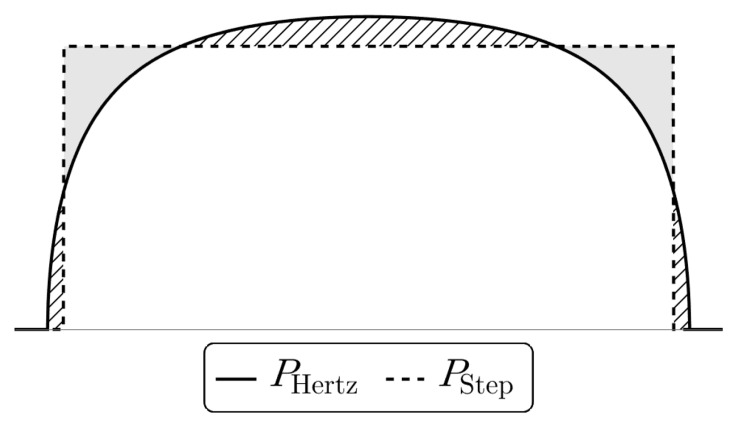
Comparison of the pressure distribution in the contact region for the exact Hertz solution ( 4.28) and an approximation using constant pressure in the contact region. The shaded and dashed regions cause overshoot and undershoot in the displacement figures below.

Finding the cosine expansion of ( 4.28) is not obvious. Instead, we will approximate the Hertz profile with a step-function as in Figure 4.5, with straightforward cosine expansion, allowing us to find explicit expressions for the Airy functions and stresses in the settings discussed below. The half-width of the step function can be determined, based on the Hertz solution, to


$$\;s_{\text{Hertz}}=\sqrt{\frac{4aF}{\pi }\frac{1-\nu^{2}}{E}},\;(4.30)$$

$$\;{\text{Θ}}_{\text{Hertz}}=\text{arctan}\left(\frac{s_{\text{Hertz}}}{a}\right),\;(4.31)$$

via ( 4.25) and ( 4.29), ensuring a physically motivated boundary pressure distribution in response to external forces.

For the purpose of the GRAVITES experiment the key role is played by a) the change of length of the waveguide and b) the deformations near the center of the waveguide, where the core of the waveguide is located and where the photons propagate, with the energy of the waves decaying exponentially fast in the cladding. In Figure 4.10 and Figure 4.11 below we show a typical plot of differences between the point-contact solution and our variation of the Hertz-type solution. The figures make it clear that the differences are subdominant in the region of interest. In Figure 4.12 we further show that the difference between the Hertz-type solution and the step-function model is negligible for all our purposes. Our calculations show that both the point-contact approximation and the step-function approximation provide a good approximation for weak forces and away from the contact region, with a numerical comparison provided in Section 4.2.3.

***4.2.1 Resting on a rigid plane*.** We apply the above to a waveguide resting on a plane, as in Figure 1.3a. Approximating the Hertz contact solution of Section 4.2 by a step-function of constant pressure P for a single contact region from below leads to the boundary conditions


$$\;\sigma_{r\theta }\text{|}\partial U=0,\;(4.32)$$

$$\;\sigma_{rr}\text{|}\partial U=-P_{\chi \left[-\Theta ,\Theta \right]}(\theta )-p(1-\chi_{\left[-\Theta ,\Theta \right]}(\theta ))=:-P^{\prime }\chi_{\left[-\Theta ,\Theta \right]}(\theta )-p,\;(4.33)$$

with Θ ≔ Θ _Hertz_ as defined in Equation (4.31), where for any set *Ω* we set


$$\;\chi_{\Omega }(x):=\{\begin{array}{ll} 1 & x\in \Omega \\ 0 & \text{else}.\;(4.34) \end{array}$$

By assumption our boundary conditions for *σ _rr_* are mirror-symmetric with respect to the vertical axis and 2π-periodic. We can write a general formula for the Fourier cosine expansion of their symmetrized sum. For ψ, Θ ∈ [0, π] we have,


$$\chi_{\left[\Psi -\Theta ,\Psi +\Theta \right]}\left(\text{θ}\right)+\chi_{\left[-\Psi -\Theta ,-\Psi +\Theta \right]}\left(\text{θ}\right)=\frac{2\text{Θ}}{\pi }+\sum_{n\geq 1}\frac{4\text{cos}\left(n\text{Ψ}\right)\text{sin}\left(n\text{Θ}\right)}{\text{π}n}\text{cos}\left(n\text{θ}\right).\;(4.35)$$

When ψ = 0 we obtain the following coefficients in ( 2.27):


$$\;D_{0}=-\frac{P^{\prime }\text{Θ}}{2\pi }-\frac{p}{2},\;(4.36)$$

$$\;C_{1}=0,\;(4.37)$$

$$\;A_{n}=\frac{P^{\prime }\text{sin}(n\text{Θ})}{\pi a^{n-2}n(n-1)},\;(4.38)$$

$$\;C_{n}=-\frac{P^{\prime }\text{sin}(n\text{Θ})}{\pi a^{n}n(n+1)},\;(4.39)$$

With condition Equation (3.13) reading


$$\;P^{\prime }=\frac{a\text{g}\rho \pi }{2\text{sin}(\text{Θ})}.\;(4.40)$$

The summed expression for the Airy function is


$$\begin{array}{l} \phi_{\text{single}}=\frac{1}{4}g\rho \left[\left(2a^{2}+r^{2})r\;\text{cos}(\theta )-ar^{2}\frac{\text{Θ}}{\text{sin}(\text{Θ})}+\frac{a}{\text{sin}(\text{Θ})}(\zeta (\theta -\text{Θ})-\zeta (\theta +\text{Θ})\right)\right]-\frac{1}{2}r^{2}p,\;(4.41) \end{array}$$

where the subscript “single” refers to a single contact region, with


$$\;\zeta (x):=\left[a^{2}+r^{2}-2ar\;\text{cos}(x)\right]\;\text{arctan}\;\left(\frac{r\;\text{sin}(x)}{a-r\;\text{cos}(x)}\right).\;(4.42)$$

**Figure 4.6.**
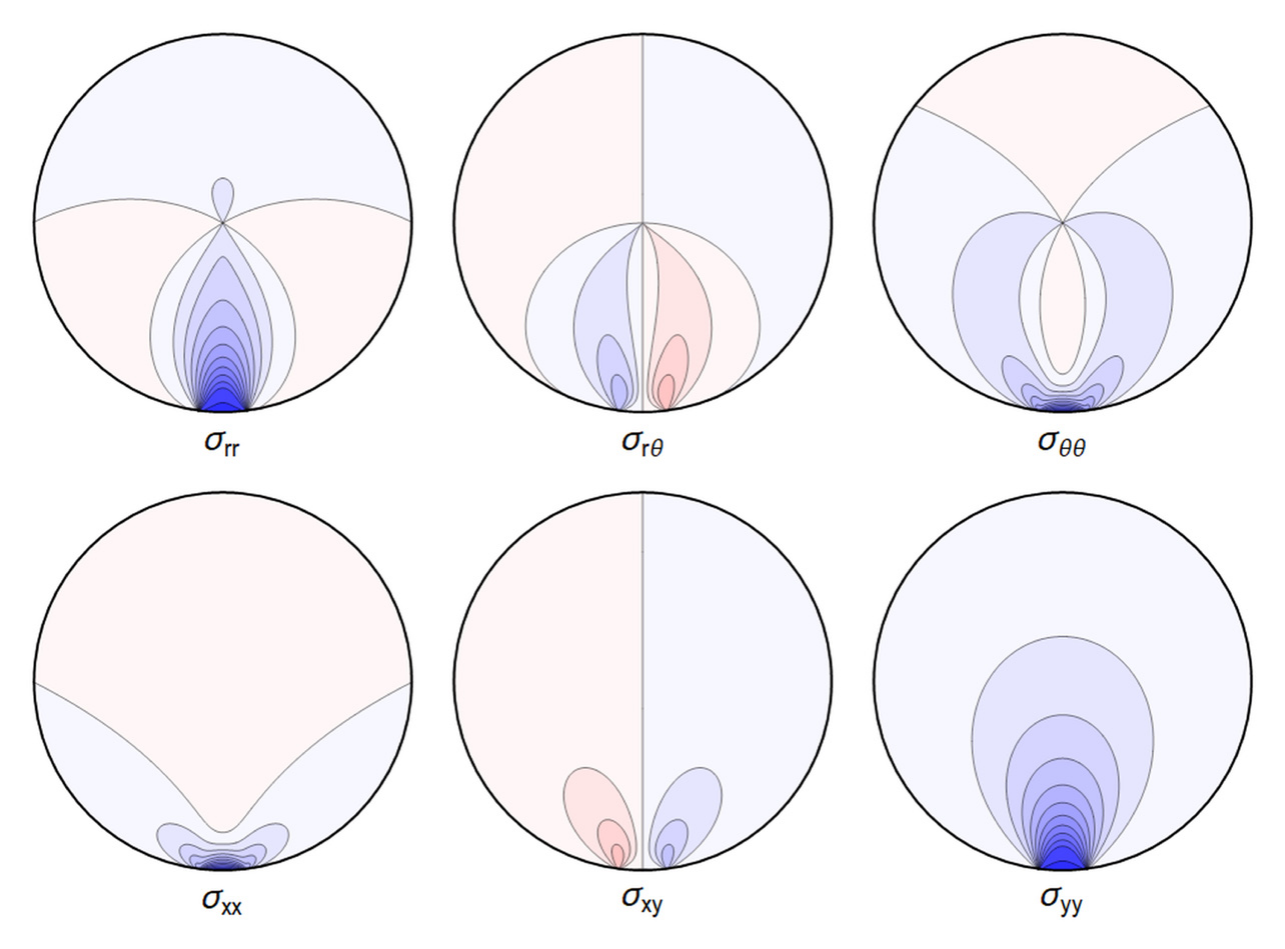
Typical internal stresses for a waveguide resting on an extended contact zone. Here, and in the following figures, we use the same parameters and the same color coding as in Figure 4.1.

**Figure 4.7.**
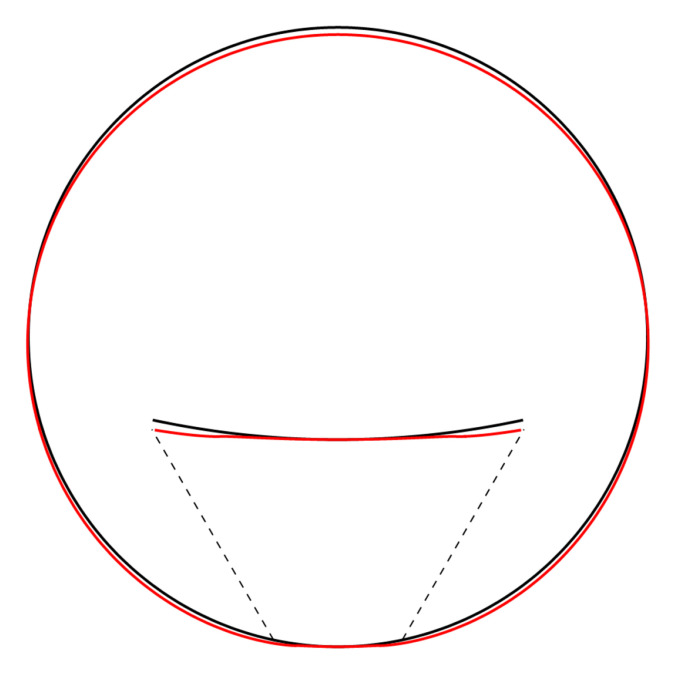
Deformation for the case of a waveguide resting on a rigid plane, drawn in red. The undeformed reference is drawn in black. Here, and in similar figures that follow, the (barely visible) indentation is an artefact of the approximation illustrated in Figure 4.5.

All the sums converge, leading to an admissible displacement field. The stresses and the displacements are shown in Figure 4.6 and Figure 4.7.

***4.2.2 Squeezed between two rigid planes*.** The next simplest description of a spooled waveguide is one with extended contact regions both above and below. We model this by squeezing the fiber between two rigid planes in the spirit of Section 4.2.1, i.e. boundary conditions


$$\sigma_{r\theta }\text{|}\partial U=0,\;(4.43)$$

$$\begin{array}{l} \sigma_{rr}\text{|}\partial U=-P_{\chi \left[-\Theta ,\Theta \right]}(\theta )-\tilde{P}\chi_{\left[\pi -\tilde{\Theta },\pi +\tilde{\Theta }\right]}(\theta )-p\left[1-\chi_{\left[-\Theta ,\Theta \right]}(\theta )-\chi_{\left[\pi -\tilde{\Theta },\pi +\tilde{\Theta }\right]}(\theta )\right]\\ \;=:\;-P^{\prime }\chi_{\left[-\Theta ,\Theta \right]}(\theta )-{\tilde{P}}^{\prime }\chi_{\left[\pi -\tilde{\Theta },\pi +\tilde{\Theta }\right]}(\theta )-p,\;(4.44) \end{array}$$

with Θ now an angle which approximates half of the contact region from below and $\tilde{\Theta }$ the same from above.

The Fourier coefficients for the Airy function are found to be


$$\;D_{0}=-\frac{P^{\prime }\text{Θ}+{\tilde{P}}^{\prime }\tilde{\text{Θ}}}{2\pi }-\frac{p}{2},\;(4.45)$$

$$\;C_{1}=0,\;(4.46)$$

$$\;A_{n}=\frac{P^{\prime }\;\text{sin}(n\text{Θ})+{\left(-1\right)}^{n}{\tilde{P}}^{\prime }\;\text{sin}(n\tilde{\text{Θ}})}{\pi a^{n-2}n(n-1)},\;(4.47)$$

$$\;C_{n}=-\frac{P^{\prime }\text{sin}(n\text{Θ})+{\left(-1\right)}^{n}{\tilde{P}}^{\prime }\;\text{sin}(n\tilde{\text{Θ}})}{\pi a^{n}n(n+1)},\;(4.48)$$

with condition Equation (3.13) giving


$$\;2\;\text{sin}(\text{Θ})P^{\prime }=2{\tilde{P}}^{\prime }\;\text{sin}(\tilde{\text{Θ}})+a\text{g}\rho \pi .\;(4.49)$$

The re-summed expression for the Airy function is


$$\begin{array}{l} \phi_{\text{double}}=\phi_{\text{single}}-\frac{{\tilde{P}}^{\prime }}{2\pi }[r^{2}\;\left(\tilde{\text{Θ}}+\text{Θ}\frac{\text{sin}(\tilde{\text{Θ}})}{\text{sin}(\text{Θ})}\right)+\xi (\theta -\tilde{\text{Θ}})-\xi (\theta +\tilde{\text{Θ}})-\frac{\text{sin}(\tilde{\text{Θ}})}{\text{sin}(\text{Θ})}\left(\zeta (\theta -\text{Θ})-\zeta (\theta +\text{Θ})\right)],\;(4.50) \end{array}$$

where


$$\;\xi (x)\;:=\left[a^{2}+r^{2}+2ar\;\text{cos}(x)\right]\;\text{arctan}\;\left(\frac{r\;\text{sin}(x)}{a+r\;\text{cos}(x)}\right).\;(4.51)$$

**Figure 4.8.**
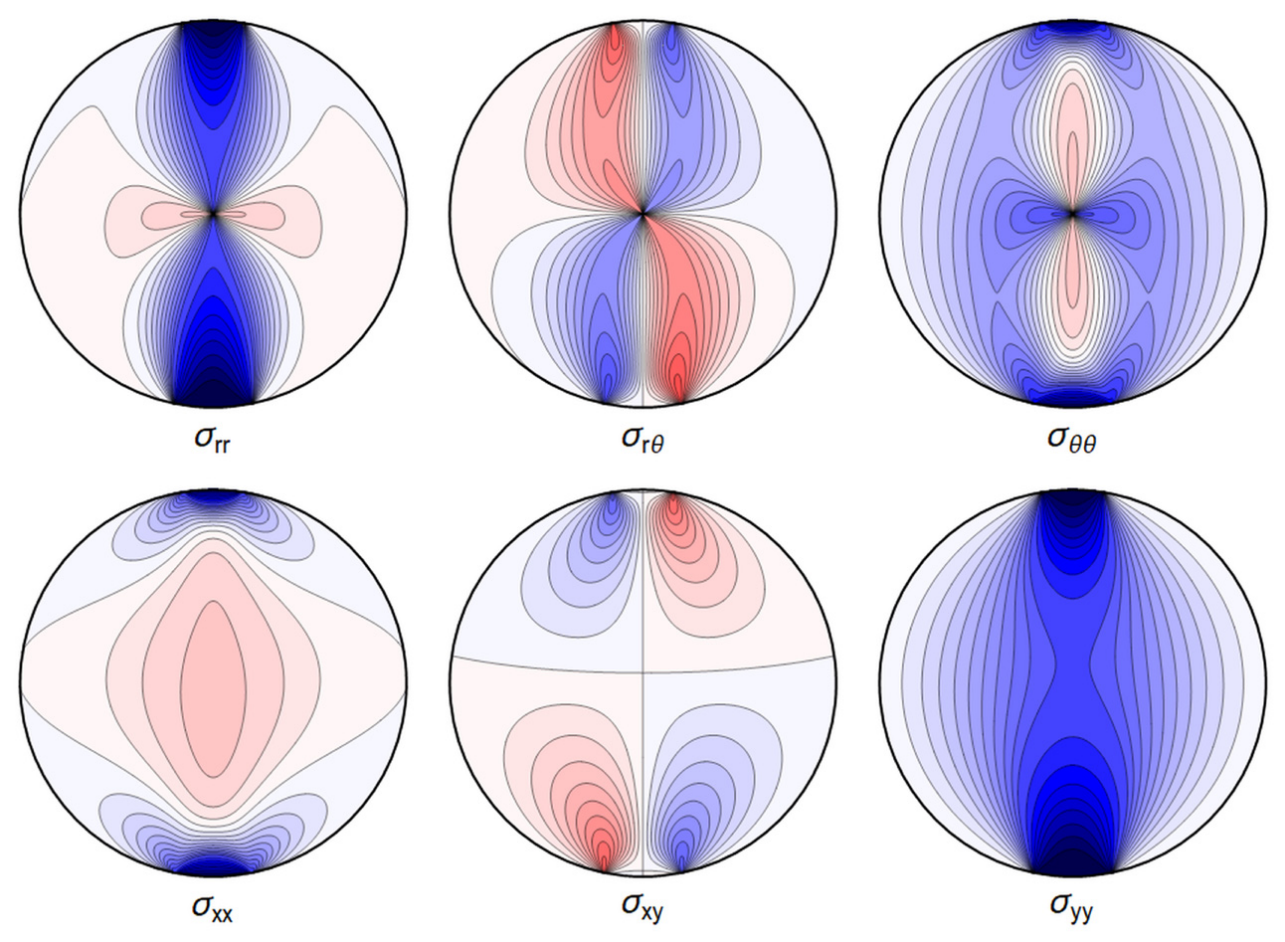
Internal stresses for the case of a waveguide squeezed between two rigid planes with extended contact region.

**Figure 4.9.**
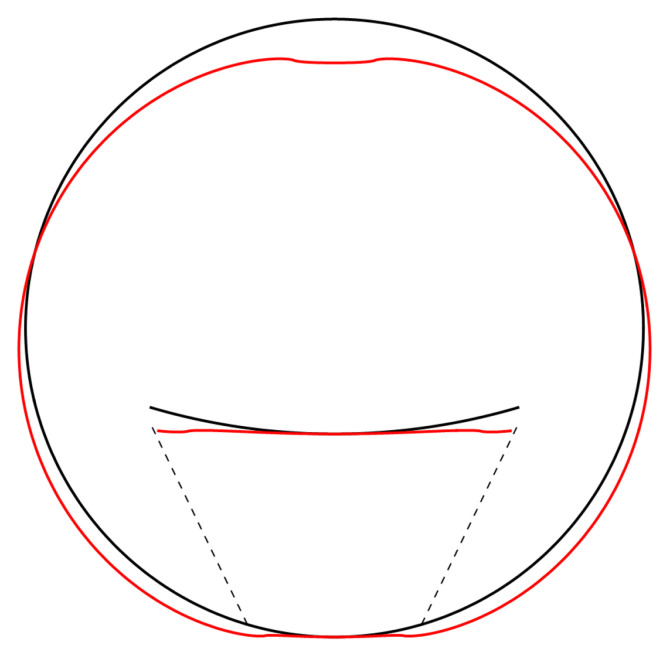
Deformation for the case of a waveguide squeezed between rigid planes of finite width, drawn in red. The undeformed reference is drawn in black.

All the sums converge, leading to a finite displacement field. The stresses and the displacements can be seen in Figure 4.8 and Figure 4.9 respectively. Here, and in the plots that follow, the parameters are related to those of the two contact-lines case via the correspondence


$$\;{\tilde{P}}_{\text{contact}\;\text{line}}={\tilde{P}}_{\text{extended}\;\text{contact}\;\text{region}}\times 2\tilde{\text{Θ}},\;(4.52)$$

which guarantees an identical integrated reaction force from the support.

**Figure 4.10.**
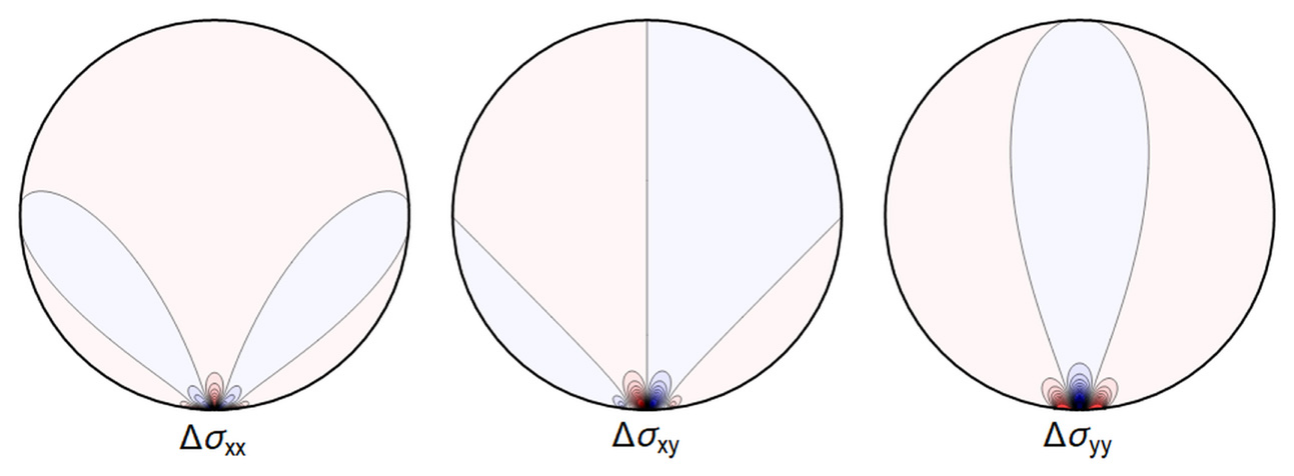
The difference ∆ _*σ*_ between the line contact Figure 4.1 and extended contact region Figure 4.6. Here and in all figures comparing two sets of stresses, shades of blue indicate negative differences, shades of red indicate positive ones, with darker colors encoding larger differences.

**Figure 4.11.**
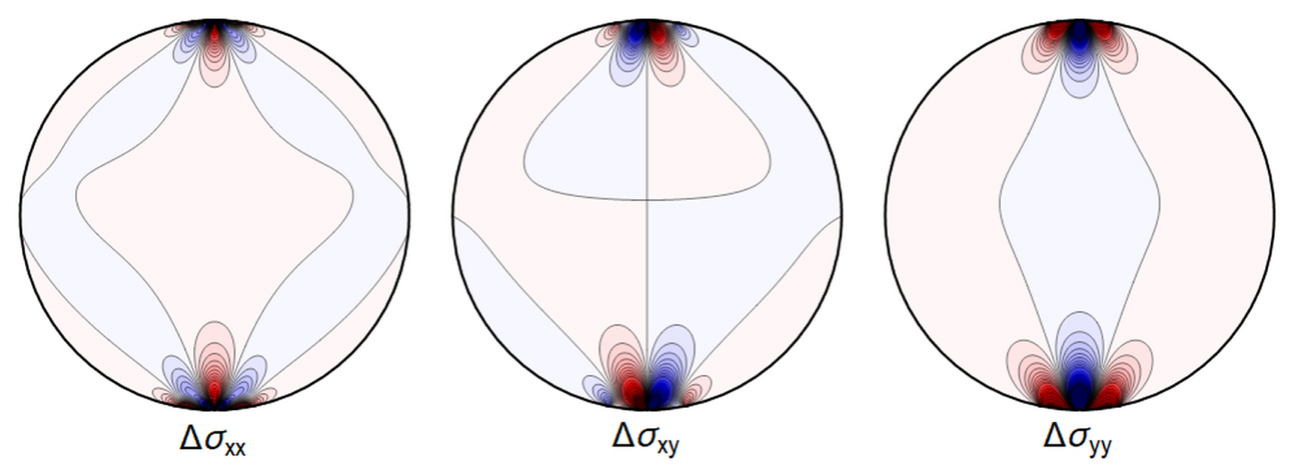
Difference ∆ *σ* between the line contact of Figure 4.3 and the extended contact region of Figure 4.8. (Same color coding as in Figure 4.10).

**Figure 4.12.**
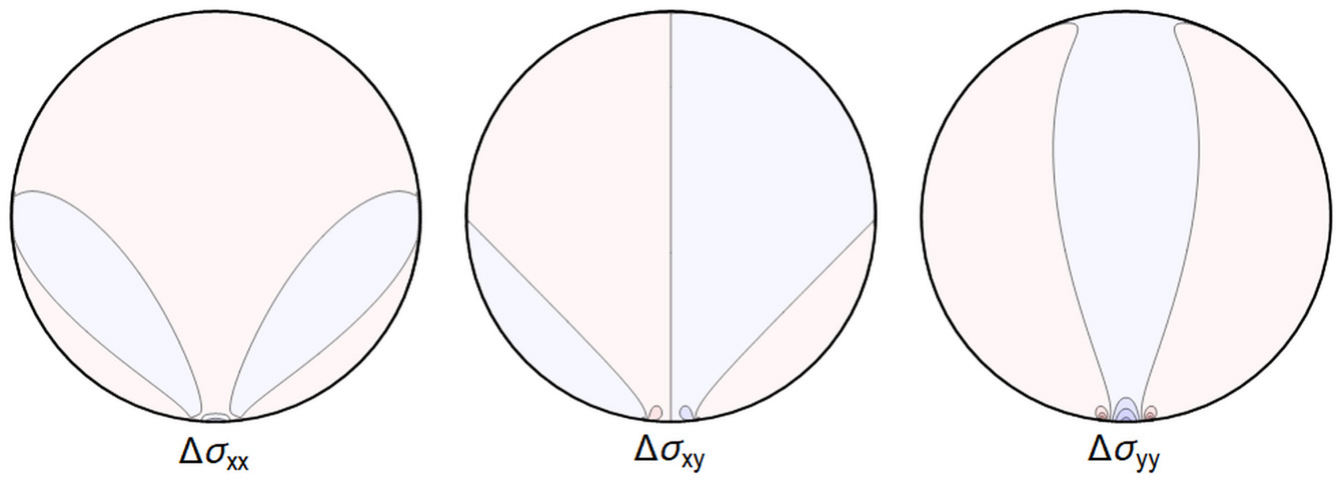
Difference ∆ *σ* between the extended contact of Figure 4.6 and the stresses given by the full Hertz contact solution. (Same color coding as in Figure 4.10 and Figure 4.11).

***4.2.3 Comparing models.*** It is of interest to compare the different contact models we employed above, in particular validating the assertion that the step-function is a good approximation for the Hertz contact pressure Equation (4.27)– Equation (4.28), at least far from the contact region. In Figure 4.10 and Figure 4.11 we show the differences between the line contact models as discussed in Section 4.1 and the step-function approximation employed in Section 4.2 going forward, for the set of parameters given in Table 4.1. In Figure 4.12 we show the difference between stresses resulting from the Hertz contact model, calculated numerically, and our explicit solution using the step-function approximation.

We can quantify the differences away from the contact points by comparing the maximal values over the regions


$$U_{1/2}=\{(r,\theta )\;:0<r\leq \frac{a}{2},-\pi <\theta \leq \pi \}\;(4.53)$$

and


$$\;U_{1/10}=\{(r,\theta )\;:0<r\leq \frac{a}{10},-\pi <\theta \leq \pi \}\text{,}\;(4.54)$$

and setting


$$\;{\text{Δ}}_{U}(\sigma_{1},\sigma_{2})=\frac{2\;{\text{max}}_{U}|\sigma_{1}-\sigma_{2}|}{{\text{max}}_{U}|\sigma_{1}|+{\text{max}}_{U}|\sigma_{2}|}.\;(4.55)$$

The results, for the parameters of Table 4.1 (used in all our figures), are shown in Table 4.2– Table 4.4. 

**Table 4.2.** Relative differences, as defined in Equation (4.55), between the point-contact model and our approximation of the Hertz-contact model for the model of Figure 1.3a.

	*σ _rr_*	*σ _rθ_*	*σ _θθ_*	*σ _xx_*	*σ _xy_*	*σ _yy_*
∆ _*U*_1/2__	2 *.*9%	4 *.*4%	5 *.*5%	11 *.*7%	6 *.*5%	2 *.*9%
∆ _*U*_1/10__	0 *.*6%	1 *.*1%	0 *.*9%	2 *.*5%	2 *.*3%	0 *.*6%

In the case of the line-contact model, a reasonable approximation is obtained in the central region for the values of parameters considered, if a 11% error is less than the measurement errors at hand. Comparing to the Hertz contact solutions, our step-function model gives errors of ~1% close to the center. Note in addition, that the chosen parameters for visualization correspond to much larger deformations than are expected to be relevant in physical applications, leading to more significant deviations.

**Table 4.3.** Relative differences, as defined in Equation (4.55), between the point-contact model and our approximation of the Hertz-contact model for the model of Figure 1.3b.

	*σ _rr_*	*σ _rθ_*	*σ _θθ_*	*σ _xx_*	*σ _xy_*	*σ _yy_*
∆ _*U*_1/2__	2.4%	9.9%	7.7%	11.0%	17.2%	3.9%
∆ _*U*_1/10__	1.4%	2.0%	1.7%	5.1%	10.4%	1.5%

**Table 4.4.** Relative differences, as defined in Equation (4.55), between the Hertz-contact model and our step-function approximation thereof.

	*σ _rr_*	*σ _rθ_*	*σ _θθ_*	*σ _xx_*	*σ _xy_*	*σ _yy_*
∆ _*U*_1/2__	0.7%	1.1%	1.4%	3.0%	1.6%	0.7%
∆ _*U*_1/10__	0.2%	0.3%	0.2%	0.6%	0.6%	0.2%

Of particular interest in this work is the effect on the length change given by Equation (3.17). The stress contributes to this change through the integral of its trace tr *σ* over the cross section of the body. In order to compare our models we evaluate the relative difference of the integrals over the cross-section of the trace of the stress tensor calculated in different models:


$$\;\Delta_{\int }\left(\sigma_{1},\sigma_{2}\right)≔\frac{2\left|\int_{U}\text{tr}\sigma_{\text{1}}-\int_{U}\text{tr}\sigma_{\text{2}}\right|}{\left|\int_{U}\text{tr}\sigma_{\text{1}}\right|+\left|\int_{U}\text{tr}\sigma_{\text{2}}\right|}.\;(4.56)$$

For the parameters listed in Table 4.1 this yields


$$\begin{array}{l} {\text{Δ}}_{\int }\left(\sigma_{\text{step}},\sigma_{\text{Hertz}}\right)=0.08\text{\%},\;{\text{Δ}}_{\int }\left(\sigma_{\text{line}},\sigma_{\text{Hertz}}\right)\;=0.20\text{\%,}\\ {\text{Δ}}_{\int }\left(\sigma_{\text{line}},\sigma_{\text{step}}\right)\;=0.27\text{\%},\;{\text{Δ}}_{\int }\left(\sigma_{\text{line}}^{\text{double}},\sigma_{\text{step}}^{\text{double}}\right)=0.21\text{\%}. \end{array}\;(4.57)$$

We conclude that replacing the Hertz-contact model by a step function gives an excellent approximation, and that our simplest model, namely a contact along a line, can also be used to obtain fairly accurate numbers, for the change of length and for the deformations near the core of the waveguide, in spite of the divergence of the displacement field *u* at the contact line, depending upon the resolution needed.

We emphasize that this is a comparison of different contact models for a waveguide at a given height, and should not be confused with formulae such as Equation (5.2) below, where two configurations at different heights are compared using the same model.

***4.2.4 Four contact regions*.** The calculations so far only take into account the neighbouring part of the waveguide pressing from above, ignoring the interaction between remaining neighbouring parts of the waveguide. The simplest model for this is presented in Figure 1.3c, with four contact regions for the waveguide, which we address now.

Using step-function boundary-stresses for the contact region gives


$$\sigma_{r\theta }\text{|}\partial U=0,\;(4.58)$$

$$\begin{array}{l} \sigma_{rr}\text{|}\partial U=-P_{\chi \left[-\text{Θ},\text{Θ}\right]}(\theta )-\tilde{P}\chi_{\left[\pi -\tilde{\text{Θ}},\pi +\tilde{\text{Θ}}\right]}(\theta )-\bar{P}\left[\chi_{\left[\frac{\pi }{2}-\hat{\text{Θ}},\frac{\pi }{2}+\hat{\text{Θ}}\right]}(\theta )+\chi_{\left[\frac{3\pi }{2}-\hat{\text{Θ}},\frac{3\pi }{2}+\hat{\text{Θ}}\right]}(\theta )\right]\\ \;-p\left[1-\chi_{\left[-\text{Θ},\text{Θ}\right]}(\theta )-\chi_{\left[\pi -\tilde{\text{Θ}},\pi +\tilde{\text{Θ}}\right]}(\theta )-\chi_{\left[\frac{\pi }{2}-\hat{\text{Θ}},\frac{\pi }{2}+\hat{\text{Θ}}\right]}(\theta )-\chi_{\left[\frac{3\pi }{2}-\hat{\text{Θ}},\frac{3\pi }{2}+\hat{\text{Θ}}\right]}(\theta )\right]\\ \;=:-P^{\prime }\chi_{\left[-\text{Θ},\text{Θ}\right]}(\theta )-{\tilde{P}}^{\prime }\chi_{\left[\pi -\tilde{\text{Θ}},\pi +\tilde{\text{Θ}}\right]}(\theta )-{\bar{P}}^{\prime }\left[\chi_{\left[\frac{\pi }{2}-\hat{\text{Θ}},\frac{\pi }{2}+\hat{\text{Θ}}\right]}(\theta )+\chi_{\left[\frac{3\pi }{2}-\hat{\text{Θ}},\frac{3\pi }{2}+\hat{\text{Θ}}\right]}(\theta )\right]-p,\;(4.59) \end{array}$$

with Θ now an angle which approximates half of the contact region from below and $\tilde{\Theta }$ the same from above and $\hat{\text{Θ}}$ from the sides.

The resulting Fourier coefficients in the Airy function are 


$$\;D_{0}=-\frac{P^{\prime }\text{Θ}+{\tilde{P}}^{\prime }\tilde{\text{Θ}}+2{\tilde{P}}^{\prime }\hat{\text{Θ}}}{2\pi }-\frac{p}{2},\;(4.60)$$

$$\;C_{1}=0,\;(4.61)$$

$$\;A_{n}=\frac{P^{\prime }\;\text{sin}(n\text{Θ})+{\left(-1\right)}^{n}{\tilde{P}}^{\prime }\;\text{sin}(n\tilde{\text{Θ}})+2{\bar{P}}^{\prime }\;\text{cos}\left(\frac{n\pi }{2}\right)\;\text{sin}(n\hat{\text{Θ}})}{\pi a^{n-2}n(n-1)},\;(4.62)$$

$$\;C_{n}=-\frac{P^{\prime }\;\text{sin}(n\text{Θ})+{\left(-1\right)}^{n}{\tilde{P}}^{\prime }\;\text{sin}(n\tilde{\text{Θ}})+2{\bar{P}}^{\prime }\;\text{cos}\left(\frac{n\pi }{2}\right)\;\text{sin}(n\hat{\text{Θ}})}{\pi a^{n}n(n+1)},\;(4.63)$$

with 


$$\;P^{\prime }={\tilde{P}}^{\prime }\frac{\text{sin}(\tilde{\text{Θ}})}{\text{sin}(\text{Θ})}+\frac{a\text{g}\rho \pi }{2\;\text{sin}(\text{Θ})},\;(4.64)$$

as by Equation (3.13). Note in particular the absence of a term proportional to $\bar{P}$ as evident by comparing to the *n* = 1-term in Equation (4.35). This can be physically interpreted as the sideways pressures not contributing to the force balance in the vertical direction.

**Figure 4.13.**
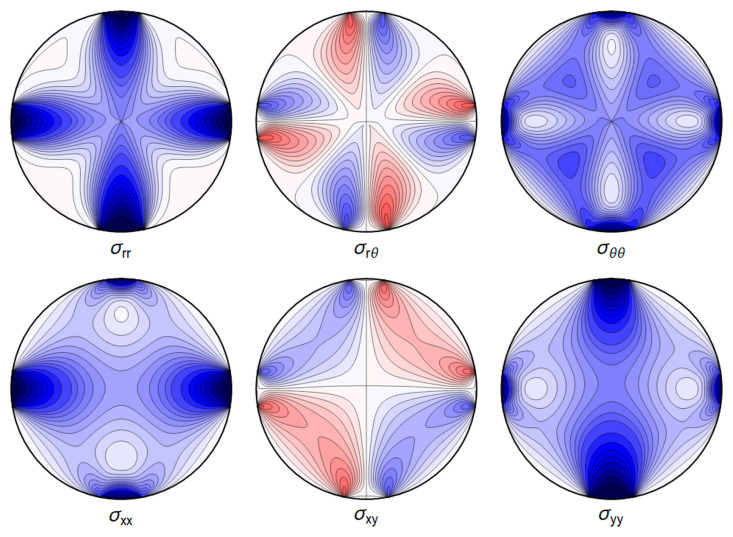
Internal stresses for the case of a waveguide squeezed between four rigid planes from below, above and the sides as depicted in Figure 1.3c, with extended contact region.

**Figure 4.14.**
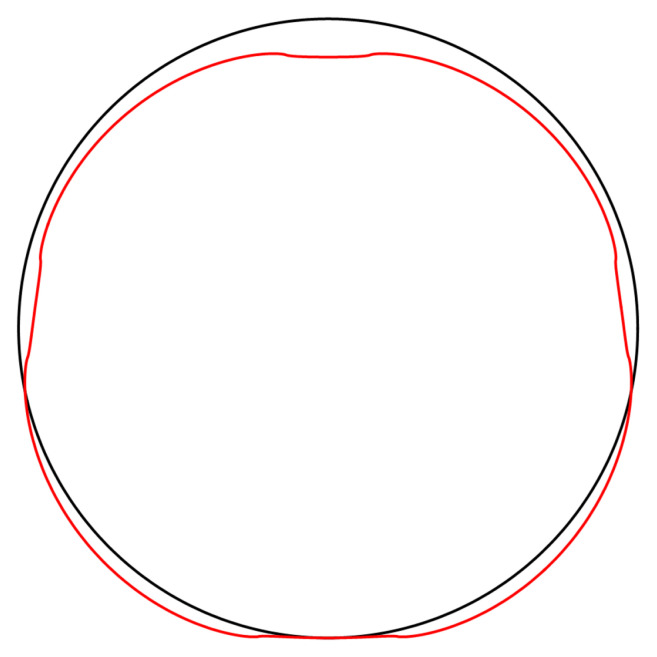
Deformation for the case of a waveguide squeezed between rigid planes of finite width as in Figure 1.3c, drawn in red. The undeformed reference is drawn in black.

All the sums converge, leading to an admissible displacement field. The stresses and the displacement can again be illustrated graphically – see Figure 4.13 and Figure 4.14.

***4.2.5 Squeezed between six rigid planes*.** Our final model for spooled waveguides is given by Figure 1.3d, which we model by imposing the boundary conditions


$$\;\sigma_{r\theta }\text{|}\partial U=0,\;(4.65)$$

$$\;\begin{array}{l} \sigma_{rr}\text{|}\partial U=-P_{\chi \left[-\text{Θ},\text{Θ}\right]}(\theta )-\tilde{P}\chi_{\left[\pi -\tilde{\text{Θ}},\pi +\tilde{\text{Θ}}\right]}(\theta )-\hat{P}\left[\chi_{\left[\frac{\pi }{3}-\hat{\text{Θ}},\frac{\pi }{3}+\hat{\text{Θ}}\right]}(\theta )+\chi_{\left[\frac{5\pi }{3}-\hat{\text{Θ}},\frac{5\pi }{3}+\hat{\text{Θ}}\right]}(\theta )\right]\\ \;–\overset{\vee }{P}\left[\chi_{\left[\frac{2\pi }{3}-\overset{\vee }{\text{Θ}},\frac{2\pi }{3}+\overset{\vee }{\text{Θ}}\right]}(\theta )+\chi_{\left[\frac{4\pi }{3}-\overset{\vee }{\text{Θ}},\frac{4\pi }{3}+\overset{\vee }{\text{Θ}}\right]}(\theta )\right]\\ \;-p[1-\chi_{\left[-\text{Θ},\text{Θ}\right]}(\theta )-\chi_{\left[\pi -\tilde{\text{Θ}},\pi +\tilde{\text{Θ}}\right]}(\theta )-\chi_{\left[\frac{\pi }{3}-\hat{\text{Θ}},\frac{\pi }{3}+\hat{\text{Θ}}\right]}(\theta )-\chi_{\left[\frac{5\pi }{3}-\hat{\text{Θ}},\frac{5\pi }{3}+\hat{\text{Θ}}\right]}(\theta )\\ \;-\chi_{\left[\frac{2\pi }{3}-\overset{\vee }{\text{Θ}},\frac{2\pi }{3}+\overset{\vee }{\text{Θ}}\right]}(\theta )-\chi_{\left[\frac{4\pi }{3}-\overset{\vee }{\text{Θ}},\frac{4\pi }{3}+\overset{\vee }{\text{Θ}}\right]}(\theta )]\\ \;=-P^{\prime }\chi_{\left[-\text{Θ},\text{Θ}\right]}(\theta )-\tilde{P}\prime \chi_{\left[\pi -\tilde{\text{Θ}},\pi +\tilde{\text{Θ}}\right]}(\theta )-\hat{P}\prime \left[\chi_{\left[\frac{\pi }{3}-\hat{\text{Θ}},\frac{\pi }{3}+\hat{\text{Θ}}\right]}(\theta )-\chi_{\left[\frac{5\pi }{3}-\hat{\text{Θ}},\frac{5\pi }{3}+\hat{\text{Θ}}\right]}(\theta )\right]\\ \;-\overset{\vee }{P^{\prime }}\left[\chi_{\left[\frac{2\pi }{3}-\overset{\vee }{\text{Θ}},\frac{2\pi }{3}+\overset{\vee }{\text{Θ}}\right]}(\theta )+\chi_{\left[\frac{4\pi }{3}-\overset{\vee }{\text{Θ}},\frac{4\pi }{3}+\overset{\vee }{\text{Θ}}\right]}(\theta )\right]-p,\;(4.66) \end{array}$$

with Θ now an angle which approximates half of the contact region from below, and $\tilde{\Theta }$ the same from above, and $\hat{\text{Θ}}$ and $\overset{\vee }{\text{Θ}}$ from the sides.

The associated Fourier cosine series for the Airy functions has coefficients


$$D_{0}=-\frac{P^{\prime }\text{Θ}+{\tilde{P}}^{\prime }\tilde{\text{Θ}}+2{\hat{P}}^{\prime }\hat{\text{Θ}}+2\overset{\vee }{P^{\prime }}\overset{\vee }{\text{Θ}}}{2\pi }-\frac{p}{2},\;(4.67)$$

$$C_{1}=0,\;(4.68)$$

$$\;A_{n}=\frac{1}{\pi a^{n-2}n(n-1)}\left[P^{\prime }\;\text{sin}(n\text{Θ})+{\left(\text{−}1\right)}^{n}{\tilde{P}}^{\prime }\;\text{sin}(n\tilde{\text{Θ}})+2{\hat{P}}^{\prime }\;\text{cos}\left(\frac{n\pi }{3}\right)\;\text{sin}(n\hat{\text{Θ}})+\overset{\vee }{2P^{\prime }}\text{cos}\left(\frac{2n\pi }{3}\right)\;\text{sin}(n\overset{\vee }{\text{Θ}})\right],\;(4.69)$$

$$\;C_{n}=-\frac{1}{\pi a^{n}n(n+1)}\left[P^{\prime }\;\text{sin}(n\text{Θ})+{\left(\text{−}1\right)}^{n}{\tilde{P}}^{\prime }\;\text{sin}(n\tilde{\text{Θ}})+2{\hat{P}}^{\prime }\;\text{cos}\left(\frac{n\pi }{3}\right)\;\text{sin}(n\hat{\text{Θ}})+\overset{\vee }{2P^{\prime }}\text{cos}\left(\frac{2n\pi }{3}\right)\;\text{sin}(n\overset{\vee }{\text{Θ}}\right)],\;(4.70)$$

with the force-balance condition Equation (3.13) leading to


$$\begin{array}{l} -a\text{g}\rho =-\frac{2}{\text{π}}\left(\tilde{P^{\prime }}\text{cos}\left(\text{π}\right)\text{sin}\left(\tilde{\text{Θ}}\right)+2\hat{P^{\prime }}\text{cos}\left(\frac{\text{π}}{\text{3}}\right)\text{sin}\left(\hat{\text{Θ}}\right)+2\overset{⌣}{P^{\prime }}\text{cos}\left(\frac{2\text{π}}{\text{3}}\right)\text{sin}\left(\overset{⌣}{\text{Θ}}\right)+P^{\prime }\text{cos}\left(0\right)\text{sin}\left(\text{Θ}\right)\right)\\ \;=\frac{2}{\text{π}}\left(\tilde{P^{\prime }}\text{sin}\left(\tilde{\text{Θ}}\right)-\hat{P^{\prime }}\text{sin}\left(\hat{\text{Θ}}\right)+\overset{⌣}{P^{\prime }}\text{sin}\left(\overset{⌣}{\text{Θ}}\right)-P^{\prime }\text{sin}\left(\text{Θ}\right)\right);\;(4.71) \end{array}$$

equivalently


$$\;P^{\prime }={\tilde{P}}^{\prime }\;\frac{\text{sin}(\tilde{\text{Θ}})}{\text{sin}(\text{Θ})}-{\hat{P}}^{\prime }\;\frac{\text{sin}(\hat{\text{Θ}})}{\text{sin}(\text{Θ})}+\overset{\vee }{P^{\prime }}\frac{\text{sin}(\overset{\vee }{\text{Θ}})}{\text{sin}(\text{Θ})}+\frac{a\text{g}\rho \pi }{2\;\text{sin}(\text{Θ})}.\;(4.72)$$

**Figure 4.15.**
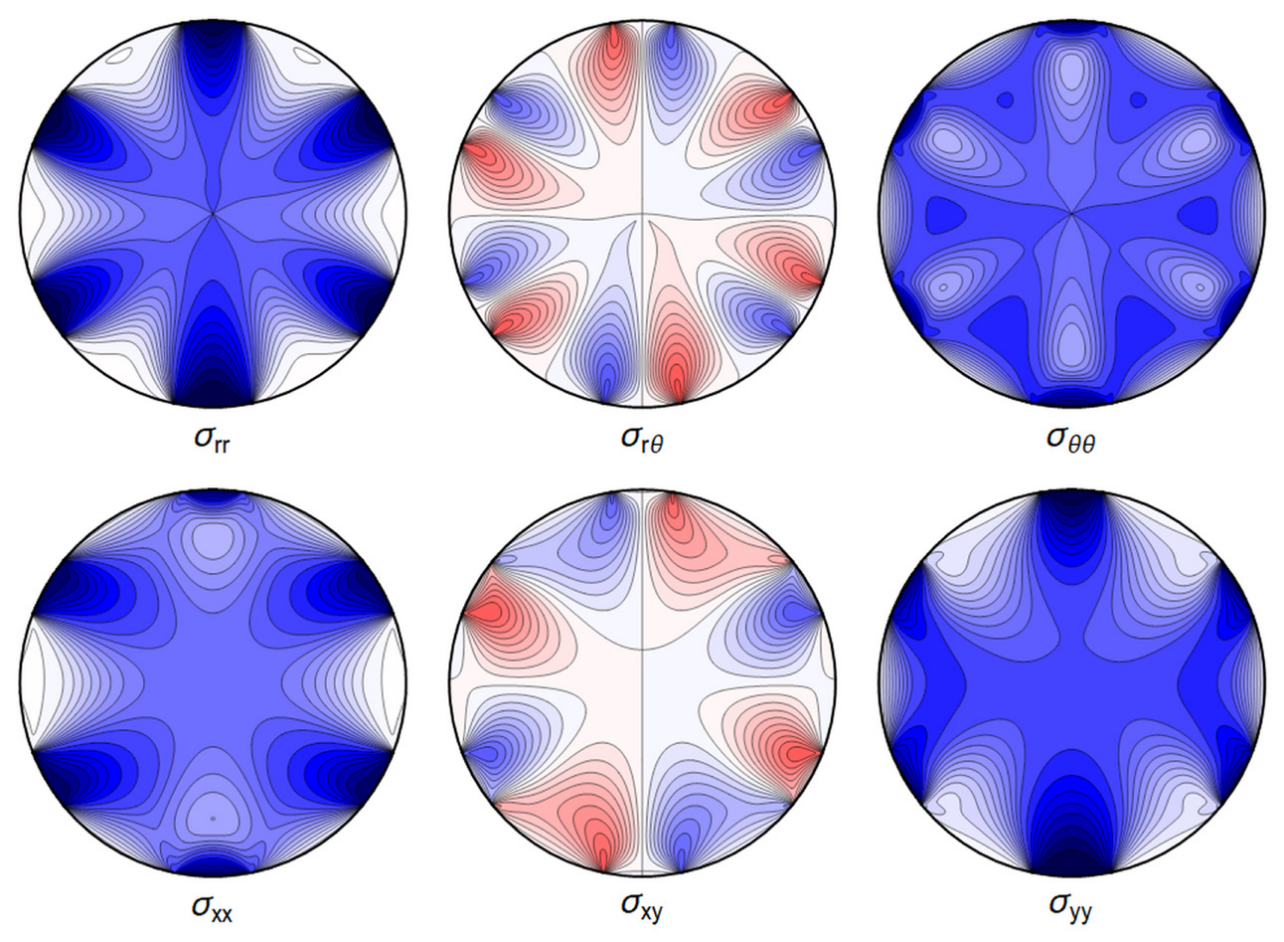
Internal stresses for the case of a waveguide squeezed between six rigid planes with extended contact regions, as in Figure 1.3d.

**Figure 4.16.**
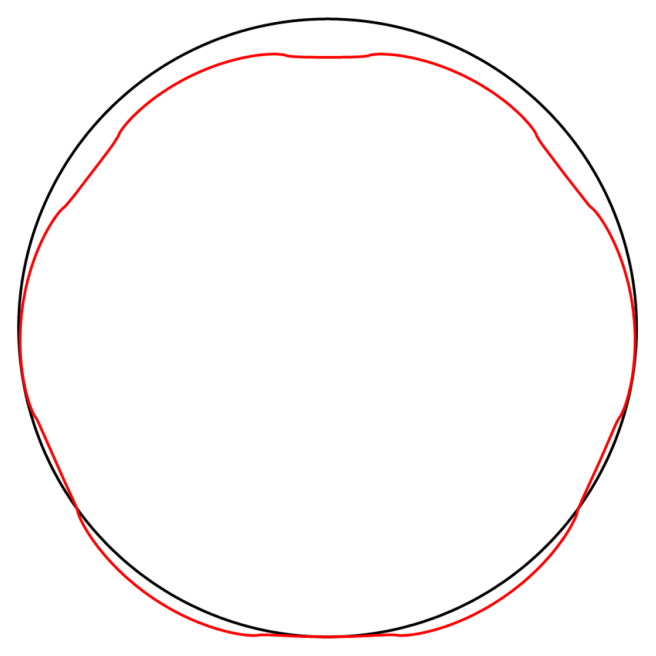
Deformation for the case of a waveguide squeezed between rigid planes of finite width as in Figure 1.3d, drawn in red. The undeformed reference is drawn in black.

All the sums converge, leading to a finite displacement field. The stresses and the displacement are seen in Figure 4.15 and Figure 4.16.

## 5 Estimates for GRAVITES

The planned GRAVITES experiment [ 1] will use single-mode optical fibers made of silica glass, which we assume to be homogeneous, with material properties and experimental parameters given in Table 5.1– Table 5.2. The numbers used below, such as the change of pressure *δ* and the change of temperature *δT*, and their outcome for the experiment, correspond to a change in height of 1 m at sea level for an unshielded experiment.

**Table 5.1.** Properties of silica oxide glass from [ 18].

*ν*	*E*	*ρ*	*α*
0.17	73.1 GPa	2.2 g/cm ^3^	1.8 · 10 ^−7^ K ^−1^

**Table 5.2.** Parameters for GRAVITES from [ 1].

*a*	*δ*g	*δp*	*δT*	*L*	*β*
62.5 µm	3 µm/s ^2^	10 Pa	10 ^–2^ K	10 ^5^ m	6 · 10 ^6^ m ^–1^

The results of our calculations make it clear that the experiment must be carried-out in a vacuum chamber, keeping in mind the following:

1. The effects scale in an obvious way with the residual pressure, in which case our calculations provide bounds on the quality of the vacuum needed for measurability of the gravitational effect.
2. The gradients of the temperature of the residual gas affect the temperature of the waveguide during the timescale of the experiment, in which case our calculations provide bounds on the residual temperature gradients.

The above can of course be circumvented by ensuring that there are neither pressure gradients nor temperature gradients, of the kind considered here, inside the vacuum chamber at the timescale of the experiment, so that the effects determined here become irrelevant. Our calculations give severe constraints on the experimental setup. It is currently planned to achieve a stability of temperature of ±20 mK at the locations of the spools, and an ambient pressure of 10 ^–1^ mbar for the duration of the measurement (as we cannot let the vacuum pumps run in view of the associated vibrations, and rely therefore on slow outgassing of the valved-off chamber). This means that the arms-stabilisation techniques will need to mitigate the effect of temperature changes to a level below the gravitational signal, taking into account the time scales associated with thermoelastic deformations of the waveguide.

In what follows we will need an estimate for the spooling force. The typical values for the equipment used are in the range from 0.2 to about 1 Newtons, with an accuracy of 0.05 N. We choose, in both arms,


$$F_{T}=1N,$$

and we assume that this is unchanged when moving the spools in the gravitational field.

In a first very rough estimate the lateral pressures $\hat{P}$ and $\overset{\vee }{P}$ , for the hexagonal case, are taken to be the same as $\bar{P}$ from the quadrilateral case, as given in ( A.2).

Finally, the maximal pressure $\tilde{P}$ for fibers at the bottom can be estimated by the weight of the spooled fiber *~* 10 kg distributed over the area of a horizontal cross-section of the spool, resulting in


$${\tilde{P}}_{\text{max}}=1.3\text{kPa}.$$

The actual value of $\tilde{P}$ decreases with height along the spool, vanishing for the top layer.

 For the following numeric estimates we use this maximal value.

In the GRAVITES experiment one interferes two photons traveling in two fibers with a typical vertical separation of 1 m. The arms of the interferometers are moved vertically during the experiment, which results in a deformation of the fiber due to a different gravitational field. The quantities that influence the measurements are the changes of deformations. Denoting by *δ* a change in a field, and by a bar the operation of integral averaging, we define the integral average $\delta {\bar{\sigma }}_{rr}$ of the difference of *σ _rr_* as,


$$\;\delta {\bar{\sigma }}_{rr}:=\frac{1}{\pi a^{2}}\int_{U}\left(\sigma_{rr}(r,\theta ){|}_{1\;\text{m}}-\sigma_{rr}(r,\theta ){|}_{0\;\text{m}}\right)r\;\text{d}r\;\text{d}\theta ,\;(5.1)$$

where the notation ⋅| *_h_* indicates that the quantity ⋅ is evaluated at a height *h*; analogously for $\delta {\bar{\sigma }}_{\theta \theta }$ . We use Equation (3.17) to determine the change in the length of the fiber,


$$\;\delta L=L\left[-\frac{\nu }{E}(\delta {\bar{\sigma }}_{rr}+\delta {\bar{\sigma }}_{\theta \theta })+\alpha T\right],\;(5.2)$$

and we use


δφ=βδL(5.3)

to denote the change of phase of light due to the change of length of the waveguide.

Consider the deformation vector


$$\text{δ}u\text{:}=u{\text{|}}_{1\text{m}}-u{\text{|}}_{0\text{m}}.$$

We wish to associate to it a number which will provide information about the magnitude of the deformation, and will allow us to compare the deformation for all our models. Taking the supremum over the waveguide is not useful, since *δu* is infinite for the line contact models. For definiteness, and as an example, we take the supremum of the length || *δu*|| over the region *U*_1/2_ of Equation (4.53),


$$\;{\left\|\delta u\right\|}_{\text{max}}≔\underset{U_{1/2}}{sup}\left\|\delta u\right\|,\;(5.4)$$

keeping in mind that the effects on light for GRAVITES are mostly relevant in the core of the waveguide.

The results are summarised in Table 5.3 for the single and double plane cases covered in Sections 4.2.1 and 4.2.2. The configurations with four and six contact planes only give small corrections compared to the two plane case, since the additional pressures are independent of the height. This should be compared with the gravitational effect


$$\;\;(5.5)$$

in the expected GRAVITES signal, and the hope is to attain an accuracy of 95 %. The control of the temperature and the pressure needed for the GRAVITES experiment is challenging but expected to be achievable.

**Table 5.3.** Figures for the GRAVITES experiment. The table lists the differences in the fields resulting from a difference of height between the arms of 1 m. The columns isolate the effects with respect to their sources. We write ${\delta \text{g}}_{\text{step}}^{\text{single}}$ for the effect calculated in the configuration of Section 4.2.1, ${\delta \text{g}}_{\text{line}}^{\text{single}}$ for the effect calculated in the configuration of Section 4.1.1, and ${\delta \text{g}}_{\text{step}}^{\text{double}}$ for that in Section 4.2.2; *δ* $\bar{\sigma }$ _*rr*_ is defined in ( 5.1); || *δu*|| _max_ is the maximal transverse deformation of the waveguide over the region *U*_1/2_ defined in Equation (4.53); *δL* is the change of length of the waveguide due to the Poisson effect; *δφ* is the change of phase of light due to the elastic elongation of the waveguide.

	${\text{δg}}_{\text{step}}^{\text{single}}$	${\delta \text{g}}_{line}^{\text{single}}$	${\delta \text{g}}_{\text{step}}^{\text{double}}$	*δ* = 10 Pa	*δT* = 10 ^–2^ K
*δ* $\bar{\sigma }$ _*rr*_[Pa]	2.1 · 10 ^–7^	2.1 · 10 ^–7^	2.9 · 10 ^–7^	10	0
|| *δu*|| _max_[µm]	8.5 · 10 ^–15^	7.2 · 10 ^–15^	9.9 · 10 ^–15^	9.9 · 10 ^–9^	2.0 · 10–7
*δL*[µm]	9.6 · 10 ^–8^	1.3 · 10 ^–7^	1.3 · 10 ^–7^	4.7	180
*δφ*[rad]	5.8 · 10 ^–7^	7.9 · 10 ^–7^	8.0 · 10 ^–7^	28	1080

Let us finally note that the height of the spools will be of the order of 30 cm, so there will be gradients of , *T*, and g along the waveguide which will influence the geometry of each arm. But, as already pointed out, in GRAVITES what matters is not the geometry itself but its change when an arm is displaced vertically. For this reason we expect that the models that we analysed suffice to provide a solid estimate of the influence of elastic deformations on GRAVITES.

It has been proposed to place the interferometer in a centrifuge, with horizontal plane of rotation, with the arms of the interferometer placed at different distances from the center of the centrifuge, achieving an acceleration gradient *δ*g *≈* 10 m/s ^2^ between the arms. To estimate the difference of phase between the arms we invoke the equivalence principle, modelling this problem by a radial gravitational field directed away from the axis of rotation of the centrifuge, with strength depending upon the distance from the axis, and ignoring the Earth gravitational field which acts with constant strength normal to the plane of rotation. (Clearly a more precise model would be needed for the real experiment. For example, the gradient of the “gravitational field” along the length of the spool is likely to become relevant; the deformations of the supporting elements might have to be taken into account.) For future reference, the corresponding values are given in Table 5.4; we do not include effects related to the change of temperature or pressure there, as such effects would depend strongly upon the experimental setup.

**Table 5.4.** Estimates for a centrifuge experiment with *δ*g = 10 m/s ^2^ at one meter separation. Notation as in Table 5.3.

	${\delta \text{g}}_{\text{step}}^{\text{single}}$	${\delta \text{g}}_{\text{step}}^{\text{double}}$
*δ* $\bar{\sigma }$ [Pa]	0.7	1.5
|| *δu*|| _max_[µm]	2.4 · 10 ^−8^	4.0 · 10 ^−8^
*δL*[µm]	0.3	0.7
*δφ*[rad]	1.9	4.2

### A   Relation between $\bar{P}$ and *κ*

The pressure terms $\bar{P}$ in the quadrilateral configuration, as well as $\hat{P}$ and $\overset{\vee }{P}$ in the hexagonal configuration are linked to the spooling tension. We make this relation explicit for the case of a waveguide pressing against a rigid cylinder of radius *R*.

We assume a static configuration, and we ignore boundary effects at the point where the contact between the waveguide and the spool is lost. We consider a waveguide, consisting of an optical fiber, which is wound *N* times in one layer around a rigid cylinder of radius *R*. We suppose that the contact interface of the waveguide and the cylinder has constant width *w*, and that the pressure, which we denote by $\bar{P}$ , exerted on the cylinder by the waveguide is constant across the whole area of contact. We assume that the axis of the cylinder is vertical, and we are interested in the pressure felt by the waveguide at the contact interface between the waveguide and the cylinder.

In a real-life situation there would be several layers of the waveguide, with various radii, with only the lowest one in contact with the cylinder, and the further ones in contact with neighbouring layers. Here we only consider the first layer, which is in contact with the cylinder. Similar considerations can be applied to describe successive layers; one would then also need to take into account the fact that the neighbouring layers are elastic and not rigid, as well as deformations of the neighbouring layers arising from the Poisson ratio. Our analysis in the main body of the paper sets the ground for further such investigations, which are left to future work.

**Figure A.1.**
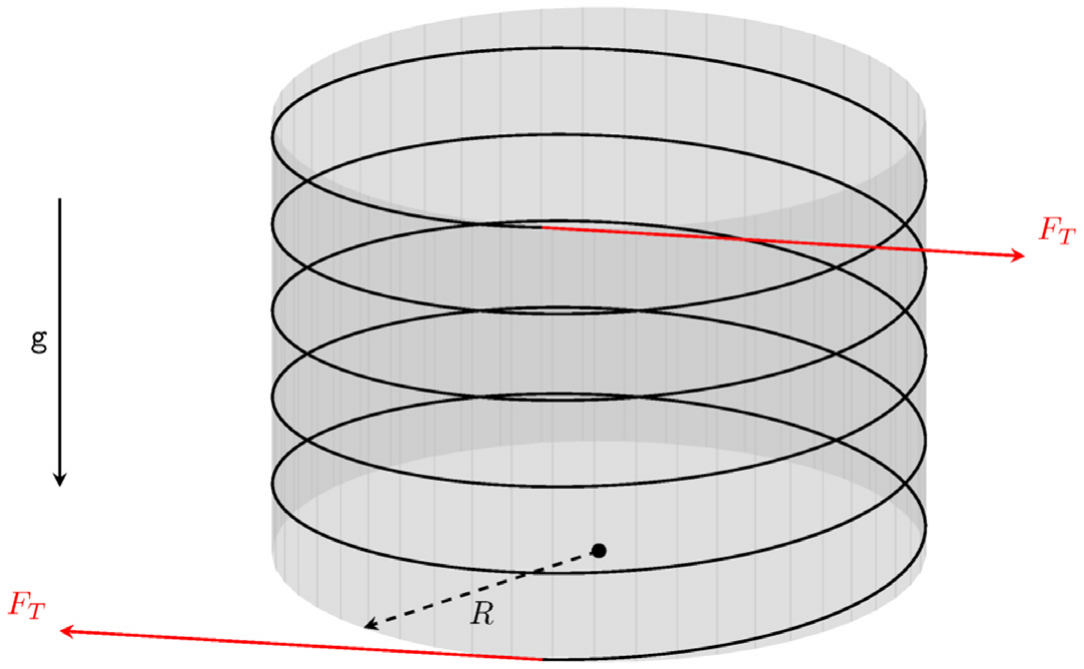
A waveguide wound around a rigid cylinder with radius *R*. We show a single layer of waveguide wrapped around the spool. Equal tension forces are applied at both the starting and end points so that the spool is in equilibrium. The spacing between the neighboring sections of the fiber has been increased for visual clarity.

**Figure A.2.**
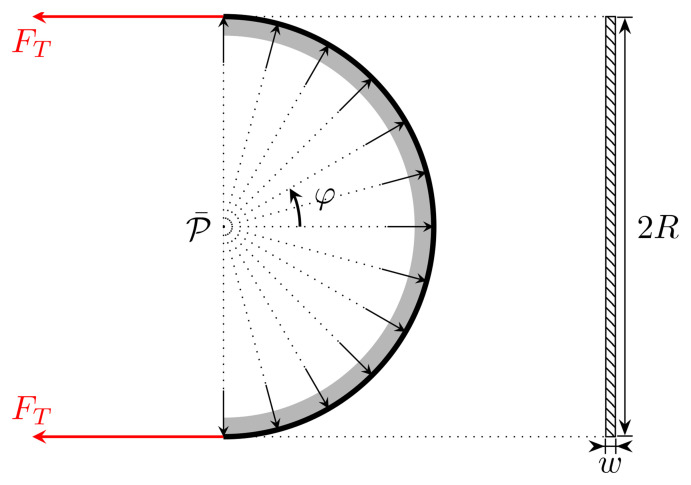
Free-body diagram of half of a single turn of waveguide around the spool (this corresponds to a view from above in Figure 1.3c or Figure A.1, so that the vertical is orthogonal to the plane of the picture). The rectangle on the right-hand side shows the contact area with the width *w* as viewed from the left. The arrows illustrate the direction of the (uniform) reaction force.

We relate the pressure $\bar{P}$ of Figure 1.3c to the tension force *F _T_* by considering the free-body diagram of a half of a single turn of a waveguide, i.e., we consider the intersection of the spool together with the waveguide and a plane on which the axis of the spool lies. The left Figure A.2 shows the intersection when viewed from above the spool of Figure A.1.

Let us imagine that we have a constant pressure $\bar{P}$ acting from the side as in Figure 1.3c, generating a force orthogonal to the contact region with the spool. This pressure generates a force *ℱ* which should equate the tension forces, i.e., *ℱ* = 2 *F _T_*, where


$$\;ℱ\;\text{:=}\;\int_{-\pi /2}^{\pi /2}\bar{P}\;\text{cos}\;\varphi \;A\;\text{d}\varphi =2Rw\;\int_{-\pi /2}^{\pi /2}\bar{P}\;\text{cos}\;\varphi \;\text{d}\varphi \;(\text{A.1})$$

is the normal force due to the pressure $\bar{P}$ acting on the contact area and *φ* is the angle shown in Figure A.2 cover 180 degrees of a semicircle. Hence, *ℱ* is the effective force caused by the pressure $\bar{P}$ .

We assume that the tension force is constant along the waveguide. Therefore, we find *ℱ* = 4 *Rw* $\bar{P}$ , which after equating the forces yields


$$\;\bar{P}=\frac{F_{T}}{2Rw}.\;(\text{A.2})$$

Recall that the constant appearing in the Hertz constant problem is defined as the total force per unit length pushing the bodies into each other, which in our context translates to $\bar{P}$ = / *w*. Comparing with ( A.2) we find


$$\;F=\frac{F_{T}}{2R}.\;(\text{A.3})$$

(Note that this is independent of the number of windings in the spool, and that a virtual work calculation gives the same result.) This value of can now be used in Section 4.2 to calculate *w* by solving the contact problem.

This formula is valid for the first layer, as counted starting from the core of the spool. One can generalize the result for the case where we have more layers that exert forces upon each other by writing


$$\;{\bar{P}}_{n}-{\bar{P}}_{n+1}=\frac{F_{T}}{2[R+(n-1)a]w_{n}},\;(\text{A.4})$$

where *w _n_* is the contact width at the *n*’th layer, while $\bar{P}$ _*n*_ and $\bar{P}$ _*n*+1_ are the contact pressures there.

### B   Extending to non-symmetric solutions

Although the mirror symmetry assumption made in Section 2 is well-motivated for our purposes, we nevertheless give the full solution for completeness, demanding only regularity at *r* = 0 and 2 *π*-periodicity in *θ*.

Discarding only terms incompatible with regularity and 2 *π*-periodicity in ( 2.26), as well as *B*_0_, *D*_1_ and *d*_1_ which do not contribute to the stresses, we have


$$\;\begin{array}{l} \phi (r,\theta )=D_{0}r^{2}+C_{1}r^{3}\text{cos(}\theta \text{)}+c_{1}r^{3}\text{sin(}\theta \text{)}\\ \;+\sum_{n\geq 2}\left[\left(A_{n}r^{n}+C_{n}r^{n+2}\right)\text{cos(}n\theta )+\left(a_{n}r^{n}+c_{n}r^{n+2}\right)\text{sin(}n\theta \text{)}\right].\;(\text{B.1}) \end{array}$$

Again, the displacement can be derived by integrating ( 2.22)–( 2.24), yielding


$$\;\begin{array}{l} u_{r}(r,\theta )=\frac{1}{2\mu }\{2(1-2\nu )D_{0}r-\frac{1}{2}\text{g}\rho (1-2\nu )r^{2}\text{cos}(\theta )+(1-4\nu )r^{2}(C_{1}\text{cos}(\theta )+c_{1}\;\text{sin}(\theta ))\\ \;+\sum_{n\geq 2}(\left[-nA_{n}r^{n-1}+(2-4\nu -n)C_{n}r^{n+1}\right]\text{cos(}n\theta \text{)}\\ \;+\left[-na_{n}r^{n-1}+(2-4\nu -n)c_{n}r^{n+1}\right]\text{sin(}n\theta \text{)})\}\\ \;-\text{Ξ}\;\text{cos(}\theta \text{)}+{\text{Ξ}}_{2}\text{sin(}\theta \text{)}-\nu \kappa r+(1+\nu )\alpha (T-T_{0})r,\;(\text{B.2}) \end{array}$$

$$\;\begin{array}{l} u_{\theta }(r,\theta )=\frac{1}{2\mu }\{\left(5-4\nu )r^{2}(C_{1}\;\text{sin}(\theta )+c_{1}\;\text{cos}(\theta ))-\frac{1}{2}\text{g}\rho (1-2\nu )r^{2}\;\text{sin}(\theta \right)\\ \;+\sum_{n\geq 2}(\left[nA_{n}r^{n-1}+(4-4\nu +n)C_{n}r^{n+1}\right]\text{sin(}n\theta \text{)}\\ \;\;+\left[-na_{n}r^{n-1}-(4-4\nu +n)c_{n}r^{n+1}\right]\text{cos(}n\theta \text{)})\}\\ \;+\text{Ξ}\;\text{sin(}\theta )+{\text{Ξ}}_{2}\text{cos(}\theta )+c^{*}r,\;(\text{B.3}) \end{array}$$

with the integration constants determined by the boundary conditions *u _r_* ( *a*, 0) = 0 = *u _θ_* ( *a*, 0) as above. Thus,


$$\;\begin{array}{l} \text{Ξ}=\frac{1}{2\mu }\{2(1-2\nu )D_{0}a+(1-4\nu )C_{1}a^{2}-\frac{1}{2}\text{g}\rho (1-2\nu )a^{2}\\ \;+\sum_{n\geq 2}\left[-nA_{n}a^{n-1}+(2-4\nu -n)C_{n}a^{n+1}\right]\}-\nu \kappa a+(1+\nu )\alpha (T-T_{0})a,\;(\text{B.4}) \end{array}$$

and further


$$\;{\text{Ξ}}_{2}=\frac{1}{2\mu }\left\{(5-4\nu )a^{2}c_{1}+\sum_{n\geq 2}\left[na_{n}a^{n-1}+(4-4\nu +n)c_{n}a^{n+1}\right]\right\}-c^{*}a,\;(\text{B.5})$$

provided that the sums converge.

Continuing to the boundary conditions, we immediately restrict to the frictionless case, taking


$$\;\sigma_{r\theta }{\text{|}}_{\partial U}=0.\;(\text{B.6})$$

The boundary condition for *σ _rr_* can now be a general Fourier series which we write in the suggestive form


$$\;\sigma_{rr}{\text{|}}_{\partial U}=f(\theta )=f_{0}+\sum_{n\geq 1}[(f_{-n}+f_{n})\text{cos}(n\theta )+i(f_{-n}-f_{n})\text{sin}(n\theta )],\;(\text{B.7})$$

with


$$\;f_{n}=\frac{1}{2\pi }\int_{-\pi }^{\pi }\text{d}\theta \;f\text{(}\theta \text{)}e^{in\theta }.\;(\text{B.8})$$

These boundary conditions determine the coefficients in ( B.1) algebraically by comparing to the corresponding stresses ( 2.15)–( 2.17). We find


$$\;C_{1}=c_{1}=0,\;C_{n}=\frac{1-n}{a^{2}(1+n)}A_{n}\;\text{and}\;c_{n}=\frac{1-n}{a^{2}(1+n)}a_{n},\;(\text{B.9})$$

via ( B.6), leading to


$$\;\sigma_{rr}{\text{|}}_{\partial U}=2D_{0}-a\text{g}\rho \;\text{cos(}\theta \text{)}\;+\sum_{n\geq 2}2(1-n)a^{n-2}[\text{sin}(n\theta )a_{n}+\text{cos}(n\theta )A_{n}],\;(\text{B.10})$$

and comparing with ( B.7) for the remaining coefficients


$$\;D_{0}=\frac{1}{2}f_{0},\;(B.11)$$

$$\;A_{n}=-\frac{1}{2(n-1)}a^{2-n}(f_{-n}+f_{n}),\;(B.12)$$

$$\;a_{n}=-\frac{i}{2(n-1)}a^{2-n}(f_{-n}-f_{n}),\;(B.13)$$

for *n* ≥ 2, as well as


$$\;f_{-1}+f_{1}=-a\text{g}\rho ,\;(B.14)$$

$$\;f_{-1}-f_{1}=0.\;(B.15)$$

Note that, similarly to the symmetric case the space of possible boundary conditions is constrained by the body force. Without any body forces oriented along the *x*-direction ( B.15) requires that the sin( *θ*) term in ( B.7) vanishes.

In the simplest case, considering line forces from the left and right and a line support from the bottom, i.e. boundary conditions


$$\;\sigma_{r\theta }\text{|}\partial U=0,\;(B.16)$$

$$\;\sigma_{rr}\text{|}\partial U=-P\delta (\theta )-\hat{P}\delta (\theta -\pi /2)-\overset{\vee }{P}\delta (\theta +\pi /2),\;(B.17)$$

Equation (B.15) implies $\hat{P}$ = $\overset{\vee }{P}$ , thus forcing mirror symmetry.

## Data Availability

No data are associated with this article.
