# Supplementary material for: On elastic deformations of cylindrical bodies under the influence of the gravitational field
Source: Open Res Eur. 2024 Dec 4;4:98. Originally published 2024 May 10. [Version 2] doi: 10.12688/openreseurope.17329.2 (PMC11367705; doi:10.12688/openreseurope.17329.2)
Supplement: Peer review history

# Reviewer report: Reviewer response for version 2

**DOI:** 10.21956/openreseurope.20364.r48085

**Published:** 2024-12-24

**Authors:** Ilya Svetlizky

**Affiliations:** 1 Physics department, Technion Israel Institute of Technology (Ringgold ID: 26747), Haifa, Haifa District, Israel

Review of “On elastic deformations of cylindrical bodies under the influence of the gravitational field” Barzegar et al.

 This paper explores the elastic deformation of optical waveguides under the combined influences of gravitational fields, temperature, and external pressure, motivated by experiments investigating the impact of gravity on photon propagation in optical fibers. The authors model the spooled waveguide as multiple straight and parallel cylinders. The contact between the cylinders is modeled by either a singular line or a more realistic uniformly distributed pressure. Using the classical Michell series, the authors obtain analytical estimates of the stresses within the waveguide and compare these to the numerical solutions based on the Hertzian contact boundary conditions. These findings hold significant importance for interpreting experimental data from the GRAVITES experiment.

 I find the manuscript to be well-written. The authors provide a comprehensive introduction—making the text self-contained—and a detailed discussion of the analysis, which appears to be sound. Additionally, the authors thoroughly address previous referees' constructive feedback, enhancing their work's robustness and clarity. For these reasons, I recommend this article for indexing.

Is the study design appropriate and does the work have academic merit?

Yes

Is the work clearly and accurately presented and does it cite the current literature?

Yes

If applicable, is the statistical analysis and its interpretation appropriate?

Not applicable

Are all the source data underlying the results available to ensure full reproducibility?

Yes

Are the conclusions drawn adequately supported by the results?

Yes

Are sufficient details of methods and analysis provided to allow replication by others?

Yes

Reviewer Expertise:

Experimental soft condensed matter physics

I confirm that I have read this submission and believe that I have an appropriate level of expertise to confirm that it is of an acceptable scientific standard.

# Reviewer report: Reviewer response for version 2

**DOI:** 10.21956/openreseurope.20364.r48026

**Published:** 2024-12-16

**Authors:** Matthew Maitra

**Affiliations:** 1 ETH Zurich, Zürich, Zurich, Switzerland

My thanks to the authors for making these revisions. Right now I have nothing more to add.

Is the study design appropriate and does the work have academic merit?

Yes

Is the work clearly and accurately presented and does it cite the current literature?

Partly

If applicable, is the statistical analysis and its interpretation appropriate?

Not applicable

Are all the source data underlying the results available to ensure full reproducibility?

No source data required

Are the conclusions drawn adequately supported by the results?

Yes

Are sufficient details of methods and analysis provided to allow replication by others?

Yes

Reviewer Expertise:

Theoretical continuum mechanics; theoretical seismology; magnetohydrodynamics

I confirm that I have read this submission and believe that I have an appropriate level of expertise to confirm that it is of an acceptable scientific standard.

# Reviewer report: Reviewer response for version 1

**DOI:** 10.21956/openreseurope.18729.r41793

**Published:** 2024-08-23

**Authors:** Michele Brun

**Affiliations:** 1 University of Cagliari, Cagliari, Italy

The paper presents some semi-analytical results for an elongated cylinder subjected to a transverse gravitational load and various contact forces.

 The study is motivated by the need to model a planned experiment that aims to explore the influence of the gravitational field on entangled photons propagating in waveguides.

 The approach used is classical and well-executed, determining the solutions in terms of Airy potentials. However, the advantage of this solution is currently limited by the availability of numerical techniques, particularly in the linear regime. Nonetheless, the work has its validity. The proposed results can be considered as an application of existing techniques to a specific problem.

 Overall, I believe the work deserves acceptance after the following minor revisions have been addressed:

1. The authors refer to large elastic deformations in the Background section, but the entire model has been developed in the small deformation (geometrically linear) regime.
2. Figure 1.1 is not sufficiently explanatory for a non-specialist, and additional details should be included. It is strongly recommended to visualize the reference system in most of the figures.
3. Page 4, Section 2: expression (2.1) is not specific to an isotropic homogeneous material, but rather to a linear isotropic material. Note that inhomogeneity only implies that material constants are functions of position.
4. The approximation indicated in Figure 4.5 is not validated. Would considering a piecewise constant approximation with additional subintervals for the pressure distribution improve the precision of the solution? In this regard, it should also be noted that the difference formula (4.50) is based on solutions that are relatively far from the load application neighborhoods.
5. I think that formula (4.64) is not correct since it appears has a linear momentum balance along y-direction and the components of the radial stresses resultants should be considered.
6. In eqns. (5.1) and (5.2) the notation @ is not explained.
7. In Appendix a the second \bar\mathcal{P} should be \hat\mathcal{P}.
8. I do not understand the factor 2 on the right handside of eqn. (A.1). Following classical Mariotte formula the 2 should be absent.
9. In the figure where contour plots of stress fields are shown legends should be inserted. These are of particular importance in Fig. 4.10.
  In conclusion I think that the work can be indexed after a revision.

Is the study design appropriate and does the work have academic merit?

Partly

Is the work clearly and accurately presented and does it cite the current literature?

Yes

If applicable, is the statistical analysis and its interpretation appropriate?

Not applicable

Are all the source data underlying the results available to ensure full reproducibility?

Yes

Are the conclusions drawn adequately supported by the results?

Yes

Are sufficient details of methods and analysis provided to allow replication by others?

Yes

Reviewer Expertise:

Solid Mechanics

I confirm that I have read this submission and believe that I have an appropriate level of expertise to confirm that it is of an acceptable scientific standard, however I have significant reservations, as outlined above.

# Response

**Published:** 2024-10-28

**Authors:** Elisabeth Steininger

**Affiliations:** Faculty of Physics, University of Vienna, Vienna, Vienna, Austria

We are grateful to the referee for his careful reading of our paper, and for helpful suggestions which led to significant improvements of our manuscript. We address the changes made in response to the comments (reproduced in italics) point by point:

**1)** *The authors refer to large elastic deformations in the Background section, but the entire model has been developed in the small deformation (geometrically linear) regime.*

**Author response: **We changed the background paragraph accordingly, and expanded it somewhat.

**2)** *Figure 1.1 is not sufficiently explanatory for a non-specialist, and additional details should be included. It is strongly recommended to visualize the reference system in most of the figures.*

**Author response: **We considerably expanded the caption following the recommendation. We are using the y-axis as the vertical direction in all the calculations, which we emphasised in the new caption, and we are using the y-axis accordingly in all Figures except Fig A.2.

 We have added the direction of the gravitational force in Figure 2.2 and A.1, and we propose not to add this information to those remaining Figures where it is not already available.

**3)** *Page 4, Section 2: expression (2.1) is not specific to an isotropic homogeneous material, but rather to a linear isotropic material. Note that inhomogeneity only implies that material constants are functions of position.*

**Author response: **(The equation has number 2.2 now) Removed the implication that the expression describes a homogeneous material. Stated afterwards that we restrict to homogeneous materials by taking the material parameters to be constant.   

**4)** *The approximation indicated in Figure 4.5 is not validated. Would considering a piecewise constant approximation with additional subintervals for the pressure distribution improve the precision of the solution? In this regard, it should also be noted that the difference formula (4.50) is based on solutions that are relatively far from the load application neighbourhoods.* **Author response: **We have added a comparison between the exact Hertz model, calculated by numerical methods and our step function approximation, which allows an explicit formula. A table and figure showing the differences has been added.  

**5)** *I think that formula (4.64) is not correct since it appears has a linear momentum balance along y-direction and the components of the radial stresses resultants should be considered.* **Author response: **(The equation has number 4.75 now) We have added an explicit calculation, which makes clear how the equations fits the radial stresses resultant; it was indeed confusing, as the cos of some angles involved are 1/2 or -1/2, and some of the pressures enter twice.  

**6)** *In eqns. (5.1) and (5.2) the notation @ is not explained.*

**Author response: **Deleted the @ notation since it is superfluous, and added an explanation of the notation used.  

**7)** *In Appendix a the second $\overline{\mathcal{P}}$ should be $\hat{\mathcal{P}}$.*

**Author response: **Fixed.  

**8)** *I do not understand the factor 2 on the right handside of eqn. (A.1). Following classical Mariotte formula the 2 should be absent.*

**Author response: **The factor 2 appears because $\mathcal F$ equals 2F _T_.  

**9)** *In the figure where contour plots of stress fields are shown legends should be inserted. These are of particular importance in Figure 4.10.*

**Author response: **The explanation of color coding in Figure 4.1 has been made clearer.

 We also added an explanation of the color coding in the caption of Figure 4.10.

# Reviewer report: Reviewer response for version 1

**DOI:** 10.21956/openreseurope.18729.r41521

**Published:** 2024-07-27

**Authors:** Matthew Maitra

**Affiliations:** 1 ETH Zurich, Zürich, Zurich, Switzerland

SUMMARY

 This paper investigates how the thermo-elastic deformation of an optical fibre will impact the phase-shift of photons passing through it. The paper supports the GRAVITES experiment -- a project that seeks to determine how Earth's gravity affects entangled photons -- by deriving some `extra' effects that will need to be corrected for when analysing the experimental data. 

 The GRAVITES experiment will essentially be an interferometer (placed in a vacuum chamber) whose two arms are optical fibres spaced a vertical metre apart. Entangled photons will pass through those arms, and the phase shift between the two arms will be measured. However, the two arms are expected to undergo different thermo-elastic deformations due to the differences in gravity, pressure and temperature between the arms. Crucially, this could lead to a change in the length of the arms, which would in turn lead to an extra phase-shift between the photons that would need to be corrected for. Ultimately, this change in length is the crucial quantity that the present paper seeks to derive.

 This paper therefore studies the thermo-elastic deformation of a long, homogenous, isotropic cylinder; the cylinder is clamped at one end and a force F_{T} is applied to the other, with the other external forces acting on the cylinder being ambient pressure and its own weight. The cylinder represents each of the arms of the interferometer. In reality the fibres will be spooled, but the authors argue (quite reasonably) that the radius of the spool will be so much larger than the the radius of the fibre that one may reasonably analyse instead the deformation of a long, straight cylinder subject to a `spooling tension force' (F_{T}).

 In Section 2 the authors state and solve the equations of static (thermo-)elastic deformation, first reducing the dimensionality of the problem by taking the cylinder's along-axis deformation to be precisely linear in z (eq. 2.5), then using the Airy stress-function to write down a general solution. In Section 3 they state the boundary conditions, as well as the crucial result (eq. 3.17) concerning the relationship between external parameters and the fibre's overall elongation. They match their solution to the boundary conditions for a variety of different representative geometries in Section 4, before discussing the application of their results to GRAVITES in Section 5.  

 The paper's language is clear and its analysis seems sound -- I appreciated Section 4's figures and adaptation of Hertz' method --  but I found the overall structure and aims quite hard to grasp. Multiple readings were required before I actually understood what Table 5.3 is saying (assuming I have now come to a correct understanding!). In what follows I will therefore mainly focus on structure and notation.

 DISCUSSION

 1

 Please lead the reader more clearly through the analysis, stating clearly why certain equations are being derived and certain assumptions made:

 1a

 As discussed above (and hopefully my interpretation is correct) the crucial relationship derived in the paper is eq.(3.17). But the authors do not flag the importance of this relationship explicitly, nor is eq.(3.17) even *referenced* in Section 5 when the GRAVITES estimates are made! Please make sure that this equation's importance is emphasised, and that its centrality to equations (5.1),(5.2),(5.3) -- and thus to the whole paper -- is stated.

 1b

 In that connection, please make EXPLICIT that Table 5.3 refers to DIFFERENCES BETWEEN THE RESPECTIVE INTERFEROMETER ARMS. It might be obvious after a few readings of the paper -- and I have little doubt that it is trivial to people who have immersed themselves in this topic for months -- but I don't think that the present manuscript makes clear what is being compared to what. I would appreciate something like the following to be added to the end of Section 3, after eq.(3.17):

 ``Given a spooling force and upon specifying ambient gravity, pressure and temperature, eq.(3.17) allows the computation of the elongation of an optical fibre, that is of each arm of the interferometer. Now, due to the arms' one-metre vertical spacing, the ambient variables could be different for each arm; the spooling force could be different too because two different spools will be used. We should therefore expect each arm of the interferometer to be of different length. This difference in length will lead to an extra phase-shift that must be corrected for and that is derived directly from eq.(3.17). In Section 4 we proceed to derive expressions for \sigma_{xx} and \sigma_{yy} in terms of ambient pressure and gravity, so that eq.(3.17) can indeed be seen to express a relationship between each arm's actual length and: p,T,g and F_{T}.''

 If words to this effect had been included in the manuscript (perhaps even in the Introduction) I think I would have had a *much* easier time understanding where things were going.

 1c

 Please introduce and describe the spooling force F_{T} earlier in the main text, rather than just mentioning it in passing in the caption of Figure 2.1c. Preferably, state at the beginning of Section 2 that the model of the waveguide that this paper analyses in detail is the version from Figure 2.1c, and that we therefore have displacement-free boundary conditions on one end while needing to match the stress at the other end to the externally imposed force F_{T}. Incidentally, this would give a much clearer motivation for imposing eq.(2.5) than what the manuscript offers at present. 

 1d 

 Following on directly from 1c, please motivate eq.(2.5) more clearly (I didn't find the statement "compare Figure 2.1 (a)–(c)" to help much in that regard). Once the spooling force has been foregrounded, this should be easy. In addition, I would appreciate a note after eq.(2.10) stating that the z-component of the force-balance equation is automatically satisfied.

 1e (miscellaneous)

 Eq.(2.1): 

 What are \sigma and \epsilon?! A reader without much background in elasticity would be stuck here; please define all terms used in the paper. In addition, eq.(2.6) should logically precede eq.(2.1): how can you define strain without any concept of deformation? Also, the material need not be homogeneous for eq.(2.1) to hold.

 Eq.(2.11): 

 \sigma_{rr} + \sigma_{\theta\theta} should probably read \sigma_{xx} + \sigma_{yy} because the cylindrical coordinate system has not yet been introduced.

 Eq.(4.32):

 Please define both `A' and the concept of `indicator function'. (Presumably A here is different from the A in equations (4.10), (4.11) and (4.12)?!)

 Eq.(5.3): 

 \delta\overline{\sigma}_{\theta\theta} has not yet been formally defined. Eq.(5.1) should be adjusted to include this definition.

 2

 Temperature and thermo-elasticity.

 A temperature T_{0} is introduced in eq.(2.3) as a `baseline', with T being the actual (constant) temperature of the optical fibre. Assuming that \delta{T} in Table 5.3 represents the difference between the *actual* temperatures of the interferometer's respective arms, then eq.(5.3) should have (T-T_{0}) replaced by \delta{T}. (This caused me a lot of confusion at first!)

 3

 I would like to see more uncertainty estimates, please.

 3a

 What is the rough uncertainty on the spooling force. How different will it therefore be between the two arms?

 3b

 What is the rough uncertainty on phase-shift due to gravity (eq. 5.5)? Without that, it is tough to get a feel for the importance of the numbers in Table 5.3.  

 3c (tangential but related)

 Please could there be a little discussion on the practical feasibility of controlling \delta{T} and \delta{p} such that one can measure \delta\phi_{GRAVITES} with sensible uncertainty?

 3d (another tangent)

 I would also like to see a discussion of the practical feasibility of controlling vertical vs horizontal gradients of T and p. For the purposes of Section 2, 3 and 4's analysis, T is taken as constant. Yet in Section 5 it is allowed to vary vertically so as to be different for each arm. But if T is constant along the fibre, then in real life it must also be constant along the spool -- and that would seem to rule out vertical gradients. Essentially we seem to require that the temperature not vary vertically along the length scale of the spool, but that it potentially vary vertically between the interferometer's arms. Is this a contradiction? In this connection, it would be nice to know roughly how tall the spools will be.

 4

 Miscellaneous

 4a

 I do not agree that ``[l]arge elastic deformations of gravitating cylindrical bodies play a significant role in everyday life". Spherical bodies, yes. But cylindrical ones... surely not?! Is it simply a misprint, with the authors actually wishing to state the opposite? That would make better sense to me, given that the following sentence starts by drawing a contrast (``On the other hand [...]"). Whatever the case, I really think that this first sentence must be changed or removed. 

 4b

 It is not (necessarily) the problem of these authors, but does Fig. 1.1 really need that background that seems to be representing curved spacetime? It makes the cartoon rather hard to read. How are the two spools distributed in space with respect to each other? A background that helps to show that distribution would be much more helpful, at least to me.

 4c

 In fact, Figure 1.1 is not particularly helpful on its own. It is appealing as a cartoon visualising GRAVITES, but I would also appreciate a proper diagram that shows the effective setup analysed within the paper. That is, I would like to see two parallel lines whose spacing is clearly indicated, each of which has its own environmental parameters (p, T, g) associated with it, with coordinate systems fully marked etc. 

 4d

 Very last paragraph:

 The authors state that the centrifuge setup would require a more precise model. Perhaps they could outline what aspects of the present paper's analysis would need to be changed in that case? In particular, would it still be reasonable to assume a certain along-fibre deformation and then take all other fields to depend only on coordinates tangential to the fibre?

 CONCLUSION

 I will be glad to see this interesting paper (and its promised follow-up!) accepted for official approval subject to dealing with the points discussed above.

Is the study design appropriate and does the work have academic merit?

Yes

Is the work clearly and accurately presented and does it cite the current literature?

Partly

If applicable, is the statistical analysis and its interpretation appropriate?

Not applicable

Are all the source data underlying the results available to ensure full reproducibility?

No source data required

Are the conclusions drawn adequately supported by the results?

Yes

Are sufficient details of methods and analysis provided to allow replication by others?

Yes

Reviewer Expertise:

Theoretical continuum mechanics; theoretical seismology

I confirm that I have read this submission and believe that I have an appropriate level of expertise to confirm that it is of an acceptable scientific standard, however I have significant reservations, as outlined above.

# Response

**Published:** 2024-10-28

**Authors:** Elisabeth Steininger

**Affiliations:** Faculty of Physics, University of Vienna, Vienna, Vienna, Austria

We are grateful to the referee for his careful reading of our paper, and for helpful suggestions which led to significant improvements of our manuscript. We address the changes made in response to the comments (reproduced in italics) point by point:

**1)** *Please lead the reader more clearly through the analysis, stating clearly why certain equations are being derived and certain assumptions made:*

**Author response: **We have tried to address this in our corrections, detailed below, and in several comments added in the text.  

**1a)** As discussed above (and hopefully my interpretation is correct) the crucial relationship derived in the paper is eq.(3.17). But the authors do not flag the importance of this relationship explicitly, nor is eq.(3.17) even *referenced* in Section 5 when the GRAVITES estimates are made! Please make sure that this equation's importance is emphasized, and that its centrality to equations (5.1),(5.2),(5.3) -- and thus to the whole paper -- is stated.

**Author response: **We have emphasized the importance of eq. (3.17) further in the text and added a reference to it for eq. (5.3).  

**1b)** In that connection, please make EXPLICIT that Table 5.3 refers to DIFFERENCES BETWEEN THE RESPECTIVE INTERFEROMETER ARMS. It might be obvious after a few readings of the paper -- and I have little doubt that it is trivial to people who have immersed themselves in this topic for months -- but I don't think that the present manuscript makes clear what is being compared to what. I would appreciate something like the following to be added to the end of Section 3, after eq (3.17): "Given a spooling force and upon specifying ambient gravity, pressure and temperature, eq.(3.17) allows the computation of the elongation of an optical fiber , that is of each arm of the interferometer. Now, due to the arms' one-metre vertical spacing, the ambient variables could be different for each arm; the spooling force could be different too because two different spools will be used. We should therefore expect each arm of the interferometer to be of different length. This difference in length will lead to an extra phase-shift that must be corrected for and that is derived directly from eq.(3.17). In Section 4 we proceed to derive expressions for σ _xx_ and σ _yy_ in terms of ambient pressure and gravity, so that eq.(3.17) can indeed be seen to express a relationship between each arm's actual length and: p,T,g and F _T_." If words to this effect had been included in the manuscript (perhaps even in the Introduction) I think I would have had a *much* easier time understanding where things were going.

**​​​​​​​Author response: **Added some clarification following eq. (3.17) as to the relation with the following section.

 Additionally added a short remark before eq. (5.1) and in the caption to clarify that we are interested in differences of configurations for the GRAVITES experiment, emphasizing that we compare two fibers with some vertical separation.

 Added in the introduction a paragraph in the spirit suggested.   

**1c.i)** *Please introduce and describe the spooling force F _T_ earlier in the main text, rather than just mentioning it in passing in the caption of Figure 2.1c. Preferably, state at the beginning of Section 2 that the model of the waveguide that this paper analyses in detail is the version from Figure 2.1c, and that we therefore have displacement-free boundary conditions on one end while needing to match the stress at the other end to the externally imposed force F _T_. Incidentally, this would give a much clearer motivation for imposing eq.(2.5) than what the manuscript offers at present.*

**​​​​​​​Author response: **Added some explanation for F _T_ to the caption of Figure 2.1, with reference to Section 3 where it is thoroughly discussed.

 Further added some comments in the main text. 

 Note that the analysis in our work can be applied to any of the configurations pictured in Figure 2.1.  

**1c.ii)** *Incidentally, this would give a much clearer motivation for imposing eq.(2.5) than what the manuscript offers at present.*

**​​​​​​​Author response: **Equation (2.5) ((2.6) in the revised version) is unavoidable if one wants to take into account the three-dimensional Poisson effect, while remaining compatible with a basically 2-dimensional description used in our calculation.  

**1d.i)** *Following on directly from 1c, please motivate eq.(2.5) more clearly (I didn't find the statement "compare Figure 2.1 (a)–(c)" to help much in that regard). Once the spooling force has been foregrounded, this should be easy.*

**​​​​​​​Author response: **(Hopefully this has been taken care of by our previous additions)  

**1d.ii)** *In addition, I would appreciate a note after eq.(2.10) stating that the z-component of the force-balance equation is automatically satisfied.*

**​​​​​​​Author response: **Comment added.  

**1e.i)** *Eq.(2.1): What are σ and ϵ?! A reader without much background in elasticity would be stuck here; please define all terms used in the paper. In addition, eq.(2.6) should logically precede eq.(2.1): how can you define strain without any concept of deformation? Also, the material need not be homogeneous for eq.(2.1) to hold.*

**​​​​​​​Author response: **Text rearranged, defined the stress and strain tensors in the text.

 Added a comment that we restrict to constant material parameters and thus homogeneous materials.  

**1e.ii)** *Eq. (2.11): σ _rr_ + σ _θθ_ should probably read σ _xx_ + σ _yy_ because the cylindrical coordinate system has not yet been introduced.*

**​​​​​​​Author response: **Fixed.  

**1e.iii)** *Eq.(4.32): Please define both `A' and the concept of `indicator function'. (Presumably A here is different from the A in equations (4.10), (4.11) and (4.12)?!) * Changed the previous symbol A to Ω to make clear that there is no relation to Eq. (4.10)-(4.12).

**​​​​​​​Author response: **Removed the notion `indicator function'.  

**1e.iv)** *Eq.(5.3): *�� *σ _θθ_ **has not yet been formally defined. Eq.(5.1) should be adjusted to include this definition.*

**​​​​​​​Author response: **Added a reference to �� *σ _θθ_*.  

**2)** *Temperature and thermo-elasticity. A temperature T _0_ is introduced in eq.(2.3) as a `baseline', with T being the actual (constant) temperature of the optical fiber . Assuming that *�� T in Table 5.3 represents the difference between the *actual* temperatures of the interferometer's respective arms, then eq.(5.3) should have (T-T _0_) replaced by �� *​​​​​​​T. (This caused me a lot of confusion at first!)*

**​​​​​​​Author response: **Fixed.  

**3)** *I would like to see more uncertainty estimates, please.*

**​​​​​​​Author response: **Added.  

**3a)** *What is the rough uncertainty on the spooling force. How different will it therefore be between the two arms?* Added the uncertainty.

**​​​​​​​Author response: **Added an explanation that we assume that the forces in both arms are the same and that they do not change when an arm is moved in the gravitational field.  

**3b)** *What is the rough uncertainty on phase-shift due to gravity (eq. 5.5)? Without that, it is tough to get a feel for the importance of the numbers in Table 5.3.*

**​​​​​​​Author response: **Added (expected accuracy is 95%).  

**3c)** *(tangential but related) Please could there be a little discussion on the practical feasibility of controlling *�� *T and *�� *p such that one can measure *�� *ϕ _GRAVITES_ with sensible uncertainty?*

**​​​​​​​Author response: **Comments added.  

**3d)** *I would also like to see a discussion of the practical feasibility of controlling vertical vs horizontal gradients of T and p. For the purposes of Section 2, 3 and 4's analysis, T is taken as constant. Yet in Section 5 it is allowed to vary vertically so as to be different for each arm. But if T is constant along the fiber , then in real life it must also be constant along the spool -- and that would seem to rule out vertical gradients. Essentially we seem to require that the temperature not vary vertically along the length scale of the spool, but that it potentially vary vertically between the interferometer's arms. Is this a contradiction? In this connection, it would be nice to know roughly how tall the spools will be.*

**​​​​​​​Author response: **Comments added: the spools are expected to be of the order of 30 cm, so there will be a gradient which will have a nontrivial effect on each arm. But in GRAVITES what matters is not the geometry but its change when the arm is moved up, therefore we expect that our model should suffice to give a good estimates of the effects on GRAVITES. 

 One way of mitigating the results of the deformations predicted by our calculations will be to place each arm in a separate vacuum chamber. This is one of the configurations that is being explored now.   

**4a)** *I do not agree that ``[l]arge elastic deformations of gravitating cylindrical bodies play a significant role in everyday life". Spherical bodies, yes. But cylindrical ones... surely not?! Is it simply a misprint, with the authors actually wishing to state the opposite? That would make better sense to me, given that the following sentence starts by drawing a contrast (``On the other hand [...]"). Whatever the case, I really think that this first sentence must be changed or removed.*

**​​​​​​​Author response: **Sentence removed.  

**4b)** *It is not (necessarily) the problem of these authors, but does Fig. 1.1 really need that background that seems to be representing curved spacetime? It makes the cartoon rather hard to read. How are the two spools distributed in space with respect to each other? A background that helps to show that distribution would be much more helpful, at least to me.* **​​​​​​​Author response: **Added a comment regarding the background of Fig. 1.1 in its caption and expanded on the description.  

**4c)** *In fact, Figure 1.1 is not particularly helpful on its own. It is appealing as a cartoon visualising GRAVITES, but I would also appreciate a proper diagram that shows the effective setup analysed within the paper. That is, I would like to see two parallel lines whose spacing is clearly indicated, each of which has its own environmental parameters (p, T, g) associated with it, with coordinate systems fully marked etc.*

**​​​​​​​Author response: **Figure revised, and a second figure added.  

**4d)** *Very last paragraph: The authors state that the centrifuge setup would require a more precise model. Perhaps they could outline what aspects of the present paper's analysis would need to be changed in that case? In particular, would it still be reasonable to assume a certain along-fiber  deformation and then take all other fields to depend only on coordinates tangential to the fiber?*

**​​​​​​​Author response: **​​​​​​​Some comments added.

# Reviewer report: Reviewer response for version 1

**DOI:** 10.21956/openreseurope.18729.r41517

**Published:** 2024-07-19

**Authors:** Robert Beig

**Affiliations:** 1 Gravitational Physics/Physics Faculty, Universitat Wien, Vienna, Austria

An experiment is currently developed aiming at measuring the influence of gravity on entangled states of photons in an optical fiber. Gravity will also deform the shape of the waveguide, which potentially changes the phase shift to be measured in the experiment. The paper by Barzegar .,et al calculates these elastic deformations, using linearized (thermo-) elasticity for isotropic, homogenous materials - which should be a valid approximation under present circumstances. The main issue in their calculations comes from boundary conditions at the rigid plane where the cylindrical wave guide is placed. In a first step the authors assume this contact to occur across a line. The results in that case, as the authors point out, and as is known from the literature they cite, is 'unphysical' - by which they mean that the obtained deformation diverges at the contact interfaces. In a second part the authors improve these solutions by employing Hertz-type boundary conditions at these interfaces. Finally, in section 5, the authors, estimate the influence on elastic deformations on the planned GRAVITES experiment. They expect this influence on the measured phase shift to be 2 orders of magnitude smaller than that due to electromagnetism. More details are promised from electromagnetic calculations under way.

 I find the topic of this paper timely. The paper is well written, Without having checked details, I expect the calculations to be correct.

 I would like to raise one point concerning the presentation. Due to symmetry the work in this paper takes place in 2d. Here, if the external force field. Comes from a potential which is harmonic (as to good approximation is the case for terrestrial gravity) and temperature is constant, the field equations boil down to the biharmonic equation for a scalar called an Airy function in the literature plus suitable boundary conditions. (One can then use an explicit expression for the general solution to the latter equation, Fourier expanded with respect to angle, known as the Michell solution in the literature.) It would greatly benefit a general theoretical physics audience if the authors cared to spend a few lines explaining the process leading from the standard field equations to the biharmonic equation rather than only referring to the classical, engineering-type literature. The basic observation (apparently due to Airy) is that a divergence-free symmetric tensor in 2d can be written as. 

 σ _ij_ = ⸹ _ij_ ΔΦ - ∂ _i_∂ _J_Φ

 The authors might also mention that the 'compatibility condition' leading from there to the biharmonic equation is actually a purely geometric condition on a symmetric tensor in order to be the linearized strain tensor of a deformation.

Is the study design appropriate and does the work have academic merit?

Yes

Is the work clearly and accurately presented and does it cite the current literature?

Yes

If applicable, is the statistical analysis and its interpretation appropriate?

Not applicable

Are all the source data underlying the results available to ensure full reproducibility?

Yes

Are the conclusions drawn adequately supported by the results?

Yes

Are sufficient details of methods and analysis provided to allow replication by others?

Yes

Reviewer Expertise:

General Relativity, Relativistic Elasticity

I confirm that I have read this submission and believe that I have an appropriate level of expertise to confirm that it is of an acceptable scientific standard.

# Response

**Published:** 2024-10-28

**Authors:** Elisabeth Steininger

**Affiliations:** Faculty of Physics, University of Vienna, Vienna, Vienna, Austria

We are grateful to the referee for his careful reading of our paper, and for helpful suggestions which led to significant improvements of our manuscript. We address the changes made in the revision as a response to the comments (reproduced in italics) point by point:

- *It would greatly benefit a general theoretical physics audience if the authors cared to spend a few lines explaining the process leading from the standard field equations to the biharmonic equation rather than only referring to the classical, engineering-type literature. The basic observation (apparently due to Airy) is that a divergence-free symmetric tensor in 2d can be written as  σij = ⸹ij ΔΦ - ∂i∂jΦ.*

We added the geometric version of the equation defining the Airy stress function, to make the manuscript easier to read, with suitable comments.

- *The authors might also mention that the 'compatibility condition' leading from there to the biharmonic equation is actually a purely geometric condition on a symmetric tensor in order to be the linearized strain tensor of a deformation.*

It is our understanding that the referee meant that the "compatibility condition" for the Airy stress function is a geometric condition related to the vanishing, in the linear regime, of the Riemann tensor of the pullback of the metric from the spacetime manifold to the body manifold. We have added references to the literature on the subject in the introduction, but explaining all this might create confusion, as this is not necessary in our context.
